# *Prototaxites* fossils are structurally and chemically distinct from extinct and extant Fungi

**DOI:** 10.1126/sciadv.aec6277

**Published:** 2026-01-21

**Authors:** Corentin C. Loron, Laura M. Cooper, Sean McMahon, Seán F. Jordan, Andrei V. Gromov, Matthew Humpage, Niall Rodgers, Laetitia Pichevin, Hendrik Vondracek, Ruaridh Alexander, Edwin Rodriguez Dzul, Alexander T. Brasier, Michael Krings, Alexander J. Hetherington

**Affiliations:** ^1^ UK Centre for Astrobiology, School of Physics and Astronomy, University of Edinburgh, Edinburgh EH9 3FD, UK.; ^2^ Institute of Molecular Plant Sciences, School of Biological Sciences, University of Edinburgh, Max Born Crescent, Edinburgh EH9 3BF, UK.; ^3^ School of Geosciences, University of Edinburgh, Edinburgh EH9 3FE, UK.; ^4^ Life Sciences Institute, School of Chemical Sciences, Dublin City University, Dublin 9, Ireland.; ^5^ EastCHEM, School of Chemistry, University of Edinburgh, Edinburgh EH9 3FJ, UK.; ^6^ Northern Rogue Studios, 58 Towerhill Avenue, Cradlehall, Inverness IV2 5FB, UK.; ^7^ Beamline B22, Diamond Light Source, Harwell Science and Innovation Campus, Fermi Ave, Didcot OX11 0DE, UK.; ^8^ School of Geosciences, University of Aberdeen, Aberdeen AB24 3UE, UK.; ^9^ SNSB-Bayerische Staatssammlung für Paläontologie und Geologie, Richard-Wagner-Straße 10, 80333 Munich, Germany.; ^10^ Department für Geo- und Umweltwissenschaften, Paläontologie und Geobiologie, Ludwig-Maximilians-Universität, Richard-Wagner-Straße 10, 80333 Munich, Germany.; ^11^ Royal Botanic Garden Edinburgh, Edinburgh EH3 5LR, UK.; ^12^ Department of Natural Sciences, National Museums Scotland, Edinburgh EH1 1JF, UK.

## Abstract

*Prototaxites* was the first giant organism to live on the terrestrial surface, represented by columnar fossils of up to eight meters from the Early Devonian. However, its systematic affinity has been debated for over 165 years. There are now two remaining viable hypotheses: *Prototaxites* was either a fungus, or a member of an entirely extinct lineage. Here, we investigate the affinity of *Prototaxites* by contrasting its organization and molecular composition with that of Fungi. We report that fossils of *Prototaxites taiti* from the 407-million-year-old Rhynie chert were chemically distinct from contemporaneous Fungi and structurally distinct from all known Fungi. This finding casts doubt upon the fungal affinity of *Prototaxites*, instead suggesting that this enigmatic organism is best assigned to an entirely extinct eukaryotic lineage.

## Teaser

*Prototaxites* fossils are distinct from Fungi, suggesting that they represent an extinct lineage of eukaryotic life.

## INTRODUCTION

The *Prototaxites* fossil record spans the late Silurian to the Late Devonian (420 to 370 million years ago) (*1*–*23*), an interval of remarkable change on the terrestrial surface, marked by the diversification and rise to dominance of land plants, terrestrial animals and Fungi (*24*). *Prototaxites* was the largest terrestrial organism for much of this time (*1*), only overtaken in stature by large trees in the Middle to Late Devonian. Because of its large size, prevalence in many terrestrial localities (*1*, *10*), and direct evidence of its use as a food source by arthropods (*1*, *25*–*27*), *Prototaxites* undoubtedly played a key ecological role during terrestrialization. Despite its importance, the systematic affinity of *Prototaxites* has remained unsolved for over 165 years (*1*–*12*). Its complexity supports a eukaryote affinity, but assignments to algal groups or land plants (*2*–*5*, *8*, *9*) have been repeatedly ruled out (*1*, *7*, *11*–*16*). δ^13^C values of *Prototaxites* fossils are inconsistent with a solely photosynthetic metabolism (*13*, *14*), and while there has been a persistent hypothesis that *Prototaxites* derived energy through a lichen-style symbiosis with a photobiont to achieve such large structures in ecosystems with sparse decayed organic matter (*17*–*19*), there has been no convincing evidence of a photobiont population, even in well preserved samples (*6*, *7*). Therefore, in the absence of strong evidence supporting autotrophy or mixotrophy, there is a growing consensus that *Prototaxites* was heterotrophic, possibly saprotrophic (*7*, *13*, *14*). This evidence, and that *Prototaxites* fossils are constituted entirely of interwoven masses of tubes (*1*), has fueled arguments for its classification as a member of the Fungi. A fungal interpretation of *Prototaxites* has become most widespread in the last 25 years (*1*, *6*, *17*–*22*), with attempts to assign it to extant groups including the Ascomycota (*6*), Basidiomycota (*1*), and Mucoromycota (*18*). However, the unique combination of characters seen in *Prototaxites*, which is not known from any extant fungal group (*23*), makes each of these assignments debatable. The field now faces two leading hypotheses: *Prototaxites* was either a fungus or a member of a now entirely extinct lineage (*7*, *16*, *23*). Further insight into these competing hypotheses requires continued fossil discovery and application of novel techniques.

**Fig. 1.**
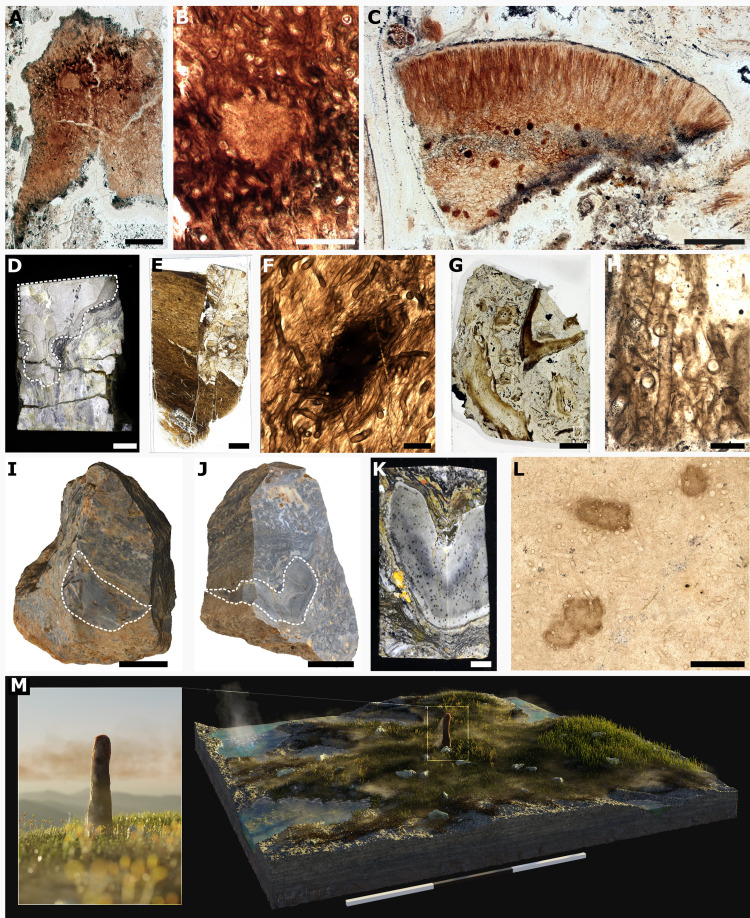
*P. taiti* material from the Rhynie chert. (**A** to **C**) Images of two of the four thin sections containing the fragments that constitute the *P. taiti* type material, including the fragment with medullary spots (B) and peripheral region (C). (D to L) *P. taiti* material used in this study. (**D** and **E**) Lyon 156 with *P. taiti* highlighted in dashed lines. (E) Thin section produced from the block in (D) showing the fractured *P. taiti* specimen. (**F**) Magnified image of the thin section in (E) showing the characteristic tubes and medullary spots of *P. taiti*. (**G** and **H**) Thin section made from Lyon 48 with *P. taiti* in dashed box. (H) Detail of thin section in (G) showing the tubes. (I to M) Imaging and reconstruction of a large, exceptionally well-preserved *P. taiti* from NSC.36. (**I**) Photogrammetry model of NSC.36 before cutting with surface exposed *P. taiti* circled by dashed line. (**J**) Photogrammetry model of NSC.36 after initial cutting of the block with *P. taiti* circled by a dashed line. (**K**) Block of NSC.36 from which thin sections were produced, showing medullary spots throughout the body. (**L**) Thin section taken from the block in (K) showing characteristic tubes and medullary spots of *P. taiti.* (**M**) Artist reconstruction of *P. taiti* within the Rhynie ecosystem including hypothesized reconstruction of the aerial portion. Illustration by M. Humpage, Northern Rogue Studios. Scale bars: 3 m (M), 3 cm (I), 2 cm (J), 1 cm (D), 5 mm (E, G, and K), 1 mm (C), 500 μm (A and L), 200 μm (B and F), and 100 μm (H). Specimen accession codes: GLAHM Kid 2523 (A and B), GLAHM Kid 2525 (C), Lyon 156 (D), Lyon 156 MPEG0078 (E and F), NSC.36 (I to K), and NMS G.2024.5.7 (L).

How new fossils and techniques can influence this debate is demonstrated in the recent reinvestigation of one species of *Prototaxites*, *Prototaxites taiti*, from the exceptionally preserved Early Devonian Rhynie chert (*6*). *P. taiti* was originally described (*24*) from two fragments (Fig. 1, A to C), one containing medullary spots, dark spherical structures diagnostic of many *Prototaxites* species (Fig. 1, A and B), and another described as part of a peripheral region (Fig. 1C). A reinvestigation (*6*) of this peripheral fragment using confocal laser scanning microscopy (CLSM) led to its interpretation as a reproductive structure containing asci, therefore *P. taiti* was assigned to the Ascomycota, as a “basal member.” This assignment remains contentious, as *P. taiti* exhibits a combination of characters not seen in the Ascomycota or other extant fungal lineage (*23*), and most critically, the proposed asci (Fig. 1C) lack organic connection to material diagnostic of *P. taiti* (Fig. 1, A and B); therefore, we agree with Edwards (*28*) that these fragments should be considered separately. Despite this, the study showcases the role of *P. taiti* in forwarding the debate around the affinity of *Prototaxites*, due to its exceptional preservation. In addition, the Rhynie chert, one of the most important sites for fossil Fungi, is the ideal locality for comparisons between *Prototaxites* and Fungi.

Here, we report a macroscale *P. taiti* specimen from the Rhynie chert (Fig. 1, I to M). This specimen is exceptionally preserved in three dimensions and provided sufficient material for microscopy and molecular analyses, the latter achieved by a side-by-side comparison of the molecular fingerprint of *P. taiti* with those of Fungi and other taxa in the Rhynie chert, which have experienced the same diagenetic history. Our integrative approach, combining study of the molecular composition, organization, and anatomy of *P. taiti*, undermines the hypothesis that *P. taiti* was an ascomycete specifically, and a crown-group Fungus more generally, and instead supports assignment of *Prototaxites* to a previously undescribed extinct eukaryotic lineage.

## RESULTS AND DISCUSSION

### NSC.36 is the largest *Prototaxites* specimen known from the Rhynie chert

Our investigation of *P. taiti* draws upon three specimens: a large but highly fractured specimen in block Lyon 156 described in Edwards *et al.* (*25*) (Fig. 1, D to F), a small fragment of *P. taiti* in block Lyon 48 (Fig. 1, G and H), and a large and exceptionally well-preserved specimen in block NSC.36 (Fig. 1, I to L); the latter specimen (referred to as NSC.36) will be described here.

NSC.36 in the intact block was roughly cylindrical, 5.6 cm at its widest, and extended obliquely through the entirety of the block (6.9 cm). To our knowledge this is the largest *P. taiti* specimen reported from the Rhynie chert, and as it was incomplete at both ends, we infer that *P. taiti* was the largest known contiguous organism in the Rhynie ecosystem (Fig. 1M).

**Fig. 2.**
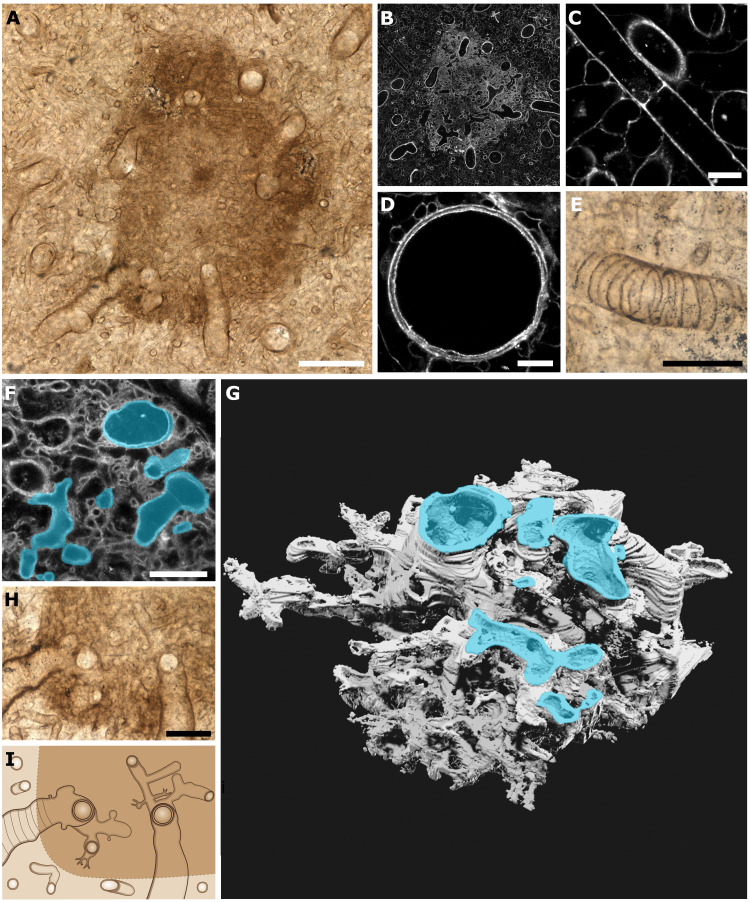
The medullary spots and tube types of *P. taiti* are morphologically distinct from extinct or extant fungal groups. (**A**) Transmitted light image showing a medullary spot within the body of *P. taiti*. (**B**) The same medullary spot imaged using CLSM, showing the spot to be composed of densely packed fine tubes contrasting with the less densely packed body. (**C** to **E**) Details of tubes types 1 to 3 seen in the body of *P. taiti*: a small diameter type 1 tube with a septal pore (C), a larger diameter type 2 tube (D), and a type 3 tube with annular thickenings (E). (**F** to **H**) Airyscan CLSM three-dimensional imaging reveals that in the medullary spot region all tube types are connected through a highly branched network. Tubes of a variety of morphologies (highlighted in cyan in F and G) were found to be connected to each other in a dense and fine branching network through the construction of a 3D model (G) using Airyscan CLSM z-stack data (the first image in the stack is shown in F). Examination of the spot region (H) supports the interconnection of all tube types through fine branching at the medullary spots, as shown in the schematic in (**I**). Scale bars: 100 μm (A), 50 μm (E and H), 20 μm (F), and 10 μm (C and D). Specimen accession code: NMS G.2024.5.7.

The block was initially bisected, revealing NSC.36 to be a boomerang shape in transverse section (Fig. 1J), the longer arm 3.9 cm long, and the other 3.6 cm. Orientation of the specimen was determined using geopetal features, and we found no evidence for compression or distortion of the specimen suggesting this was its original form (fig. S1 and Supplementary Text). Internally, NSC.36 comprises a light brown body with abundant and evenly distributed, dark brown, roughly spherical medullary spots measuring 200 to 600 μm in diameter (Fig. 1, K and L). A black carbonized layer delineates the specimen from the surrounding substrate (Fig. 1K), where remains of plants were identified (fig. S1). In the thin sections, we identified three distinct tube types in the main body of NSC.36. Type 1 tubes are small in diameter (∼10 μm), thin (<1 μm), and smooth-walled, septate with septal pores, sinuous, occasionally branching, and tend to be oriented with the long axis of the specimen (Fig. 2B). Type 1 tubes constitute ∼75% of the body volume. Type 2 tubes are larger in diameter (ranging from 20 to 40 μm), have smooth, double layered (∼2 μm thick) walls, and are aseptate, sinuous, and unbranched (Fig. 2C). Type 2 tubes are similarly oriented with the long axis of the specimen and are intermingled with the type 1 tubes. Type 2 tubes constitute ∼20% of the body volume. Type 3 tubes are the largest in diameter (∼40 μm), thick walled (∼2 μm) with weak annular thickenings (referred to here as banded tubes), and are aseptate and unbranched (Fig. 2D). Type 3 tubes also align with the long axis and comprise ∼5% of the body by volume (Fig. 1, D and H). Despite its size and preservation quality, there was no evidence of reproductive structures, photobionts, or a lichen-style organization in NSC.36. Our new *P. taiti* material adds extensively to the description of *P. taiti*, offering further comparisons to more widespread species. Drawing on reviews of *Prototaxites* species by Chitaley (*10*) and Edwards and Burgess (*15*), we identify similarities in tubes sizes, the presence of banded tubes and medullary spots between our material and other species. Banded tubes are known from *P. hicksii* and *P. storriei* and medullary spots are known from *P. loganii*, *P. crassus*, *P. laxum*, *P. ortoni*, *P. caledonicus*, *P. clevelandensis*, and *P. southworthii*, with both being confirmed as occurring together in *P. storriei* similar to *P. taiti* described here [reviewed in (*1*, *10*, *15*)]. The only characteristic not observed in *P. taiti* or in any of the other species listed above is the presence of growth rings, which are diagnostic only of *P. loganii* (*1*, *10*, *15*). This review demonstrates that species with forms similar to *P. taiti*, especially those with medullary spots, are widespread and have a geological range spanning from the Silurian to the Late Devonian. Therefore, inferences made from *P. taiti* can offer valuable insights into the *Prototaxites* genus as a whole (*10*, *15*)*.*

### Structural differences between *Prototaxites* and Fungi indicate major physiological and developmental differences

The extraordinary quality of preservation in NSC.36 permitted high-resolution imaging of *P. taiti* using CLSM, Airyscan CLSM, and 3D reconstruction. Novel aspects of structure identified in NSC.36 suggest a profound difference in physiology and development between *Prototaxites* and Fungi.

Optical and Airyscan CLSM examination of the medullary spots of NSC.36 revealed these structures to consist of a dense network of the three types of tubes, as well as very fine (<1-μm diameter) tubes (Fig. 2E). High-resolution maps with synchrotron Fourier transform infrared microspectroscopy (micro-FTIR) of the body and the medullary spots revealed that both regions were similar considering the range of the recorded spectra (3000 to 1400 cm^−1^) (figs. S9 to S11). The application of high-resolution imaging and micro-FTIR allowed us to investigate the hypothesis that the body of *P. taiti* was a symbiotic aggregation of two organisms. Both optically and chemically, we observed no differences between tube types. This is consistent with a similar molecular composition, supporting the idea that medullary spots developed through extensive internal branching of the body tubes of a single organism, and that NSC.36 was not a symbiotic association of different organisms. Internal branching was reconstructed using 3D modelling of Airyscan CLSM z-stack data of a region of the tube network. This demonstrates that branching in medullary spots was three dimensional and highly complex on the micron scale, visually comparable to structures involved in gas, nutrient, or water exchange, such as the alveoli of mammalian lungs or blood capillary networks. The closest comparable structures in Fungi in terms of tube size and complexity are the coltricioid hyphae found in the fruiting bodies of some basidiomycetes (*5*), although these are only produced as lateral emergences of wide hyphae that are aligned together (*29*, *30*), and branching order is highly specific, with only generative hyphae producing other hyphal types (*31*). Some ascomycetes exhibit complex hyphal differentiation in the fruiting bodies, but this is of limited relevance to NSC.36, which is nonfertile and the overall dimensions of the tubes of NSC.36 are more similar to those of basidiomycetes than ascomycetes. By contrast to the coltricioid hyphae, medullary spots are found at the nexus of numerous tubes of all tube types, and no branching order can be discerned. This branching complexity, in addition to their occurrence within the body of NSC.36, suggests that medullary spots could have carried out a gas, nutrient, water, or other exchange function.

Type 3 or banded tubes vary in thickness in *P. taiti* (Fig. 2D and fig. S2) and are known from other *Prototaxites* species and related taxa (*15*, *21*, *32*). The occurrence of banded tubes in some *Prototaxites* species (*15*) is contrasted with the rarity of comparable features in extant Fungi. Spiral thickening can be seen in the elaters, thread-like structures with spiral ornamentation, which wrap around spores, of some fungal taxa, but this is rare, being known from the basidiomycete genera *Podaxis* and *Battarrea* (*33*), and their role in spore release suggests a distinct function to the banded tubes of *Prototaxites*, which occur within internal body structures. Another example of spiral thickening in fungal hyphae is illustrated in (*34*), although this is limited to only one end of mycelial hyphae. The annular thickenings seen in banded tubes of *P. taiti* strongly resemble the cell wall thickenings of vascular plant tracheids, which could suggest the convergent evolution of such structures for functions in water conduction and mechanical support. Together, banded tubes and medullary spots call into question fungal affinity of *Prototaxites*. These structures are consistent with functions in transport and exchange of substances without parallels in extant Fungi, such physiological functions could have played a critical role in allowing *Prototaxites* to reach large freestanding sizes.

### The molecular fingerprint of *P. taiti* is distinct from that of contemporaneous Fungi

To further test the fungal affinity of *P. taiti*, we contrasted its molecular composition with that of contemporaneous fossil Fungi in the Rhynie chert using attenuated total reflectance FTIR (ATR-FTIR). Fossil organic matter is produced from selective preservation of original organic material, alteration of this material, and precipitation of newly formed polymers (*35*). For example, during the early phases of diagenesis, resistant polymers may be formed by recombination of sugar and protein compounds [e.g., melanoidin-like material; (*36*)], whereas, in certain conditions, selective preservation of sugar-protein complexes, like chitin-protein complexes, have been reported (*37*, *38*). These processes vary greatly between preservation sites, making comparison of fossils from the same site highly informative. Previous work on the Rhynie chert has demonstrated the application of spectroscopic (*39*, *40*, *41*) and geochemical analyses (*42*–*44*) and therefore permits unique side-by-side comparisons of molecular composition between *P. taiti* and a wide diversity of other taxa, whilst baselining for the influence of diagenesis between similar organisms. Twelve ATR-FTIR acquisition spots were measured across the three *P. taiti* specimens. We hypothesized that if *P. taiti* was a fungus, fossilization products resulting from the selective preservation or diagenetic alteration of glucan sugars would be present, derived from the original main composition of the cell walls including chitin (a polymer of *N*-acetyl glucosamine), or resulting from the recombination of glucan and protein into new melanoidin-like compounds, like those found in Fungi and arthropods in the Rhynie chert (*39*). To test this hypothesis, the previously assembled dataset of 47 samples, representing six higher taxonomic groups (*39*), was combined with 55 additional samples from these six groups and *P. taiti* (see the Supplementary Materials for full spectra for each sample, images of all sample spots, and details of taxonomic assignment). We then developed a novel analytic pipeline comprising two key steps: data exploration (step 1) and modelling (step 2) (fig. S3 and Supplementary Text Extended Methods). Step 1 aimed to show that biologically informative spectral bands correlated with taxonomic classification of specimens and that molecular fingerprints of organisms in the Rhynie chert retained information regarding their biological affinities, and to select the most relevant spectral features for further modelling. In step 2, we built classification models using supervised machine learning approaches to provide a robust statistical framework to accept or reject the taxonomic classification of each sample based on the selected spectral features.

**Fig. 3.**
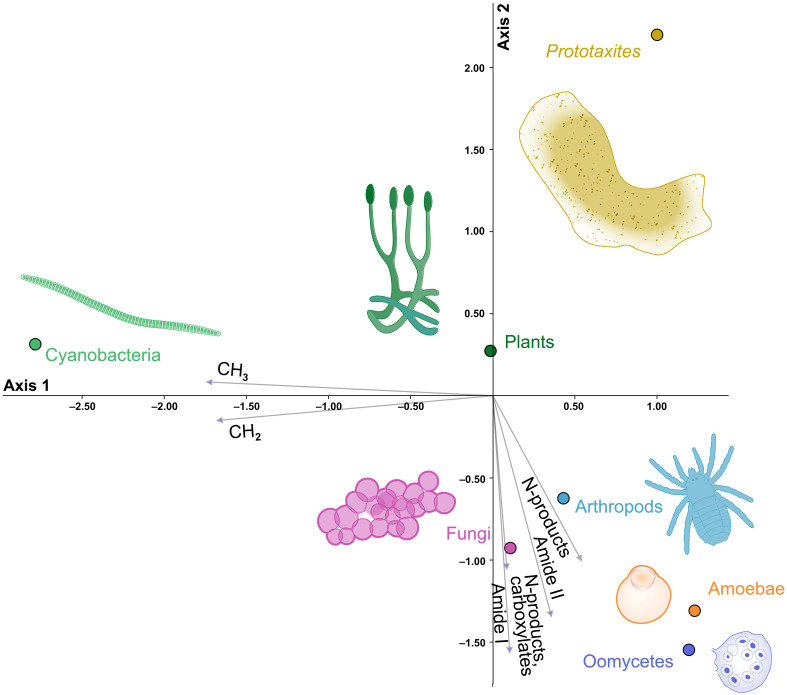
The CCA illustrates the correlation between informative spectral features and the fossils in the Rhynie chert. The CCA uses the most informative ATR-FTIR absorption bands obtained after dimension reduction and feature selection (bands 1, 2, 7, and 10 to 12 in table S1, see Methods, Supplementary Text Extended Methods, and fig. S3). It shows strong correlation between the fossilization products of sugar-protein (amide I, II, N-products, and carboxylate) and Fungi, Arthropod, Oomycetes, and Amoebae. Bacteria show a strong correlation with aliphatic CH*_x_* moieties. On the other hand, *P. taiti*, and land plants, shows a negative correlation with all these products. The CCA supports the differences between the molecular composition of *P. taiti* and Fungi, and differences with the other fossils.

We first performed a canonical correspondence analysis (CCA) on the absorption bands selected using principal components analysis (PCA) (see Supplementary Text Extended Methods). The CCA (Fig. 3) shows good separation between taxonomic groups in the ordination space and demonstrates clear correlations between spectral features and taxonomic assignment. Fungi, arthropods, oomycetes, and amoeba are all strongly positively correlated with bands characteristic of the fossilization products resulting from the alteration of sugar-protein compounds, especially the carbonyl and nitrogen moieties (*45*, *46*), which would also be expected from amino-glucan-rich precursors (*39*, *47*) like chitin. By contrast, bacteria are positively correlated with aliphatic moieties. *P. taiti* and, to a lesser extent, plants are negatively correlated with aliphatic moieties. We conclude that there is a correlation between an organism’s fossilization products and their taxonomic classification, as previously suggested (*39*), and report that the molecular fingerprint of *P. taiti* is distinct from those of chitinous organisms in the Rhynie chert. Having established this correlation, we moved to step 2, where classification models were used to assign samples to a taxonomic group.

Unlike CCA, the classification approach leveraged the full spectra (reduced by PCA) and provided metrics for the confidence of assignment (see Methods and Supplementary Text Extended Methods). We first trained our model on a dataset of *P. taiti* against Fungi, then against all chitinous samples (Fungi and arthropods). Support vector machine (SVM) models were produced for both datasets. All *P. taiti* samples were successfully identified in both test datasets. Globally, the models successfully discriminated *P. taiti*, with a discrimination accuracy of 91% against Fungi and 93% against all chitinous specimens. All other performance metrics (recall, sensitivity, F1; see Supplementary Text Extended Methods) scored above 91% and the Matthews correlation coefficient (MCC), which measures the quality of the binary discrimination, was 0.85 for each dataset, respectively. These results confirm that the fossilization products of *P. taiti* differ strongly from those of Fungi and other chitinous organisms in the Rhynie chert. Having found no support for similarities between *P. taiti* and Fungi, we then tested for similarities between *P. taiti* and bacteria, land plants, and a combined dataset of all other samples. In all cases, our models successfully discriminated *P. taiti* from all other groups (Supplementary Text Extended Methods). Together, these results demonstrate that *P. taiti* cannot be assigned to the Fungi or any other taxonomic group found in the Rhynie chert, and that the fossilization products expected for a fungal composition (*48*), and characteristic of Fungi in the Rhynie chert, are lacking in *P. taiti*.

These findings threw doubt upon the hypothesis that *P. taiti* was a member of the Fungi, prompting deeper molecular investigation. For the classification approach described above, thin-section analysis was the only feasible approach to sample the full diversity of Rhynie chert taxa, some of which are microscopic. One complication of this approach is that, as the samples are embedded in chert, the spectral bands produced by silica can mask portions of the fossil spectra. This masking does not decrease the robustness of our approach, as its influence is identical across samples and silica bands are not present in the features retained for the classification tasks (see Supplementary Text Extended Methods). However, to characterize *P. taiti* in the absence of silica, we analyzed the molecular composition of extracted fossil material (Supplementary Text). This was possible because the original NSC.36 block was large enough to extract organic content solely of *P. taiti* by acid maceration, and also contained a peaty substrate of predominantly plant, but also fungal and arthropod, fossils (see Fig. 1, J and K, and fig. S1). As substrate fossils were too small and interbedded to allow comparison of *P. taiti* with specific taxa, *P. taiti* was contrasted with a bulk extraction of the substrate. The extracted *P. taiti* spectra (fig. S8) included bands characteristic of short/branched aliphatic moieties, carbonyl/carboxyl, aromatic, and ether compounds. We did not observe the ensemble of bands typically associated with polysaccharides (*49*) (C─O─C, CC, and CO stretching in the interval 1200 and 800 cm^−1^), which would be expected with selective preservation of structural sugars, and bands associated with recondensation products of sugars with proteins (*39*, *47*). It is possible that polysaccharides and proteins were lost shortly after the organism’s death or did not form their characteristic resistant C─O/N-rich products (*39*). However, these products were identified in the peaty substrate, indicating that fossilization products of polysaccharides and proteins are preservable in the Rhynie chert.

If *Prototaxites* was a fungus, we predict that, in life, cell walls would have contained chitin and β-glucan complexed with abundant glycoproteins (and possibly subordinate melanin) as in extant Fungi (*48*). *Prototaxites* fossils would therefore, according to this hypothesis, preserve the fossilization products reflecting this composition, in the form of selective preservation, alteration, or recondensation products of these components. Our analyses indicate that fossilization products resulting from sugars and proteins are present in chitinous organisms of the Rhynie chert, such as Fungi and arthropods. However, these fossilization products are not present in *P. taiti.* The simplest interpretation is that, in life, *P. taiti* lacked the typical fungal cell wall composition, including chitin, undermining the interpretation it was a fungus. There is a possibility that the molecular composition of *P. taiti* was uniquely more liable to change or replacement than that of its contemporaneous Fungi or arthropods, despite their occurrence in the same geological setting. Such considerations limit our ability to pinpoint the exact molecular composition of the *P. taiti* cell wall, but bolsters our interpretation that *P. taiti* was unique among the other major groups in the Rhynie chert. We note that melanin is another possible subordinate component of Fungi, but melanin is present across the whole tree of life, reducing its discriminatory power, and is molecularly similar to kerogen (*36*, *47*, *50*), meaning that even if present in the cell wall, its signal would be hidden by the stronger general kerogen signal. We conclude that investigation of melanin in *P. taiti* offers limited evidence for our analysis. Our side-by-side investigation of *P. taiti* and Fungi in the Rhynie chert therefore strengthens our argument that *P. taiti* was molecularly distinct from Fungi. These conclusions are supported by previous investigations of *Prototaxites* spp. from other localities in Canada, England, and Czechia preserved in different taphonomic conditions, although these alternative settings did not allow for the side-by-side comparisons with fossil Fungi achieved in this study (*16*). These previous results suggest that *Prototaxites* was composed of an “extinct polyphenolic structural biomacromolecule” similar to lignin (*16*). The detection of similar fossilization product in our material despite the strong difference in diagenetic history [that is, preservation in chert versus coalified remains in siltstone and sandstone in (*16*)] is interesting and suggests that *Prototaxites* in the Rhynie chert may have been structured by a similar compound. In contrast to the study by Abbott *et al.*, which we find support for here, a study by Vajda *et al.* (*20*) instead suggest a fungal affinity for *Prototaxites* based on spectroscopic analysis. This analysis only concerned aliphatic signatures (stretching of C─H bonds in methyl and methylene groups) and specifically only the ratio methyl/methylene [*R*_3/2_; see (*51*)]. We recognized three areas that require further clarification in their study. First, concerning experimental setup, in the absence of detailed sample preparation information, it cannot be excluded that the organic signal reported resulted from resin fixing the thin section and subsequently captured by the transmission acquisition rather than representing a fossil signature. Second, Materials and Methods indicate that no baseline correction of the spectra was necessary. This is puzzling, as baseline correction is a critical step for spectral preprocessing (*52*) and a correction, consistent between samples, is necessary to obtain peak intensity values that are comparable (i.e., reflecting chemical contributions and not absorption interferences). This calls into question the values obtained for the *R*_3/2_. For example, the asymmetric CH_3_ and CH_2_ absorptions, as presented in the Supplementary Materials of (*20*), show clear differences in spectral shape between the modern fungi and *Prototaxites* that should have been reflected by the ratio values after baseline correction. Third, the authors suggest that this aliphatic ratio can be directly compared to those of living organism, thanks to the low thermal maturity estimated with Raman and the preservation in silica. The thermal maturity of organic material can offer valuable clues about the likelihood of certain compounds being preserved but Raman-based geothermometers and deconvolution methods used to estimate this maturity are often debated, and multiple methods should be applied (*53*). Thermal alteration experiments have showed that microorganisms embedded in silica maintain stable *R*_3/2_ values during heating but that these experimental values never match those observed for natural silica-hosted fossils, suggesting that additional processes played a role in molecular preservation (*54*). Therefore, without comparisons to contemporaneous fossil Fungi from the same locality, it is not possible to confidently determine whether, and which, aliphatic fingerprints of fossil Fungi would be preserved. Together, the results presented in Vajda *et al.* (*20*) require further documentation to be taken as supporting a fungal affinity. However, even if these points are clarified, the results of our current and previous studies (*39*) demonstrate that aliphatic signals alone are not informative in separating fossil Fungi from other eukaryotes, including *Prototaxites*, even when samples are preserved in silica (chert) and have experienced the same diagenetic conditions. Therefore, any studies focused solely on an aliphatic signal are unlikely to be informative for separating fossil Fungi from other organisms with different diagenetic history. In conclusion from our molecular composition analysis, we conclude that *Prototaxites* had a conserved and distinct molecular fingerprint from contemporaneous Fungi, and our results are in line with previous results reported by Abbott *et al.* (*16*).

### The biomarker perylene was not detected in *P. taiti*

Last, to further interrogate interpretation of *P. taiti* as a fungus and specifically as an ascomycete (*6*), we performed a biomarker analysis to test for the presence of perylene. Perylene is a known biomarker for phytopathogenic Fungi in the Rhynie chert (*43*) and is derived from perylenequinones, pigment compounds produced predominantly by ascomycete Fungi (*55*). We contrasted samples consisting exclusively of *P. taiti* with those of the peaty substrate. Perylene was not detected in *P. taiti*, but was detected in the substrate (fig. S4), likely from the presence of ascomycetes (Supplementary Text). This biomarker analysis does not support an ascomycete affinity for *P. taiti*, and underlines the distinctness of *P. taiti* from other organisms in the Rhynie chert.

### No evidence for fungal affinity of *Prototaxites*

**Fig. 4.**
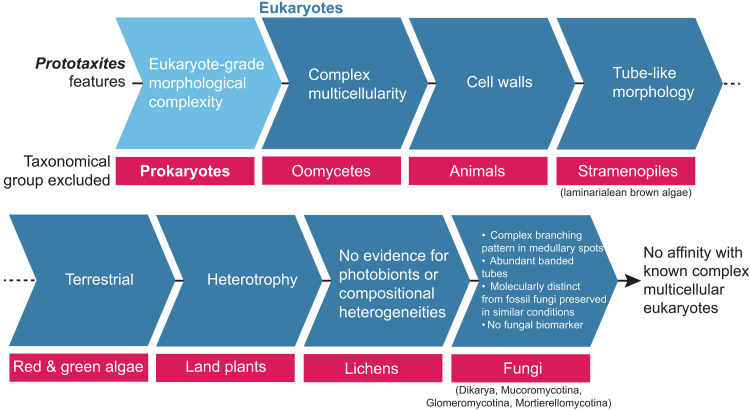
The morphology, metabolism, ecology, and chemistry of *Prototaxites* preclude its placement in known crown lineages of multicellular eukaryotes.

Complex multicellularity [i.e., organisms with cell-cell adhesion, inter-cellular communication and tissue differentiation; see (*56*)] is known only in three main eukaryotic lineages: Archeoplastids, in red algae, green algae, and Land plants; Stramenopiles, in laminarialean brown algae; and Opisthokonts, in animals and fungi (*56*). Previous investigation showed that *Prototaxites* was a eukaryotic terrestrial heterotroph made of tubes with cell walls (*7*), which exclude a prokaryotic, archeoplastidal, animal, or laminarial affinity (Fig. 4). Within the Fungi, four lineages are known to build complex multicellular structures: Mortierellomycotina, Glomeromycotina, Mucoromycotina, and Dikarya (Ascomycota and Basidiomycota) (*57*). In addition to discrepancies in evolutionary timing (*7*), *Prototaxites* differ anatomically and chemically from these lineages and the multicellular structure they produce, notably in their patterns of tube branching, the presence of abundant banded tubes, and in their fossilization products, which are inconsistent with both a fungal molecular composition and with contemporaneous Fungi preserved under similar taphonomic conditions.

We hypothesized that if *P. taiti* were a fungus, it would have shared, in life, a molecular composition comparable to that of contemporaneous crown-group fungi preserved alongside it in the Rhynie chert. All extant Fungal clades, including the unresolved basal taxa Rozellidae (*58*), have various amounts of chitin (or chitosan) during at least part of their life cycle, as well as β-glucan and abundant glycoproteins (*48*, *59*). The secondary loss and replacement of these foundational cell wall components would require a major alteration of main developmental pathways, especially in higher lineages (such as Dikarya). Our molecular data therefore do not support the placement of *Prototaxites* within Basidiomycota or Ascomycota, even within a stem group. For the same reason, it is also unlikely that *Prototaxites* is the result of an increase in complexity within an early-diverging fungal branch, e.g., Mucoromycota. Furthermore, our optical and molecular fingerprint analyses provide no evidence that *Prototaxites* was a symbiosis between multiple species: All tube types were found to interconnect at the spots, shared similar anatomical and chemical characteristics, and exhibited no evidence of photobionts. The documentation of these features in a *Prototaxites* specimen from the Rhynie chert is particularly important, as this ecosystem preserves clear evidence of lichen, cyanobacterial, and plant-fungal symbioses, making it an ideal site to test for such complex interactions.

A previous interpretation of *P. taiti* suggested that it was a “basal ascomycete” (*6*), a finding that bolstered the overall hypothesis that *Prototaxites* was a Fungus. However, our new data challenge this interpretation. First, reinvestigation of the type material highlights the absence of connection between fragments assigned to Ascomycota and material diagnostic of *P. taiti*. Using new *P. taiti* specimens, we demonstrate that *P. taiti* is structurally distinct from all known Fungi, that the molecular fingerprint of *P. taiti* is distinct from contemporaneous fossil Fungi in the Rhynie chert, and that *P. taiti* lacks the fungal biomarker perylene. With no support for a Fungal affinity, we suggest that *Prototaxites* is best considered a member of a previously undescribed, independent and extinct lineage of complex multicellular eukaryotes (Fig. 4). Our results also directly support previous investigations of the molecular composition of *Prototaxites*, suggesting a possible structural composition of polyphenolic compounds (*16*). These results coupled with the banded structure of larger tubes and the intricate branching pattern within the medullary spots, constitute a major advance in understanding the biology of *Prototaxites*.

## MATERIALS AND METHODS

### Rhynie chert specimens

NSC.36 was originally collected in farmland owned by the Windyfield Farm (NJ 349642 mE, 827852 mN) adjacent to the site of special scientific interest by a local landowner before 2021. Specimens were passed to North Sea Core to help distribute the samples for academic research with the mutual agreement of NatureScot. Blocks were distributed using the accession numbers North Sea Core NSC.01-NSC.45. NSC.36 was processed in the lab of A.J.H. and the remaining subblocks of NSC.36 (Fig. 1K) are deposited in National Museums Scotland, UK (G.2024.5.1 and G.2024.5.2). Alongside our investigation of NSC.36, we also carried out a reexamination of the four thin sections that constitute the type material of *P. taiti* in The Hunterian, University of Glasgow (GLAHM Kid 2523–2526) as well as blocks of Rhynie chert containing *P. taiti* in the Lyon Collection, University of Aberdeen, UK (Lyon 156 and Lyon 48).

### Sample preparation

NSC.36 was first cut in half (Fig. 1J) and a subblock containing *P. taiti* was produced (Fig. 1K), from which six thin sections were made; three uncovered 100-μm-thick specimens for FTIR [accessioned into National Museum Scotland (NMS) G.2024.5.3, G.2024.5.4, and G.2024.5.5] and three 30-μm-thick thin sections with coverslips for light and confocal microscopy (initially named MPEG0056, MPEG0057, and MPEG0058, accessioned as NMS G.2024.5.6, G.2024.5.7, and G.2024.5.8, respectively), the first two sections were taken from faces of the subblock perpendicular to each other to allow for the anatomy to be studied from different orientations. New covered and uncovered thin sections were made from blocks 156 and 48 containing *P. taiti* from the Lyon Collection, University of Aberdeen, UK. A total of four uncovered (UC) thin sections were created for FTIR analysis: Lyon 156 UC1, Lyon 156 UC2, Lyon 156 UCB1, and Lyon 48 UC1. A total of five thin sections with coverslips were made for light and confocal microscopy (Lyon 156 MPEG0071, Lyon 156 MPEG0072, Lyon 156 MPEG0073, Lyon 156 MPEG0078, and Lyon 48 MPEG0070). Covered and uncovered thin sections of NSC36 were deposited in National Museums Scotland, UK. All new thin sections created from material from the Lyon collection were returned to the Lyon Collection, University of Aberdeen, UK.

### Photogrammetry

For all three of the photogrammetry models [Fig. 1D and fig. S2A (Lyon 156), Fig. 1I (NSC.36 before cutting), and Fig. 1J (NSC.36 after cutting)] a Canon EOS 5D Mark IV camera with a 100-mm macrolens and tripod was used, with the following settings: ISO 100, f1/16, and 1/5 s. An adapted version of the photogrammetry protocol of (*60*) was used, in which the block was placed on an automated turntable (Genie Mini II and turntable) within a light box (Neewer) for even illumination. Each block had a series of 16 photos taken through a full rotation of the turntable, with the camera moved through four heights of the tripod (photos taken from ~0°, ~30°, ~50°, and ~70° relative to the plane of the turntable) and a full rotation series of 16 photos taken at each of these heights. This full procedure (four heights, 16 photos at each height) was conducted twice for each block, resting first on one face then flipped to rest on the converse face (2.5 + 2.5 photogrammetry method). Scaled photogrammetry models were created using AgiSoft PhotoScan Professional and exported to CloudCompare to produce scaled renders.

### Optical microscopy

Thin sections were studied and photographed using a Nikon ECLIPSE LV100D compound microscope (Figs. 1L and 2, A, E, H, and I, and fig. S2D) and a Nikon SMZ18 Stereoscope (Fig. 1E and fig. S2C). Figure 2E was taken using extended depth of focus (EDF Capture) in the NIS Elements software.

### Confocal microscopy

A Zeiss LSM 880 confocal microscope with Airyscan was used to produce regular and Airyscan CLSM images. Single plane Airyscan images (Fig. 2, C and D, and fig. S2, E and F) were taking using a ×40 oil immersion lens, 488- and 561-nm lasers, longpass emission window, 2.5 Au pinhole, pixel size 0.05 μm, and 2576 × 2576 frame size. Regular CLSM settings (with all other parameters the same as those used for Airyscan with the exception of a 1 Au pinhole) with image stitching was used to create the image in Fig. 2B. Z-stack images were produced with all settings as for single plane Airyscan CLSM imaging, with a 0.27-μm interval between successive images, corresponding to a total of 114 slices.

### 3D reconstruction

The Airyscan CLSM z-stack of the medullary spot region was first opened in Fiji (*61*), subjected to image contrast enhanced on all slices, color inverted, then saved as a jpeg file series. 3D reconstruction was conducted using SPIERS (*62*). The jpeg file series was imported to SPIERSedit, and a single large axis was picked to reconstruct branching (axis in top right of Fig. 2F). After application of automatic thresholding to the image series, the branching pattern was traced through the image series and segmented using curves, the thresholded image was then adjusted manually, and masks were produced from curves (illustrated in cyan on Fig. 2, F and G). Images were rendered in Blender, with adjustments to light, shading and color for clarity of the resulting render (Fig. 2G).

### Transmission benchtop FTIR

Small fragments of the NSC.36 block containing only *Prototaxites* were macerated in acid to remove silica following a low-manipulation protocol adapted from (*63*), in the Oceanography Clean Laboratory (School of Geosciences, University of Edinburgh). The samples were placed in a 120-ml Teflon jar and bathed overnight in hydrochloric acid (HCl) to remove potential carbonate influence (no reaction was noted). HCl was discarded, and the samples were left for a week in analytical grade hydrofluoric acid with daily light hand-agitation of the jar. After complete dissolution and sedimentation, the acid surplus was removed, and samples were brought to boil with HCl for half a day to remove or prevent the formation of secondary minerals. The acid was then neutralized by successive washes with water. A drop of macerate was deposited on a zinc selenide window, placed to dry in an oven at 60°C for 2 hours, and a spectrum was acquired in the range 4000 to 650 cm^−1^, with a spectral resolution of 4 cm^−1^ and 16 accumulations, using an AlphaII FTIR spectrometer (Bruker) at the UK Centre for Astrobiology (School of Physics and Astronomy, University of Edinburgh). Spectra were preprocessed using Quasar 1.9.2 [Orange (*64*, *65*)] (atmosphere corrected, Gaussian smoothing, and baseline correction). A second derivative spectrum was obtained by applying a SavitzkyGolay filter of order 2 and window 21. Band assignments are shown in table S1.

### Attenuated total reflectance-FTIR

ATR-FTIR was conducted at room temperature on a Smiths IlluminateIR microscope equipped with a liquid nitrogen–cooled MCT detector and a diamond-coated ATR objective (magnification ×36) at the School of Chemistry (University of Edinburgh). Backgrounds were taken in air before analysis. We acquired the spectra in reflection mode by combining 128 accumulations in the range 4000 to 650 cm^−1^ at a resolution of 4 cm^−1^ with the software Qual ID 2.51 (Smiths). We processed the spectra using software Quasar. We truncated the spectra to analyze the range between ca. 1445 to 1750 cm^−1^ and 2760 to 3000 cm^−1^, removing intervals due to hydroxyl absorptions (4000 to 3000 cm^−1^), intense vibration of silica (1400 to 650 cm^−1^), atmospheric CO_2_, ATR diamond absorption, and high–wave number silica overtones (2760 to 1750 cm^−1^). We corrected the baseline, applied a light Gaussian smoothing. Last, to minimize the influence of thickness and differences in concentration of organic matter, the spectra were normalized on the highest silica overtone absorption peak (1615 cm^−1^). For inspection and band assignment, average second derivative spectra for each domain (chitinous and nonchitinous) were obtained by applying a Savitzky-Golay filter (figs. S7 and S8). Band assignments are shown in table S1. Additional spectra of Rhynie chert samples of plants, fungi, animals, peronosporomycetes (oomycetes), and amoeba were taken from (*39*).

### Data exploration—Features selection

After preprocessing, the spectra were organized into four datasets: (1) Prototaxites versus bacteria, (2) Prototaxites versus Fungi, (3) Prototaxites versus chitinous organisms (Fungi + arthropods), and (4) Prototaxites versus plants (including plant spores). A fifth dataset comprising the 102 samples from plants (*n* = 37), Fungi (*n* = 24), arthropods (*n* = 12), bacteria (cyanobacteria; *n* = 10), Peronosporomycetes (oomycetes; *n* = 4), Amoebae (*n* = 3), and Prototaxites (*n* = 12) is also compiled for one-class classification (see below). For datasets 1 to 4, each sample was one-hot encoded according to their biological group. These formatted datasets were then processed through the pipeline illustrated in fig. S3. Analyses were carried out in Python 3.12.8. Data exploration began by exploring the data structure with PCA. PCA is a fundamental step before performing supervised classification tasks because it performs a reliable dimension-reduction that allows selection of robust features for classification while remaining easily interpretable. PCA was conducted using the scikit-learn package. We extracted the first ten principal components (PCs) with their respective explained variance and cumulative variance. To control the robustness of our PCA, we compiled scree plots, scores and loading spectra, and tested the stability of each PCA by calculating the cosine angle between the original dataset and resampled dataset using bootstrapping (100 bootstraps). A cosine angle close to 1 for a PC indicates low variation and shows that the PCA is not sensitive to variation in the dataset for this PC (i.e., this PC represents a real pattern in the data) (*66*). Last, using the most stables PCs as references, we performed outlier detection using Hotelling’s T2 versus Q residual values (*52*, *67*). Detected outliers were removed, and each dataset was recompiled. After these first robustness checks, we performed new PCA analyses on the recompiled datasets. On the basis of the robustness checks for these new analyses, we selected PCs for further supervised learning that retained sufficient variance and represented interpretable biological information (see Supplementary Text Extended Methods). The retained PCs (PC1 and PC2 for dataset 1 and PC1-PC4 for datasets 2 to 4) were directly used as variable for classification tasks (see below). Last, we extracted the intensities of the main biologically informative absorption bands in the spectra, based on the loading spectra of the retained PCs. These bands (bands 1, 2, 7, and 10 to 12 in table S1) were used for correlation analysis with CCA and for further classification tasks as a comparison with the full spectra approach described above.

### Data exploration—CCA

CCA is a multivariate supervised statistical method used to explore the correlation between a matrix of response variables and a set of explanatory variables (*68*, *69*). CCA was conducted on the extracted bands using the R package Vegan with scaling parameter equal to 1 (*70*) called via Python using rpy2 (*71*) with the Standard Scaler from scikit-learn (*72*) used to preprocess the bands before conducting the CCA. We used our lineages as response variables (with each sample one-hot encoded) and the extracted band intensities as explanatory variables. Results of the ordination and correlation between the lineages and the spectral feature can be seen in Fig. 3.

### Modelling

Multiclass supervised classification tasks were performed according to the pipeline presented in fig. S3. Modelling was performed in Python 3.12.8 using the scikit-learn package on the datasets without outliers. To keep the models simple, we performed binary classifications. Similarly, we follow a parsimony principle by testing first simple linear models [linear discriminant analysis (LDA)] before moving to a more complex algorithm (SVM) (*52*). Data were randomly split into training (70%) and test sets (30%), while retaining the ratio of each lineage (stratified). After splitting, the training data were subjected to dimension reduction using PCA retaining a number of PCs determined by data exploration steps. The training dataset for datasets 2 to 4 show a strong data imbalance between each class. To address this imbalance, we performed synthetic minority oversampling technique (SMOTE), which generates synthetic examples for the minority class (*73*). PCA and SMOTE are performed after data splitting to avoid data leakage, which would compromise the test set and the robustness of the models. For all analyses, validation was perform using leave-one-out cross-validation on the training set, a method well adapted for small datasets (*52*, *74*). After cross-validation, the model was run on the whole training set. For each analysis, learning curves were computed to control if the size of our datasets were sufficient to obtain robust results. For each LDA, we computed score plots and controlled robustness using bootstrap stability tests, confusion matrix and five performance metrics (accuracy, precision, recall, F1, and MCC). Each SVM analysis followed the same robustness checks. For SVM we also computed the two-dimensional decision boundary based on the best parameters (C and gamma) obtained after grid-search during cross-validation. LDA performed well for dataset 1, scoring 1 for each parameter in both the training and the test set. Other datasets showed improved performance and robustness with SVM, consistently scoring between 0.91 and 0.96 for each of the metrics and above 0.8 for MCC in both training and test sets (see details in Supplementary Text Extended Methods). Unlike full spectra, models using selected individual bands performed poorly, showing a strong discrepancy between training and test set performances, especially lower MCC. Last, one-class modelling was used to show that *Prototaxites* is not only molecularly different from the Rhynie chert bacteria, plants, and chitinous organisms when treated separately, but also from all Rhynie chert fossils taken together (dataset 5). We performed one-class modelling using data-driven class analogy (DD-SIMCA) as developed by Kucheryavskiy *et al.* (*75*) (www.mda.tools/ddsimca). We trained a PCA model on the Prototaxites samples (*n* = 12). To mitigate the effect of the small sample size, we use resampling leave-one-out cross-validation. We used a rigorous feature selection approach based on sensitivity, and less permissive outlier detection using robust parameter estimates on the training set (*75*, *76*). No outliers were detected. We tested all non-*Prototaxites* fossils with the model trained on *Prototaxites* samples. The model provided an excellent true negative rate (specificity) of 0.911, indicating high confidence that *Prototaxites* differs from all other Rhynie chert fossils in its fossilization products.

### Synchrotron FTIR

A double polished thin section (80 μm) was produced from *Prototaxites* NSC.36 (School of Geosciences, University of Edinburgh) and analyzed at the B22 beamline of Diamond Synchrotron (UK). Two maps were acquired through a medullary spot and the body, and one line was acquired through a type 2 tube. Using Quasar, spectra were atmosphere corrected and truncated between 3000 and 1440 cm^−1^. Second derivatives were calculated using a SavitzkyGolay Filter of order 2 and window 9. We applied a light Gaussian smoothing and a vector normalization.

A total of 975 individual spectra were extracted from the maps and line, and labeled as body (i.e., dense pack of type 1 and 2 tubes) and medullary spot (mix of dense tiny tube and branching larger tubes). We did not find informative difference between the two groups (see fig. S11). The preprocessing of the spectra was repeated using the steps above, but with baseline correction instead of calculation of the second derivative. CH_2_ asymmetric stretching bands at ca. 2925 cm^−1^ were integrated together with CH_3_ asymmetric stretching bands at ca. 2960 cm^−1^, and a CH_3_/CH_2_ semiquantitative ratio was calculated on the basis of band intensity. This ratio provides information about carbon chain length and branching; the lower the ratio, the higher the chain length and the more limited the branching (based on the construction of alkane chain with CH_3_ at the extremity and CH_2_ forming the chain, e.g., (*39*, *47*, *51*, *54*, *77*–*79*). We calculated average ratio values of 0.75 for the body and 0.71 for the medullary spots, which both correspond to chain length of ca. nine carbons (*51*).

### Biomarkers

For biomarker analysis, samples of block NSC.36 were separated into those consisting of bulk material absent *P. taiti* and those consisting entirely of *P. taiti*, to enable comparative analysis. A standard clay brick was used as a control material (furnaced at 550°C for 12 hours and underwent every step of the sample preparation, extraction, and analysis that the NSC.36 samples were subjected to). All glassware, ceramic ware, and aluminium foil were furnaced at 550°C for 12 hours. Procedural controls containing no material were run alongside every NSC.36 and combusted brick sample from extraction to analysis, to control for contamination introduced in the laboratory. Samples were prepared for extraction in the Earth Surface Research Laboratory at Trinity College Dublin. Large pieces were first broken down to smaller pieces in a jaw crusher and subsequently milled to a powder using a ball mill. Components of the jaw crusher and milling equipment that came into contact with the sample material were cleaned with deionized water, methanol, and chloroform between uses. Smaller fragments of *P. taiti* were crushed to a powder using a mortar and pestle.

Samples were weighed into 50-ml glass centrifuge tubes with polytetrafluoroethylene lids (ca. 13 g for bulk and brick and ca. 3 g for *Prototaxites*). One hundred microliters of 100 parts per million (ppm) squalane was added to the combusted brick and procedural control to estimate % recovery. Samples and controls were ultrasonically extracted three times with 20 ml of 9:1 chloroform:methanol at 40°C for 15 min. Following each extraction, tubes were centrifuged at 3000 rpm for 30 min. Extracts were transferred to round bottom flasks with a glass Pasteur pipette. Combined extracts were then filtered through glass fiber filters (Whatman GF/A) before being concentrated under anhydrous N_2_. Concentrated extracts were transferred to 2-ml gas chromatography (GC) vials, dried under anhydrous N_2_, and subsequently resuspended in 100-μl extraction solvent. One hundred microliters of 100 ppm 5α-cholestane was added to the combusted brick and procedural control as an internal standard. Samples were extracted in triplicate by ultrasonication with 9:1 (v/v) chloroform:methanol at 40°C. Total lipid extracts were combined and concentrated under anhydrous N_2_ before analysis. Aliquots (1 μl) of samples were injected in triplicate onto an Agilent model 7890 N gas chromatograph coupled to an Agilent 5973 N mass selective detector operating in electron impact mode at 70 eV. The column was a 30-m HP-5MS column (0.25-mm inner diameter and 1-μm film thickness). Each sample was injected with a 2:1 split ratio. The GC inlet temperature was 280°C and the oven program was 65° (held 2 min) to 300°C (held 20 min) at 6°C/min. Each sample was run in full scan and selected ion monitoring modes (mass-charge ratio of 252 for perylene). Individual compounds were assigned for comparison with mass spectral library databases (NIST98 and Wiley275) and comparison of mass spectrometry patterns with published spectra.

## Supplementary Materials

### The PDF file includes:

Supplementary Text Figs. S1 to S33 Table S1 Legends for data S1 and S2 References
