# Supplementary material for: Prototaxites fossils are structurally and chemically distinct from extinct and extant Fungi
Source: Sci Adv. 2026 Jan 21;12(4):eaec6277. doi: 10.1126/sciadv.aec6277 (PMC12822639; doi:10.1126/sciadv.aec6277)
Supplement: Supplementary file 1 — Supplementary Text Figs. S1 to S33 Table S1 Legends for data S1 and S2 References [file sciadv.aec6277_sm.pdf]

Science Advances
AAAS

Supplementary Materials for

Prototaxites fossils are structurally and chemically distinct from extinct and extant Fungi

Corentin C. Loron *et al.*

Corresponding authors: Corentin C. Loron, corentin.loron@ed.ac.uk;
Alexander J. Hetherington, sandy.hetherington@ed.ac.uk

Sci. Adv. **12**, eaec6277 (2026)
DOI: 10.1126/sciadv.aec6277

The PDF file includes:

Supplementary Text
Figs. S1 to S33
Table S1
Legends for data S1 and S2
References

Other Supplementary Material for this manuscript includes the following:

Data S1 and S2

Supplementary Text

Provenance of Material

The block NSC.36 was originally collected in farmland owned by the Windyfield Farm (NJ 349642 mE, 827852 mN) adjacent to the Rhynie chert Site of Special Scientific Interest (SSSI) by a local landowner before 2021. Specimens were passed to North Sea Core to help distribute the samples for academic research with the mutual agreement of NatureScot. Blocks were distributed using the accession numbers North Sea Core NSC.01-NSC.45. NSC.36 was processed in the lab of AJH and the remaining sub-blocks of NSC.36 (Fig. 1K) are deposited in National Museums Scotland, UK (G.2024.5.1 and G.2024.5.2). The NSC.36 block was validated for authenticity as originating from the Rhynie chert deposit by AJH, and dating by association with the age estimates of the Rhynie chert deposit.

Way up orientation of NSC.36

Although the Rhynie block NSC.36 lacks a recorded orientation, the way-up direction can be inferred from the geopetal features illustrated in **Fig S1**: (1) truncated laminae; (2) compaction localised laterally to one side of the *Prototaxites* specimen, inferred to be the underside; (3) relatively uncompacted plant axes in the crook of the boomerang-shaped *Prototaxites*, part of a probable geopetal sediment fill that thins out laterally; (4) a similar geopetal infill on one side of the *Prototaxites*.

Qualitative analysis of extracted *Prototaxites* and Rhynie chert bulk FTIR spectra

The spectrum displays strong absorption bands attributed to stretching and deformation of CH bonds, C=O, CO/CC, OH and aromatic moieties (see **Figure S7** and **Table S1**). *Prototaxites* is characterised by a strong absorption at 1710 cm^{-1} (characteristic of carboxyl and conjugated ketone[1]) associated with strong contribution of aromatic rings at 1595 cm^{-1} and C-H_x deformation at 1460 cm^{-1}. Additional absorption can be observed at 1120 cm^{-1} attributed to aromatic C-H in plane deformation and C-O-C and C-O/C-C stretching as well as 1370 and 1270

cm^{-1}, attributed to O-H and C-O stretching in phenols (e.g.,*79-81*). In the 1200-800 cm^{-1} interval, we noticed the absence of the three-peaks absorption associated with carbohydrates (*44*), suggesting the peaks at 1040 and 1120 cm^{-1} do not result from stretching in preserved polysaccharide products (*47*). The absence of the ensemble of bands at 1650 and 1540-1575 cm^{-1} additionally indicates that no fossilisation products of the alteration such polysaccharides, or from the recondensation of sugar and protein material (melanoidin-like material (*82*)) are present.

The bulk material from the surrounding peaty chert substrate (containing plants as well as other organisms such as fungi, and fragments of arthropods possibly fungi, but no *Prototaxites*) also shows absorption of carboxyl and conjugated ketone at 1715 cm^{-1}, aromatic rings at 1605 cm^{-1}, C-H_x deformation at 1458 cm^{-1} and bands of C-O-C, C-O and C-C. Bands for phenol are present at 1260 cm^{-1} and 1335 cm^{-1}. In addition, bands characteristic of recondensation products of sugar-protein are clearly present at 1650 and 1535 cm^{-1}.

Our results are in accordance with a previous FTIR study of other *Prototaxites* specimens from Canada, England and Czechia (*16*) that detected a polyphenolic structural macromolecular composition for these specimens.

<u>Biomarker supplementary results</u>

Lipid biomarker analysis supports a possible non-ascomycete origin for organic matter (OM) within the *P. taiti* specimen. Both bulk peaty substrate and fragments of the pure *P. taiti* body were extracted using an ultrasonic assisted extraction (UAE) method and analysed by gas chromatography – mass spectrometry (GC-MS). We applied several organic geochemistry proxies to the resulting data including the carbon preference index (CPI) which is a measure of the odd-over-even predominance of long chain *n*-alkanes (Eq. 1 in (*83*), and the pristane/phytane (Pr/Ph) ratio which compares the abundance of two compounds derived from the phytol side chain of chlorophyll (*84*).

(1) $CPI_{25\text{-}33} = 0.5 \times [(C_{25}+C_{27}+C_{29}+C_{31}+C_{33})/(C_{24}+C_{26}+C_{28}+C_{30}+C_{32})] + [(C_{25}+C_{27}+C_{29}+C_{31}+C_{33})/(C_{26}+C_{28}+C_{30}+C_{32}+C_{34})]$

CPI values of 1.07 and 1.26 for the bulk material and *P. taiti*, respectively, suggest a terrestrial OM source of high thermal maturity. Pr/Ph ratios for both samples indicate a sub-oxic to oxic environment, with potentially more oxic conditions reflected in the bulk material (Pr/Ph 2.79) than the *P. taiti* specimen (Pr/Ph 1.69). These values are similar to those reported by Akinsanpe et al. (*85*) in their biomarker analysis of the Rhynie chert. It has been suggested that the polycylic aromatic hydrocarbon perylene in geological materials may be derived from perylenequinones, natural pigments produced by some species of Ascomycota, and therefore acts as a biomarker for (ascomycete) fungi in the fossil record (*55, 86*). Perylene was identified in the substrate material but not in the *P. taiti* sample via SIM analysis for m/z 252. Ascomycete remains present in the substrate material may be the source of perylene in this instance, however it should be noted that there are other potential sources for this compound, such as plants and animals, though in both cases this is a minor source (*43*). If *P. taiti* were a large ascomycete that produced perylenequinones, it would be expected that the perylene signal in the organic matter derived from the *P. taiti* sample would be stronger than that of the substrate material, however it was not detected. This suggests *P. taiti* was not an ascomycete. Alternatively, if perylene in the substrate was produced by plants and animals, its absence from the *P. taiti* specimen adds further support to the widely held view that it cannot be assigned to either of these groups. Taken together, the absence of perylene in *P. taiti* therefore further demonstrates the chemical uniqueness of *P. taiti* compared to other eukaryotes in the Rhynie chert.

<u>Extended methods: Detailed extended methods and justification of workflow for the molecular composition analysis.</u>

Contents

1. Data Exploration 6
   1.1. Starting hypotheses and dataset selection 6
      1.1.1. Hypotheses 6
      1.1.2. Datasets 6
   1.2. Principal Component Analysis (PCA) 8
   1.3. Robustness analyses and feature selection 8
      1.3.1 Robustness analysis 8
      1.3.2. Feature selection for classification 11
   1.4. Canonical Correspondence Analysis (CCA) 12
2. Modelling (Classification) 13
   2.1. Multi-class classification. 15

2.1.1. Parsimony principle 15
2.1.2. Imbalanced dataset 15
2.1.3. Model evaluation 15
2.1.4. Whole spectra analyses (PC features) 19
2.2. One-class modelling 22
3. Conclusion 24

To define the original molecular composition of *Prototaxites* and complement our morphological reinvestigation, we investigated its *in situ* molecular fossil fingerprint using infrared spectroscopy. Spectroscopic signatures of fossils can be challenging to interpret due to the influence of diagenesis, producing signal convergence with increased maturation (*87*), in addition to convergence due to similar original molecular precursors (e.g., different sugars), and overlapping mineral absorption. Because of this inherent complexity, the sources of confounding variables must be reduced, and quantitative tools must be used to help understand patterns in data. To do this in our analysis, we compared the molecular fingerprint of *Prototaxites* obtained with Attenuated Reflectance Fourier Transform Infrared Spectroscopy (ATR-FTIR) to those of other organisms in the Rhynie chert. This was done using a full spectrum approach and without prior selection of features of interests.

This approach relies upon a taphonomic assumption: that in the Rhynie chert, all organisms were exposed to broadly similar diagenetic environmental conditions (e.g., the same temperature and pressure), baselining the effect of differential molecular degradation between organisms. The resulting signals will therefore account for their respective original molecular composition modified under similar conditions, minimising the confounding effects of diagenesis. Our approach is also data-driven to minimise arbitrary choices in spectral feature selection, making it an agnostic approach.

This extended method aims to explain our analysis framework and is separated into two parts. Part 1 is focused on data exploration, and Part 2 uses modelling for classification.

1. Data Exploration

The first part of the method concerns data exploration. It aims to investigate the patterns in the data and select features for classification which reflect biological information in the samples. To achieve these, we set out four main hypotheses and used distinct datasets of our FTIR samples:

1.1. Starting hypotheses and dataset selection

1.1.1. Hypotheses

(H1) The molecular fingerprints of organisms in the Rhynie chert retained information regarding their biological affinity.

(H2) The molecular fingerprint of *Prototaxites* differs from the molecular fingerprints of fungi and other chitinous organisms in the Rhynie chert.

(H3) The molecular fingerprint of *Prototaxites* differs from the molecular fingerprints of cyanobacteria and plants in the Rhynie chert.

(H4) The molecular fingerprint of *Prototaxites* is unique in the Rhynie chert.

1.1.2. Datasets

To test each of these hypotheses, we built the following 5 datasets.

(D1) The full dataset of 102 samples from Plants (n=37), Fungi (n=24), Arthropods (n=12), Bacteria (= cyanobacteria; n=10), Peronosporomycetes (oomycetes, n=4), Amoebae (n=3), and *Prototaxites* (n=12).

(D2) *Prototaxites* vs Bacteria

(D3) *Prototaxites* vs Fungi

(D4) *Prototaxites* vs Chitinous organisms (Fungi + Arthropods)

(D5) *Prototaxites* vs Plants

Having detailed our hypotheses and the datasets, we then conducted three successive steps: Principal Component Analysis (PCA) for dimension reduction (Section 1.2), robustness analyses and feature selection (Section 1.3), and Canonical Correspondence Analysis (CCA) (Section 1.4). The reasoning for doing each of these successive steps is that in concert they are vital for generating robust results. The importance of dimension reduction cannot be overstated so, before

outlining these steps, we will demonstrate why dimension reduction is necessary using the full dataset (D1).

Dimension reduction is a necessary and fundamental step before application of supervised classification methods, as these methods are sensitive to high dimensionality (the "curse of dimensionality"). The goal of dimension reduction is to remove multicollinearity between variables - which otherwise undermines the reliability and interpretability of the model - but also to eliminate noise and redundancy, reducing the risk of overfitting and improving model generalisation.

Using the full spectra to perform classification analyses results in strong overfitting. For example, if we take our full dataset (D1) described above and carry out a Linear Discriminant Analysis (LDA), the discriminant space projection shows excellent separation between classes (**Fig. S12**). This may incorrectly be taken as support for these organisms producing very distinct molecular fingerprints, and therefore originally possessing very distinct molecular compositions. However, this approach is misleading, as each discriminant component's loading spectra (the plot showing which variable contributes the most to the ordination) do not correspond to informative biological bands (**Fig. S13**). This result demonstrates that we must ensure the analysis is only conducted on informative biological bands. To do this, the complexity of the model (e.g., the number of classes analysed), the correlations between variables and the high number of dimensions must all be reduced. Moreover, the model must be validated by a separate unseen test set to demonstrate its generalisation performance (*52*). Taken together, this requires us to first carry out a process of dimension reduction on our datasets before commencing further analysis.

We can demonstrate the impact of dimension reduction by repeating the analysis of dataset D1 outlined above but with the inclusion of a dimension reduction step using Principal Component Analysis (PCA). This produces overlap between the classes in the two-dimensional biplot (**Fig. S14**), but their distribution in space is now based on features reflecting biological information (PCs 1 and 2, reflecting here the contribution of aliphatic and sugar-protein fossilisation products, respectively; **Fig. S15**). Overlap in this two-dimensional space results from the convergence between spectroscopic signals and the simplicity of the model, which uses simple linear correlations in a reduced two-dimensional space. However, this does not imply that the classes cannot be discriminated in higher dimensions, and with more appropriate models.

For all our analyses of datasets D1-D5, we carried out an initial dimension reduction step using PCA. Our choice of the number of dimensions retained was based on the robustness analyses and feature selection processes detailed below.

1.2.Principal Component Analysis (PCA)

PCA is a quick and reliable dimension reduction method, and so is a common data exploration and feature extraction approach to spectral data. It is important to note that PCA is not a classification method, so is not used here for classification (classification was done separately, in part 2 of our analysis pipeline). Rather, PCA creates a set of new, mutually uncorrelated variables —the PCs— that capture a large part of the variance in the original dataset.

For each of these new variables, samples are assigned a score, reflecting their position along that PC. Scores help visualise patterns or groupings among samples on the PCA space. Loadings, conversely, are the weights that describe the contribution of each original variable (e.g., wavenumber) to a given PC. They indicate which regions of the spectrum (i.e., specific wavenumbers) are most influential in defining this PC. Loadings are critical for interpreting which molecular vibrations or functional groups are responsible for the observed variance. Loadings in the study of spectroscopic data are to be read as a spectrum, these are the loading spectra.

We performed PCA on each dataset, obtaining explanatory loading spectra and score plots for each training dataset. For each exploratory PCA, we extracted 10 PCs. For the PCAs conducted on each dataset, between 2 PCs and 5 PCs accounted for 95% of the variance. Score plots are visible on **Fig. S16**.

We performed a series of robustness tests before selecting the number of PCs to use as classification features.

1.3. Robustness analyses and feature selection

1.3.1 Robustness analysis

Outlier removal.

PCA and classification analyses are sensitive to outliers, data points that strongly differ from the majority. The exclusion of these outliers is recommended to avoid skewing subsequent

modelling. For each dataset, we tested the Hotelling's T^2 (sum of the normalised squared PCA scores) versus Q residual values (sum of square of each sample in the PCA error matrix) (*52,67*). Hotelling's T^2 measures how far a sample lies from the centre of the model in the principal component space, whereas the Q residuals quantify how much of the sample's variance is unexplained by the model, indicating samples that do not fit well within the model structure (e.g., possibly corrupted data) (*52*). Outliers are identified based on statistical thresholds. The threshold for T^2 values is based on the Chi-square distribution at a confidence level of 99% (alpha=0.99) for the number of components, retained whereas the threshold for Q residual is set at the 99th percentile of the Q value. Data points larger than these thresholds are consider outliers in our dataset. Samples detected as outliers (**Fig. S17**) were excluded from the dataset, then the PCAs, scree plots, and stability analyses were recompiled. The resulting retained PCs were subsequently used as classification variables.

Variance explained by the PCs.

To help select the appropriate number of PCs for classification, we first observed how much variance was accounted for ('explained') by each component and the cumulative variance (the sum of the variance of individual PCs) using scree plots (**Fig. S17**). An optimal number of PCs represents a maximum of Cumulative Variance (CV), and a maximum amount of Explained Variance (EV) for each PC. A good trade-off can be estimated between the slope change in Cumulated Variance and 95%. A 95% Cumulative Variance threshold strikes a balance between retaining meaningful information and reducing dimensionality. This ensures that most of the dataset's variability is preserved whilst filtering out noise and redundant features found in higher PCs, which account for very low variance. This selection can be further refined by the stability analysis and loading inspection.

Scree plots for each dataset can be seen in **Fig. S18**. For *Prototaxites* vs Bacteria (**Fig. S18a**), the Explained Variance (EV) decreases dramatically from PC1 (EV 78%) to PC2 (EV 10%); together these PCs account for 88% of the total variance (CV 88%). Beyond this, PC3 accounts only for 5% of EV, and with PC4 EV drops to less than 3%, and so is highly susceptible to variation due to noise. Therefore, the optimal number of PCs to be considered for further selection is between 2 and 4 (CV 95%).

For *Prototaxites* vs Plants (**Fig. S18b**), the Explained Variance decreases notably from PC1 (EV 59%) to PC2 (EV 18%), then more gradually from PC2 to PC3, and PC3 to PC4 (EV 11% and 5%, respectively); together these four PCs account for 93% of the total variance. PC5 accounts only for 2% of EV. Therefore, the optimal number of PCs to be considered for this dataset is between 2 and 5 (CV 95%).

For *Prototaxites* vs Fungi (**Fig. S18c**), the Explained Variance decreases notably from PC1 (EV 57%) to PC2 (EV 18%), then more slowly from PC2 to PC3, PC3 to PC4, and PC4 to PC5 (EV 12%, 5%, and 3%, respectively), together these five PCs account for 95% of the total variance. Therefore, the optimal number of PCs to be considered for this dataset is between 2 and 5 (CV 95%).

Finally, for *Prototaxites* vs Chitinous Organisms (**Fig. S18d**), the Explained Variance decreases notably from PC1 (EV 54%) to PC2 (EV 19%), then more slowly from PC2 to PC3, PC3 to PC4, and PC4 to PC5 (EV 11%, 7%, and 3%, respectively), together these five PCs account for 94% of the total variance. PC6 is less than 2% EV. Therefore, the optimal number of PC to be considered for this dataset is between 2 and 5 (CV 94%).

Stability analyses.

In addition to the scree plots, we estimated the optimal number of PCs to be retained on the basis of their stability to change in the dataset. In a stable PCA, the initial PCs usually present stronger stability compared to later PCs, as they reflect real patterns in the data variance rather than noise. Stability can be tested by resampling the dataset to allow rows to be selected with replacement (bootstrapping) and calculating the cosine angle between each resampled PC and the original PCs (*66*). A cosine angle of 1 indicates that the PCs in the original and resampled dataset are identical.

We performed bootstrap resampling for the PCA of each dataset. Results after outlier removal can be seen in **Fig. S19**. For *Prototaxites* vs Bacteria (**Fig. S19a**), only the first two PCs are very stable and should be considered for further selection. For *Prototaxites* vs Plants (**Fig. S19b**), *Prototaxites* vs Fungi (**Fig. S19c**), and *Prototaxites* vs Chitinous Organisms (**Fig. S19d**), the stable PCs are PC1 to PC5. These analyses support the estimations from the variance analysis detailed above.

1.3.2. Feature selection for classification

New variables obtained from PCA after robustness tests and outlier removal can be implemented as features for classification, constituting agnostic features as they are directly produced from the unsupervised PCA. Retaining only the most stable PCs removes complexity from the analysis and precludes the influence of noise, but the variables retained must be representative of the dataset variance and biologically informative. To maximise this trade-off, we inspected the loadings for the PCs which account for 95% of the variance, at the conditions where they were stable after outlier removal, for each individual dataset. This represents the first two PCs for Bacteria vs *Prototaxites,* and the first five PCs for the other datasets.

The loading spectrum for PC1 in Bacteria vs *Prototaxites* (**Fig. S20a**) reflects global aliphatic concentration, with a major influence from the 2800-3000 cm^{-1} region, and the same is true for PC1 in *Prototaxites* vs Plants (**Fig. S20b**). PC2 in Bacteria vs *Prototaxites* (**Fig. S20a**) and PC2 for *Prototaxites* vs Plants (**Fig. S20b**) are influenced by the common absorption of carbonyl moieties at 1650 cm^{-1}, coupled with the bands from nitrogen products at 1575, 1560 and 1540 cm^{-1}. Together these bands reflect the contribution of sugar-protein fossilisation products to PC2 for these datasets (*39*).

Variables influencing PC1 and PC2 for *Prototaxites* vs Fungi (**Fig. S20c**) and *Prototaxites* vs Chitinous Organisms (**Fig. S20d**) are similar to those for the two firsts datasets. PC1 is here influenced by the contribution of sugar-protein fossilisation products (reflecting the original organismal composition rich in amino-glucan), whereas PC2 reflects aliphatic contributions. On PC2, we also see a strong peak at 1590 cm^{-1} attributed to aromatic compounds. Together, these two contributions certainly reflect the general organic framework (aliphatic and aromatic contributions).

On PC3 for *Prototaxites* vs Plants (**Fig. S20b**), *Prototaxites* vs Fungi (**Fig. S20c**), and *Prototaxites* vs Chitinous Organisms (**Fig. S20d**), the main contribution is due to aromatic carbons (1590 cm^{-1}), but without an aliphatic contribution, suggesting that reflects a difference in the aromatic composition of the sample. On PC4 for these datasets, the main contribution is the band at 1540 cm^{-1}, indicating the contribution of carboxylate or nitrogen moieties.

Finally, PC5 for *Prototaxites* vs Plants (**Fig. S20b**), *Prototaxites* vs Fungi (**Fig. S20c**), and *Prototaxites* vs Chitinous Organisms (**Fig. S20d**), shows a contribution of bands at 1490 and 1680 cm^{-1}, which is associated with the silica matrix (silica overtone). This reflects a fine

difference in the silica content of the spectra and is not biologically informative. Therefore, PC5 should not be retained for classification.

Based on this visual inspection, we retained the first two PCs for *Prototaxites vs* Bacteria and the first four PCs for other datasets for classifier training and modelling (Part 2 of the pipeline).
On each of these PCs we selected the spectral bands with the highest loading coefficient. Here, we consider the bands for aliphatic CH_2 and CH_3 along with the bands for the fossilisation products of sugar-protein (carbonyl, carboxylate, and nitrogen products). We retained their intensity in the original spectra, to be used as explanatory variables for the Canonical Correspondence Analysis (CCA; section 1.4.).
These bands are inherently correlated with each other, which can limit their use in data exploration methods and interpretations. Correlated variables can be used in CCA only if the interpretation of the results is restrained to the correlation of each variable with the axes and not their weight (*68,69*). However, using intercorrelated individual bands instead of uncorrelated variables, for example obtained by PCA, will skew classification tasks (e.g., *52*).

1.4. Canonical Correspondence Analysis (CCA)

After an unsupervised exploration of the data (outlined in sections 1.2 and 1.3), we performed supervised exploration to investigate links between the lineages and their spectral features, which include ordination and correlation analyses.

To test Hypothesis 1, Canonical Correspondence Analyses (CCA) was conducted on the features selected above using the full dataset (D1). We wanted to visualise the correlation between the lineages and the spectroscopic variables (the most representative absorption bands).

CCA is a multivariate supervised statistical technique designed to explore the correlation between a matrix of response variables and a set of explanatory variables (*68,69*). To achieve this, CCA constructs ordination axes that are constrained to be linear combinations of the explanatory variables. CCA creates ordination diagrams where response variables and samples are represented as points and explanatory variables as vectors. **Fig. S21** helps visualise the factors (the spectroscopic features) which drive the differences between lineages.

The CCA (**Fig. S21**) uses the most informative bands as revealed by the loading spectra of the PCAs (**Fig. S20**) and shows a strong correlation between the fossilisation products of sugar-proteins (Amide I, II, N-products and carboxylate) and fungi, arthropod, peronosporomycete and amoeba lineages. A strong contribution of nitrogen products and carbonyl groups can also be expected for lineages with an amino-glucan derived composition in case of selective preservation, as chitin is a polymer of acetyl glucosamine ($C_8H_{15}NO_6$). On the other hand, bacteria show a strong correlation with aliphatic CH_x moieties. *Prototaxites*, and to a lesser extent the plants, shows a negative correlation with all these products.

The CCA (**Fig. S21**) confirms that each lineage correlates with different spectral features representing biological information, validating Hypothesis 1 (H1), and demonstrating that further classification can be performed to support the difference between *Prototaxites* and other Rhynie chert fossil taxa.

In summary, Part 1 of our analysis found that the molecular fingerprints of Rhynie chert organisms retained information regarding their biological affinity, supporting H1. We also generated datasets without outliers and constrained the PCA parameters for dimension reduction and feature selection, which we then used in our classification approach in Part 2.

2. Modelling (Classification)

Classification is a supervised machine learning task to predict the category to which a given input belongs based on previously trained data. The process involves splitting the dataset into three subsets: a training set, a validation set, and a test set.

The training set trains the model by teaching it the patterns and relationships in the data. The validation set then helps fine-tune model parameters and avoids overfitting by evaluating the model's performance on unseen data during training. For small datasets such as this one, cross-validation is used, whereby the dataset is split into multiple folds, and the model is trained and validated iteratively on different folds, ensuring better generalisation. When training is complete, the test set assesses the model's performance on completely new data. The test set is essential in machine learning as it objectively evaluates a model's ability to generalise to unseen data. By assessing performance on the test set, we ensure the model can handle new data effectively rather than simply memorising patterns in the training data and overfitting. The test set remains

untouched until the final evaluation phase, offering an independent and objective measure of the model's predictive power. Misusing the test set, such as incorporating it into training or model selection, compromises its purpose and can lead to overly optimistic performance estimates.

We performed two types of classification: multi-class classification and one-class classification. In multi-class classification, the model discriminates among multiple possible classes (e.g., *Prototaxites* and Fungi). In one-class classification, the model identifies whether data points belong to a single target class (e.g., *Prototaxites*) or are outliers (e.g., any other fossils).

For multiclass classification, we have used Linear Discriminant Analysis (LDA) and Support Vector Machine (SVM).

LDA is a classification technique which finds linear combinations of features to maximise the separation between classes, whilst minimising within-class variance. It works well for linearly separable data that follow a normal distribution. However, LDA is robust enough to handle spectroscopic data when variable selection and reduction are applied beforehand (e.g., with PCA) (*52*), and especially so for small datasets. For visualisation, we have included the ordination graph of each training sets, which is presented as a one-dimensional diagram as the models are binary.

SVM is a versatile classification algorithm that finds an optimal hyperplane to separate classes by maximising the margin between them. It can handle linear and non-linear data using kernel functions and is robust to high-dimensional spaces. We implemented our SVM using a Radial Basis Function (RBF) kernel, which is a very flexible kernel (*88*). Cross-validation is used to define the best kernel hyperparameters. For two-dimensional visualisation, we included the decision boundary calculated on a two PC iteration of our model.

One-class classification was used to test Hypothesis 4 (H4) with Soft Independent Modelling of Class Analogy (SIMCA). We will now describe each of these approaches in turn in, starting with a description of our approach to model selection, imbalanced dataset correction and model evaluation.

2.1. Multi-class classification.

2.1.1. Parsimony principle

Classification modelling should be conducted in order of parsimony, beginning from the simplest and moving through increasingly complex algorithms (*52*). This order also implies testing models that base their discrimination on linear correlations before testing non-linear correlations. For example, LDA should be performed before SVM. If the results of one classifier are not satisfactory (e.g., performance is not robust), then the complexity of the model can be increased. Ensuring satisfactory results for a model implies validation or cross-validation of the results obtained for the training set and obtaining satisfactory results for the external test set. A similar performance on both the validated training set and the test set demonstrates the model's robustness.

2.1.2. Imbalanced dataset

Imbalanced datasets occur when the classes in a dataset are not represented equally and could lead to biased model performance where the majority class is favoured. We have addressed this issue using Synthetic Minority Oversampling Technique (SMOTE). SMOTE addresses the issue of imbalance by generating synthetic examples for the minority class rather than duplicating existing ones or relying solely on loss adjustments (weighting), enabling better decision boundaries, and reducing model bias (*73*).

2.1.3. Model evaluation

Cross-Validation.

Analysing fossils within a single assemblage can limit the number of relevant targets that can be utilised. Here, we required well-preserved fossils located at the surface of the thin sections and belonging to taxonomic groups of relevance to the study. Previous study of the Rhynie chert fossils[5] demonstrate that this approach is successful at producing meaningful spectroscopic data. To ensure proper validation on this limited dataset, we used resampling cross-validation algorithms instead of a validation set (*74*). We performed Leave-One-Out Cross Validation (LOOCV) which consists of training the model on all the training data samples bar one, then the model is tested on the sample that had been left out. This process is repeated until all samples

have been used as tests. LOOCV is particularly suited for our analyses because the time needed to compute the cross-validation is not a limitation when the dataset is small. Once done, the model is retested on the complete training dataset. The performance of the cross-validating training data will be compared to the test data results to assess the model's robustness.

Learning curves.

To demonstrate that dataset size does not impede the model's robustness, we computed the learning curve for each analysis. We computed the learning curves by testing the model performance first on 10% of the training dataset, then added iteratively 10% of the dataset until the whole training dataset was used; with a five-fold cross-validation (i.e., 80% of the training set was used to train the model, and 20% of it to test the model). The resulting curves represent how model accuracy evolved with the amount of data in the training set. Learning curves for the LDA models, and then the SVM models will be discussed below.

Linear Discriminant Analysis (LDA) learning curves

Fig. S22 shows the learning curves for each LDA model for each dataset. These curves illustrate how model accuracy improves with increased training set size up to the full dataset. Every training accuracy curve starts high (near 1.0), indicating overfitting when the dataset is small. The curve then decreases slightly as training set size increases, which is expected as the model generalises better with more training data. Similarly, validation accuracy is initially low with a large variance, reflecting model instability due to a small training dataset. The validation accuracy increases, and its variance decreases with more training data, meaning better generalisation. These behaviours reflect a decrease in overfitting as the number of samples in the training set increases.

The dataset Bacteria vs *Prototaxites* (**Fig. S22a**) shows training and validation accuracy reaching 100% almost immediately and stabilising. The dataset is very small, and the model is possibly overfitting even with the full dataset in that configuration (e.g., 5-fold cross validation). However, bacteria and *Prototaxites* present very distinctive spectral signatures relative to each other, which was identified by qualitative and exploratory analyses. A complex model to discriminate between them would likely provide no further insight.

The dataset Plant vs *Prototaxites* (**Fig. S22b**) shows a training accuracy beginning at 100%, then decreasing as dataset size increases. The validation accuracy starts low (~50%) but gradually improves with more data. This reflects a strong initial overfitting, however the gap between training and validation accuracy narrows with an increased dataset size, indicating the complete dataset is sufficient to draw meaningful conclusions. In absence of additional samples, Leave-One-Out Cross Validation (LOOCV), or a more complex model (e.g., SVM), could further reduce the variance and enhance the classification robustness.

The dataset Fungi vs *Prototaxites* (**Fig. S22c**) has a training accuracy that starts high (~100%) but decreases slightly with additional data. The validation accuracy fluctuates significantly in the early stages, showing high variance but, as the dataset grows, validation accuracy stabilises. The full dataset is sufficient to draw meaningful conclusions, as indicated by the narrow gap between the two curves, and the constrained variance.

The dataset Chitinous Organisms vs *Prototaxites* (**Fig. S22d**) has a training accuracy that starts at 100% and decreases slowly with more data. The validation accuracy starts low but steadily increases, eventually closely approaching training accuracy. The variance is originally high but reduces as the dataset grows. The model generalises better with more data and the full dataset is large enough for reliable analysis.

Overall, across all plots, the incomplete datasets lead to overfitting and high variance, but the use of a full training set improves generalisation and reduces the variance, making the model more robust.

Support Vector Machine (SVM) learning curves

Fig. S23 shows the learning curves for each SVM model. Each model shows a strong initial overfitting, but the gap between training and validation accuracy narrows with increased dataset sizes. The variance is relatively well constrained with the full dataset, indicating that the available datasets are adequate to draw meaningful conclusions, and we can see a clear improvement from the LDA models above. Our dataset *Prototaxites* vs Bacteria is too small to perform a robust SVM.

Stability tests.

To test the stability of our models, we performed bootstrapping stability tests on each dataset (100 iterations) using the cross-validated model. The bootstrap stability results show the sensitivity of the model to variation in the data. The results of each stability test are displayed for each analysis in section 2.1.4 alongside the results of LDA and SVM (**Figs. S24-30**)

Confusion matrices.

To show the discrimination performance of the models, we provide the confusion matrices for each model that displays the number of well-classified and misclassified samples. It provides the number of True Positive (TP), True Negative (TN), False Positive (FP), and False Negative (FN), which are defined as:

True Positive (TP): Correctly predicting a sample as non-*Prototaxites*.
True Negative (TN): Correctly predicting a sample as *Prototaxites.*
False Positive (FP): Incorrectly predicting a sample as non-*Prototaxites* when it is actually *Prototaxites.*
False Negative (FN): Incorrectly predicting a sample as *Prototaxites* when it is actually non-*Prototaxites.*

Confusion matrices for each dataset are displayed for each analysis in section 2.1.4 alongside the results of LDA and SVM (**Figs. S24-30**).

Performance metrics.

To evaluate the performance of each model, we calculated the following metrics:

- Accuracy: The measure of the proportion of correctly classified instances (both *Prototaxites* and non-*Prototaxites*) among all instances.
- Precision: The measure of the proportion of samples predicted as non-*Prototaxites* that are actually non-*Prototaxites*.
- Recall (Sensitivity): The measure of the proportion of non-*Prototaxites* instances correctly predicted as non-*Prototaxites*.

- F1: The harmonic means of precision and recall, providing a balanced measure.
- Matthews Correlation coefficient (MCC): A robust metric for binary classification that accounts for all four components of the confusion matrix (TP, TN, FP, FN). It ranges from -1 (perfect misclassification) to +1 (perfect classification), with 0 indicating random guessing.

We aimed to minimise differences between performance metrics values for the cross-validating training sets and the test sets because. Similar performances for both sets indicate that the model generalises to new unseen data without either underperforming, which suggests overfitting, or overperforming, which could suggest underfitting or random guesses rather than true performance. The results for each metric and each set are displayed on radar plots in **Figs. S24-30**. Having outlined the complete list of performance metrics used, we will now describe the results for each dataset, and for the different modelling approaches. This is separated into two sections. First our investigation of whole spectra (2.1.4), which is separated into results for the LDA and then SVM; then our tests on individual absorption bands (2.1.5).

2.1.4. Whole spectra analyses (PC features)

LDA performance metrics

Prototaxites vs Bacteria (**Fig. S24**)*:*

LOOCV Accuracy on Resampled Training Set: 1.00

LOOCV Precision on Resampled Training Set: 1.00

LOOCV Recall on Resampled Training Set: 1.00

LOOCV F1-Score on Resampled Training Set: 1.00

LOOCV Matthews Correlation Coefficient (MCC) on Resampled Training Set: 1.00

Test set Accuracy: 1.00

Test set Precision: 1.00

Test set Recall: 1.00

Test set F1-Score: 1.00

Test set Matthews Correlation Coefficient (MCC): 1.00

Prototaxites vs Plants (**Fig. S25**)*:*

LOOCV Accuracy on Resampled Training Set: 0.83

LOOCV Precision on Resampled Training Set: 0.87

LOOCV Recall on Resampled Training Set: 0.83

LOOCV F1-Score on Resampled Training Set: 0.83

LOOCV Matthews Correlation Coefficient (MCC) on Resampled Training Set: 0.71

Test set Accuracy: 0.93

Test set Precision: 0.94

Test set Recall: 0.93

Test set F1-Score: 0.93

Test set Matthews Correlation Coefficient (MCC): 0.85

Prototaxites vs Fungi (**Fig. S26**)*:*

LOOCV Accuracy on Resampled Training Set: 0.81

LOOCV Precision on Resampled Training Set: 0.86

LOOCV Recall on Resampled Training Set: 0.81

LOOCV F1-Score on Resampled Training Set: 0.81

LOOCV Matthews Correlation Coefficient (MCC) on Resampled Training Set: 0.67

Test set Accuracy: 0.82

Test set Precision: 0.88

Test set Recall: 0.82

Test set F1-Score: 0.82

Test set Matthews Correlation Coefficient (MCC): 0.69

Prototaxites vs Chitinous Organisms (**Fig. S27**)*:*

LOOCV Accuracy on Resampled Training Set: 0.85

LOOCV Precision on Resampled Training Set: 0.89

LOOCV Recall on Resampled Training Set: 0.85

LOOCV F1-Score on Resampled Training Set: 0.85

LOOCV Matthews Correlation Coefficient (MCC) on Resampled Training Set: 0.74

Test set Accuracy: 0.79

Test set Precision: 0.88

Test set Recall: 0.79

Test set F1-Score: 0.80

Test set Matthews Correlation Coefficient (MCC): 0.63

SVM performance metrics

Prototaxites vs Plants (**Fig. S28**)*:*

LOOCV Accuracy on Resampled Training Set: 0.90

LOOCV Precision on Resampled Training Set: 0.91

LOOCV Recall on Resampled Training Set: 0.90

LOOCV F1-Score on Resampled Training Set: 0.9

LOOCV Matthews Correlation Coefficient (MCC) on Resampled Training Set: 0.81

Test Set Accuracy: 0.93

Test Set Precision: 0.93

Test Set Recall: 0.93

Test Set F1-Score: 0.92

Test Set Matthews Correlation Coefficient (MCC): 0.83

Prototaxites vs Fungi (**Fig. S29**)*:*

LOOCV Accuracy on Resampled Training Set: 0.94

LOOCV Precision on Resampled Training Set: 0.94

LOOCV Recall on Resampled Training Set: 0.94

LOOCV F1-Score on Resampled Training Set: 0.94

LOOCV Matthews Correlation Coefficient (MCC) on Resampled Training Set: 0.88

Test Set Accuracy: 0.91

Test Set Precision: 0.93

Test Set Recall: 0.91

Test Set F1-Score: 0.91

Test Set Matthews Correlation Coefficient (MCC): 0.83

Prototaxites vs Chitinous Organisms (**Fig. S30**)*:*
LOOCV Accuracy on Resampled Training Set: 0.96
LOOCV Precision on Resampled Training Set: 0.96
LOOCV Recall on Resampled Training Set: 0.96
LOOCV F1-Score on Resampled Training Set: 0.96
LOOCV Matthews Correlation Coefficient (MCC) on Resampled Training Set: 0.92
Test Set Accuracy: 0.93
Test Set Precision: 0.94
Test Set Recall: 0.93
Test Set F1-Score: 0.93
Test Set Matthews Correlation Coefficient (MCC): 0.85
The classification models built using LDA and subsequently improved with SVM answered H2, that *Prototaxites* fundamentally differs in its fossilisation products from those of the fungi and chitinous organisms in the Rhynie chert. Our analyses also answered H3 by demonstrating that the fossilisation products of *Prototaxites* also differ from those of bacteria and plants in the Rhynie chert.

2.2. One-class modelling
In order to support our discriminant models and test whether *Prototaxites* is not only molecularly different from bacteria, plants, and chitinous organisms but also distinct from all Rhynie chert fossils taken together, we performed a One-Class Classifier (OCC) on the full dataset (D1).

OCC analyses are conducted by modelling only one class of a dataset, in order to test if new data belongs to the modelled class. The most used algorithm for OCC is Soft Independent Modelling of Class Analogy (SIMCA), specifically its Data-Driven approach (DD-SIMCA) (*75,77,89*). DD-SIMCA builds a class-specific model using Principal Component Analysis (PCA) for the target class, allowing for the identification of new samples by their fit to this model, while accommodating data variability and dimensionality reduction.

We performed DD-SIMCA using the mda.tools Web-application for DD-SIMCA developed by Kucheryavskiy and colleagues (*77*) available at www.mda.tools/ddsimca.

Model training.

We trained a PCA model on the *Prototaxites* samples (n=12). To mitigate the effects of small sample size, we use resampling Leave-One-Out Cross-Validation, a rigorous feature selection (PCs) approach based on sensitivity, and less permissive outlier detection using robust parameter estimates on the training set[11,12]. Following the method of Kucheryavskiy et al. (*75*), we identify outliers in the middle range of PCs, which correspond to 5 PCs. In DD-SIMCA, objects are categorised as regular (inlier samples), extreme (inlier samples with extreme values), and outlier (bad samples). The decision limits between the regular and extreme (95%) and extreme and outlier (1%) can be visualised on an acceptance plot. After robust outlier detection, the log-acceptance plot for our model (**Fig. S31**) shows no detected outlier. We then selected the optimal number of PCs to be retained for future prediction tasks based on the performance of the model.

Sensitivity (the true positive rate) is the main performance metric for OCC models, which measures the proportion of actual positive cases correctly identified by the model. A sensitivity of 1 or 100% indicates there are no false negatives. The sensitivity plot for the *Prototaxites* dataset (**Fig. S32**) shows overfitting starting at three PCs. According to this plot, a model using two PCs is optimal (sensitivity = 0.917). Using these two PCs, we proceeded with the prediction task.

Prediction.

To demonstrate how *Prototaxites* differs from other Rhynie chert organisms, we tested the whole fossil dataset (all other fossils) using the model trained on *Prototaxites* samples.
The results of the classification are shown on the log-acceptance plot (**Fig. S33**). Specificity (the true negative rate) reflects performance of the model on the test set. A specificity of 1 or 100% indicates that the model makes no mistakes.

The model provides an excellent specificity (true negative rate) of 0.911, indicating high confidence that *Prototaxites* differs from all other Rhynie chert fossil in its fossilisation products.

The OCC analysis allows us to accept H4, that the molecular fingerprint of *Prototaxites* is unique in the Rhynie chert.

3. Conclusion

Our analyses aimed at contrasting the molecular composition of *Prototaxites* fossils with those of other Rhynie chert fossils (fungi, arthropods, plants, bacteria, amoebae, and peronosporomycetes). To achieve this, we conducted a two-step approach based on data exploration and modelling that relied upon a single taphonomic assumption – that all the Rhynie chert fossils have undergone a similar diagenetic history - and minimised the arbitrary choice of variables. Using CCA for ordination and correlation, we demonstrated that the molecular composition of the fossils in the Rhynie chert are correlated with their biological lineages (H1). In particular, fungi, arthropods, peronosporomycetes and amoebae are strongly correlated with the features for fossilisation products of sugar-protein complexes.

Using PCA for dimension reduction, and robustness analyses of variance, stability, and spectral exploration for variable selection, we built robust classification models using LDA for D1 and SVM for D2-D4. These models strongly discriminated the molecular composition of *Prototaxites* from those of fungi and chitinous organisms together, confirming H2. The models also discriminated *Prototaxites* signals from those of plants and bacteria (H3). Finally, using OCC we demonstrated that the molecular composition of *Prototaxites* is unique in the Rhynie chert, addressing H4.

These results support the findings from our descriptive palaeontological investigations. *Prototaxites* was clearly distinct from fungi in its morphology, anatomy, and molecular composition, and is therefore incompatible with classification as a fungus.

Supplementary Figures

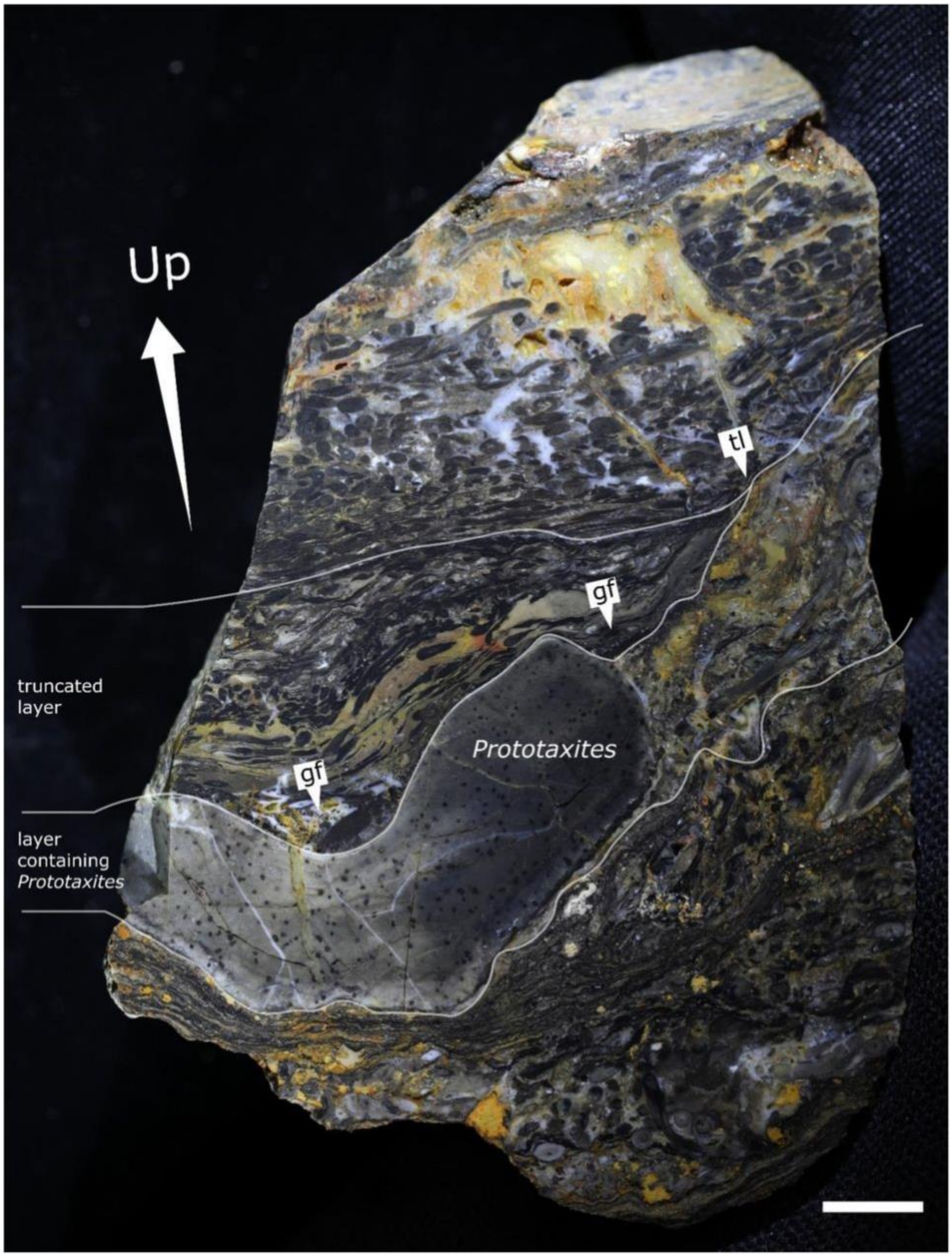

Fig. S1. Establishing the orientation of NSC. 36. Annotated view of Rhynie chert block NSC.36 after being cut in half to show the location of the specimen relative to surrounding sediment and interpret its orientation. Selected laminations and geopetal features, which support the way-up direction indicated: tl = truncated lamina; gf = geopetal sediment fill. Scale bar: 5 mm.

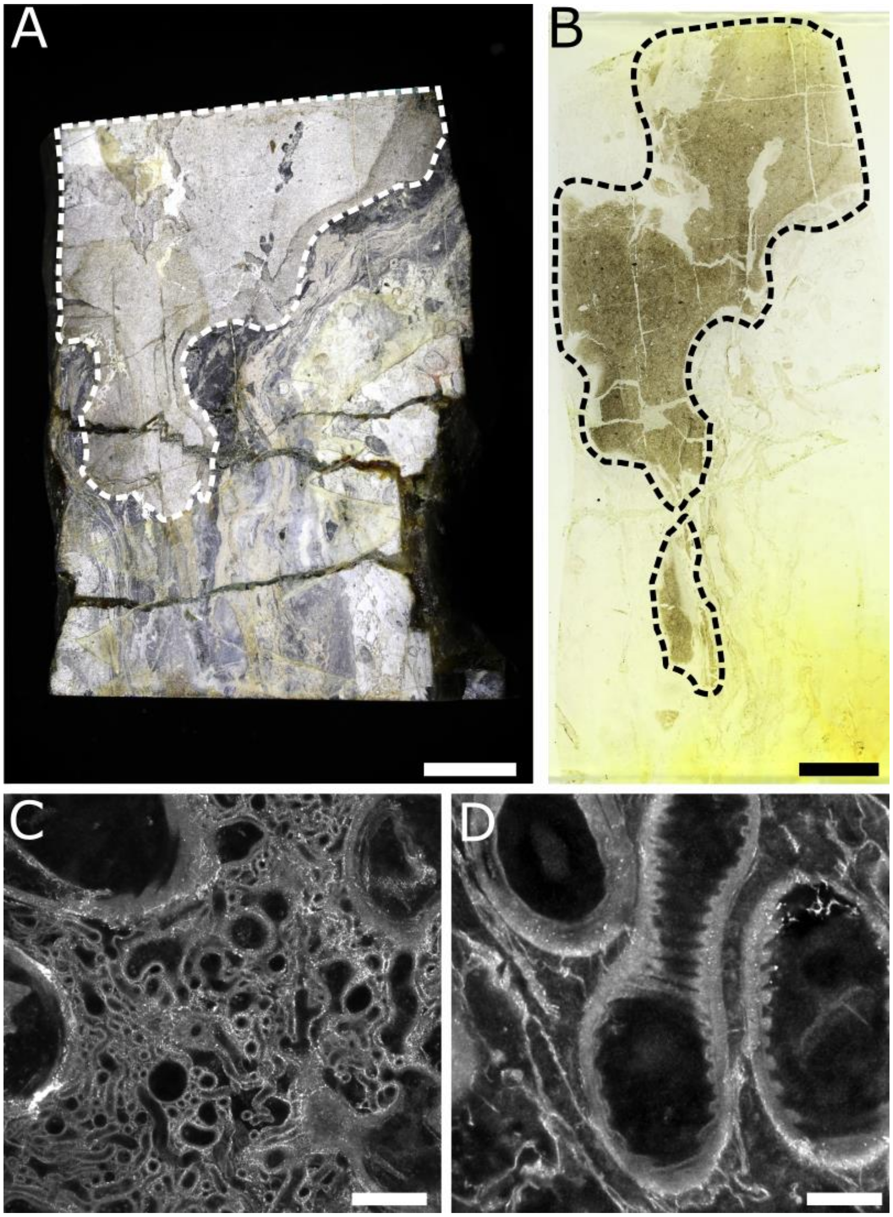

Fig. S2. Structural features of *Prototaxites taiti* from Lyon Block 156. A-**B**, Lyon 156 block (**A**) and peel (**B**) with *P. taiti* highlighted in dashed lines. **C-D**, Airyscan CLSM imaging of the spot region shown in Fig. 1F (**C**), Airyscan CLSM imaging of banded tubes in the body of Lyon 156, also from the slide shown in Fig. 1E (**D**). Scale bars: 1cm (**A**, **B**), 200µm (**C**), 20µm (**D**). Specimen accession codes: Lyon 156 (**A**), Lyon Peel 156/1 (**B**), Lyon 156 MPEG0078 (**C**-**D**).

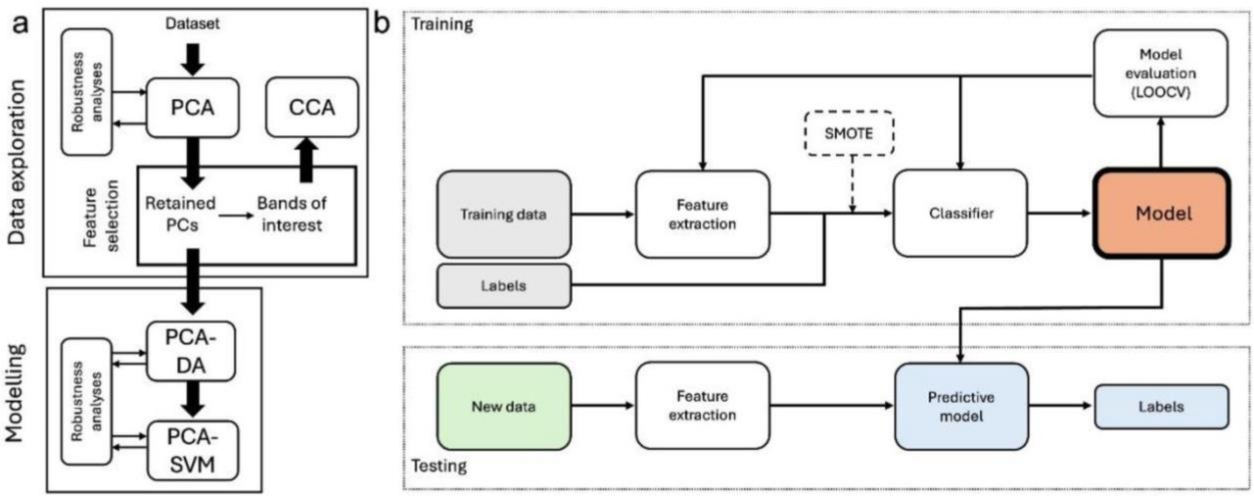

Fig. S3. Analytical pipeline used in this study. (**A**) illustrates the key steps of our workflow, comprising data exploration (dimension reduction and feature selection using Principal Component Analysis (PCA), and Canonical Correspondence Analysis (CCA) for correlation and ordination) and modelling using Linear Discriminant Analysis combined with PCA (PCA-DA) and Support Vector Machine combined with PCA (PCA-SVM). (**B**) details of the modelling steps with the splitting of the data for training and testing. Training consists of feature extraction (the PCA stage) followed by the correction for class-imbalance (SMOTE) if needed, and the choice of classifier (DA or SVM). The model obtained is cross-validated by Leave-One-Out-Cross-Validation (LOOCV). Testing consists of a feature extraction stage following the same parameters than the training stage and a prediction stage where the label (*Prototaxites* or plant, fungi, bacteria) for new, unseen instances in the test set are predicted by the cross-validated model.

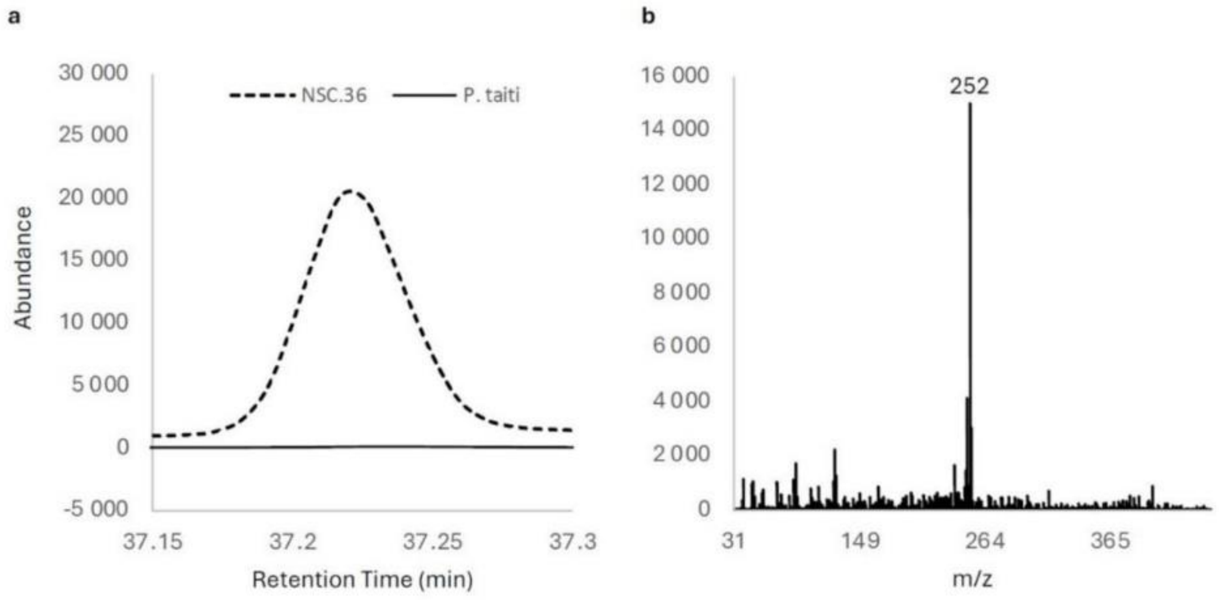

Fig. S4. Perylene was not identified in *Prototaxites taiti* but was detected in bulk NSC.36 material. a, Chromatogram trace showing possible ascomycete biomarker perylene peak with a retention time of ca. 37.22 minutes for the bulk NSC.36 material (dashed line) and the pure *P. taiti* material (solid line). Chromatogram was recorded in selected ion monitoring mode (SIM) at m/z 252 which is diagnostic for perylene. Only the relevant chromatographic region is shown. **b**, Mass spectrum of perylene showing diagnostic m/z 252 ion.

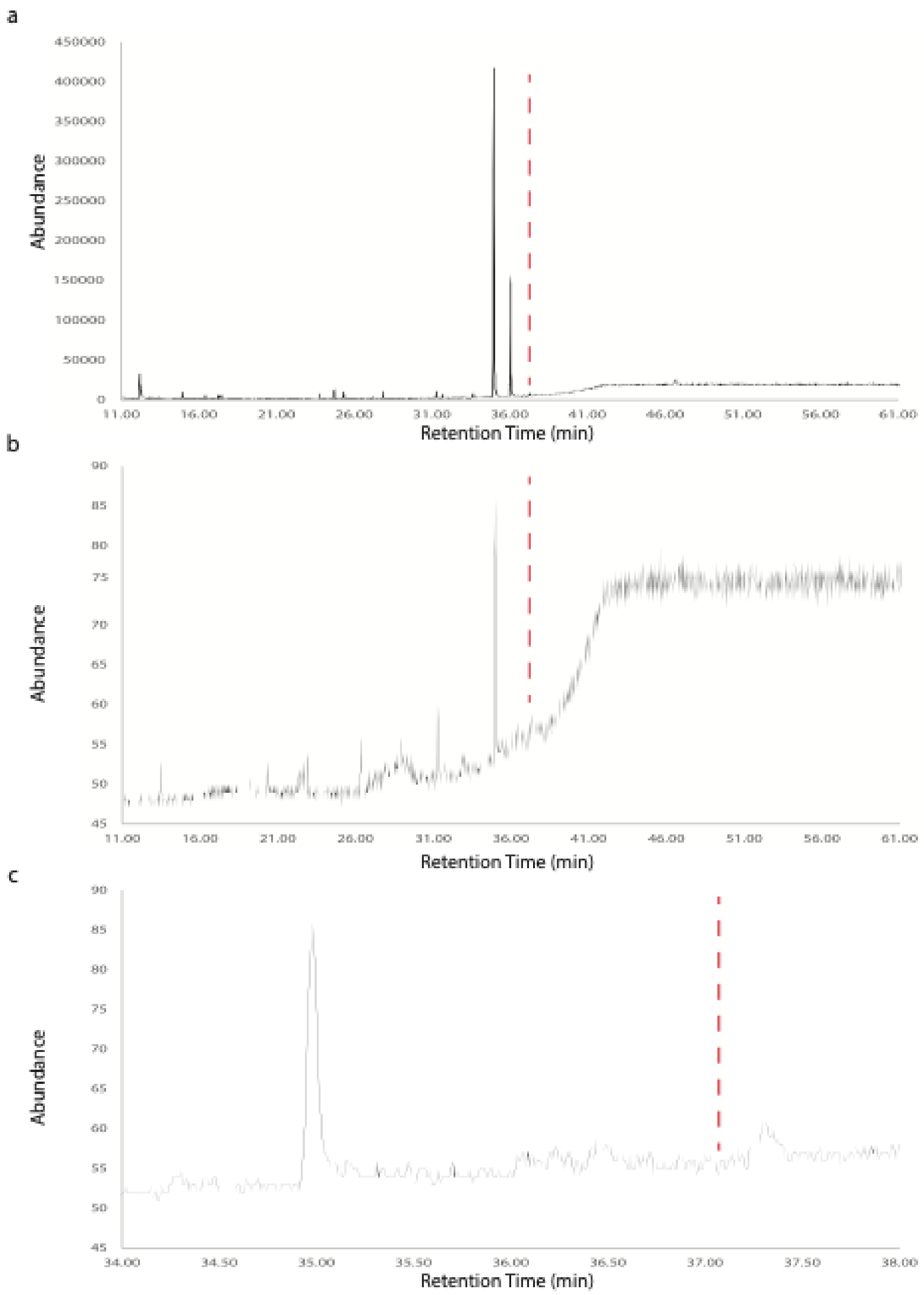

Fig. S5. GC-MS chromatograms for combusted brick control. a Total ion chromatogram, **b** selected ion monitoring at m/z 252 chromatogram, and **c** section of chromatogram in **b** displaying region of elution for perylene (34 to 38 minutes). The dashed red line in each chromatogram indicates the retention time for perylene (32.23 minutes) highlighting the absence of this molecule in the control.

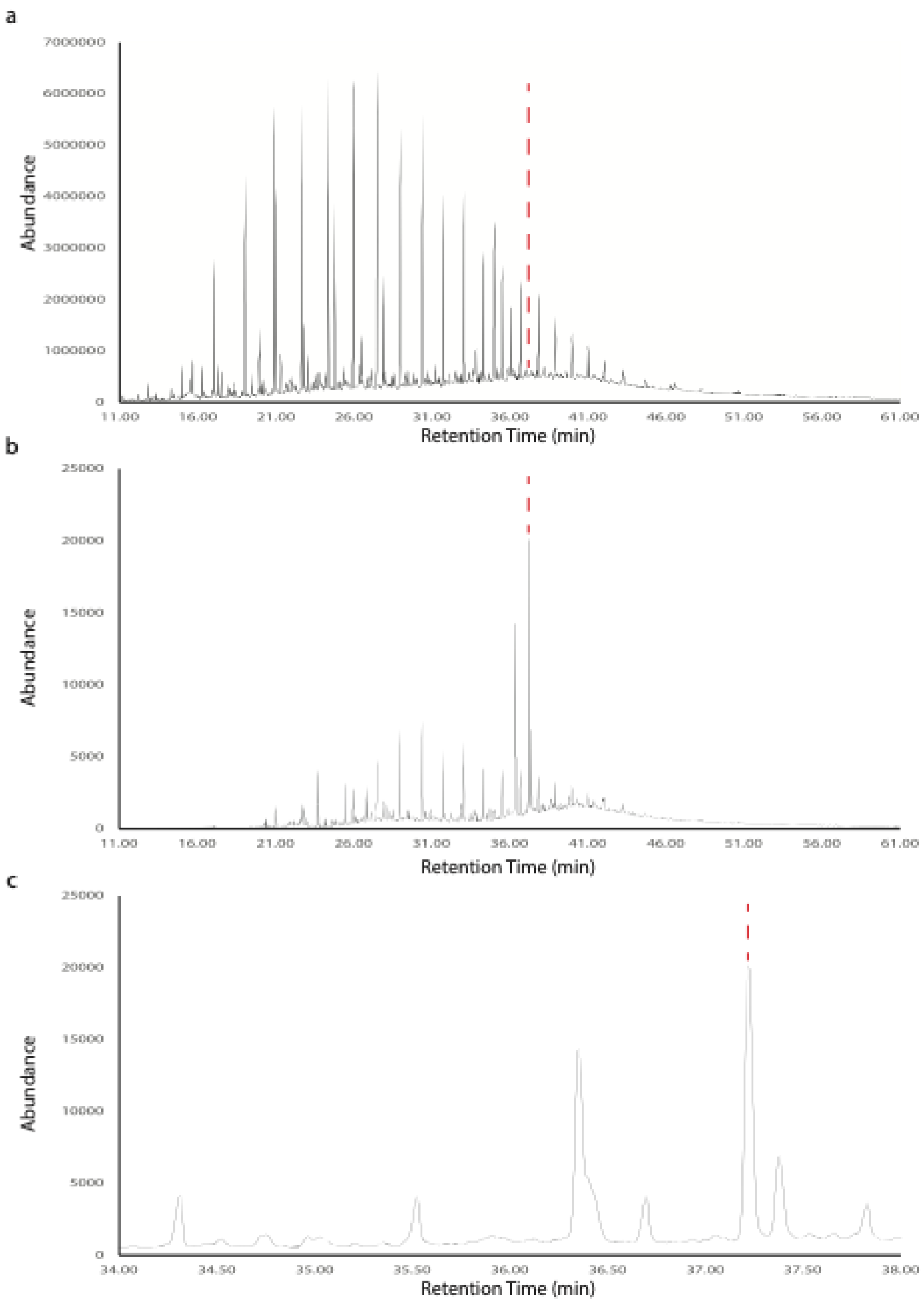

Fig. S6. GC-MS chromatograms for bulk Rhynie chert material. a Total ion chromatogram, **b** selected ion monitoring at m/z 252 chromatogram, and **c** section of chromatogram in **b** displaying region of elution for perylene (34 to 38 minutes). The dashed red line in each chromatogram indicates the perylene peak at retention time 32.23 minutes.

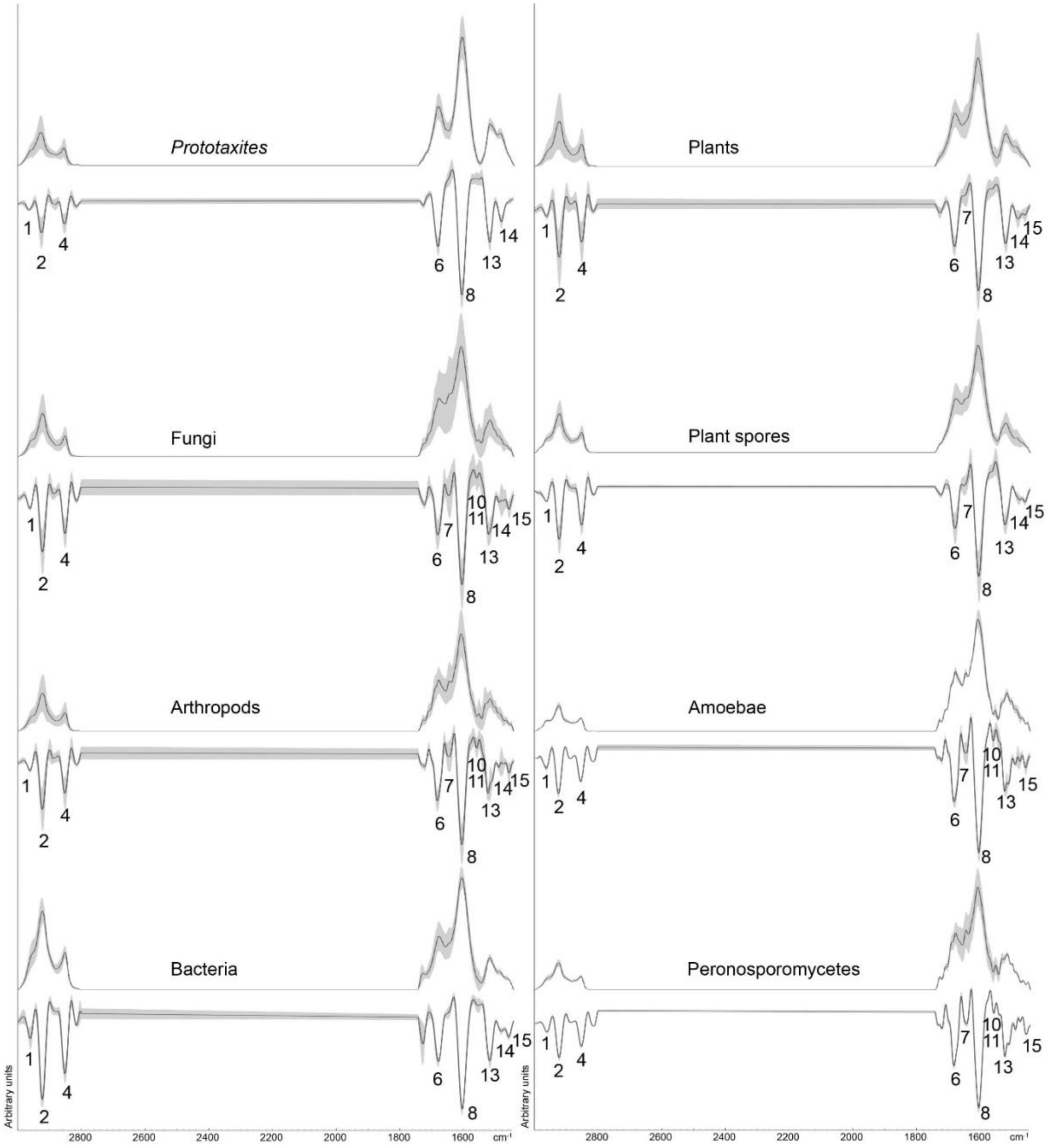

Fig. S7. Truncated and baselined ATR-FTIR spectra for each category. Absorption spectra are on top, and their respective second derivative spectra are at the bottom. Black lines are the average spectra; grey areas are the full variability for each category. Peak numbers correspond to the numbers in the first column of **Table S1**. Full spectra for all samples are provided in the **Auxiliary supplementary materials** (Datasets for FTIR analyses).

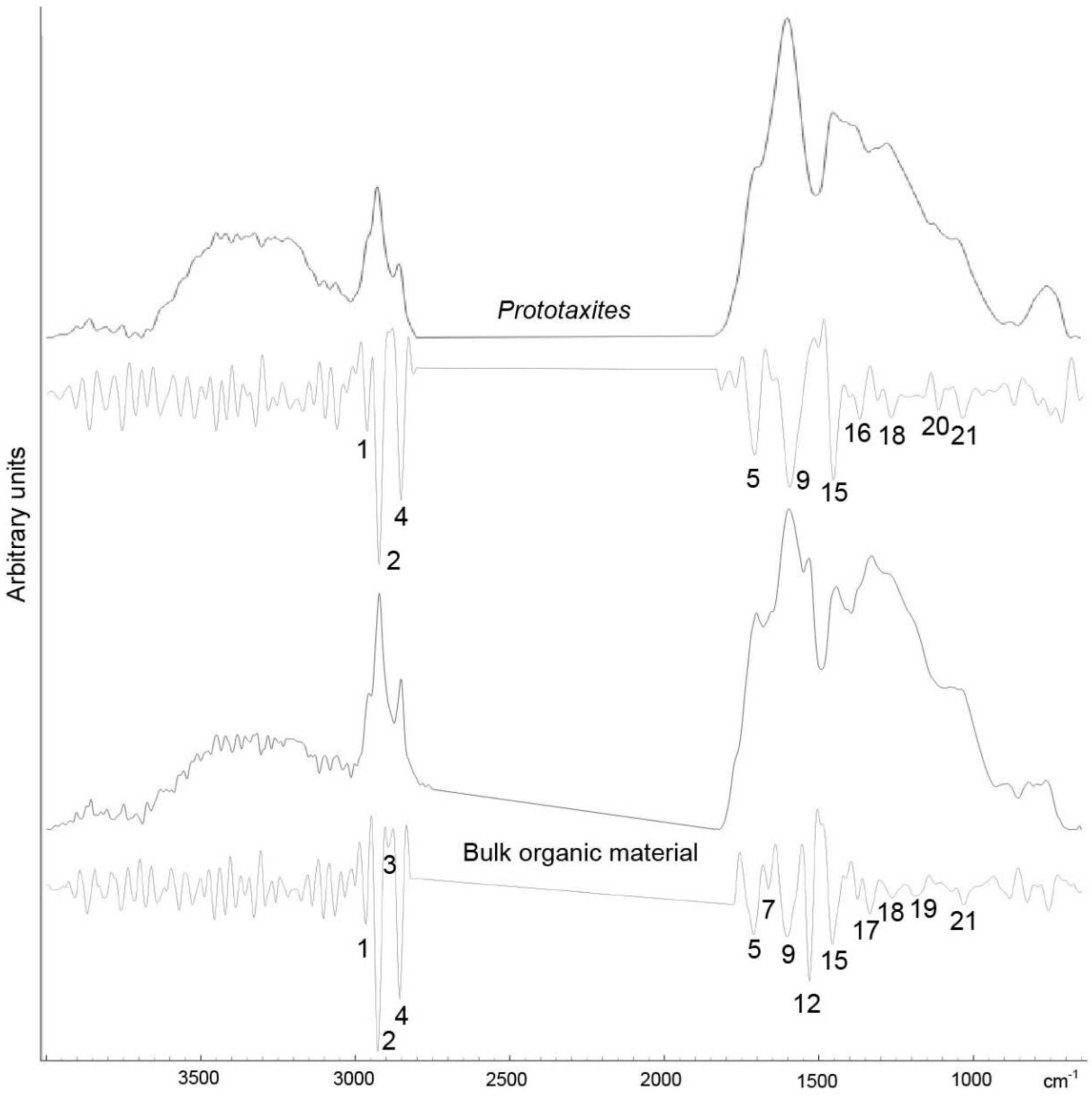

Fig. S8. Transmission FTIR spectrum for extracted *Prototaxites* material. Absorption spectrum is on top with its respective second derivative spectrum in the bottom**.** Peak numbers correspond to the numbers in the first column of **Table S1** below.

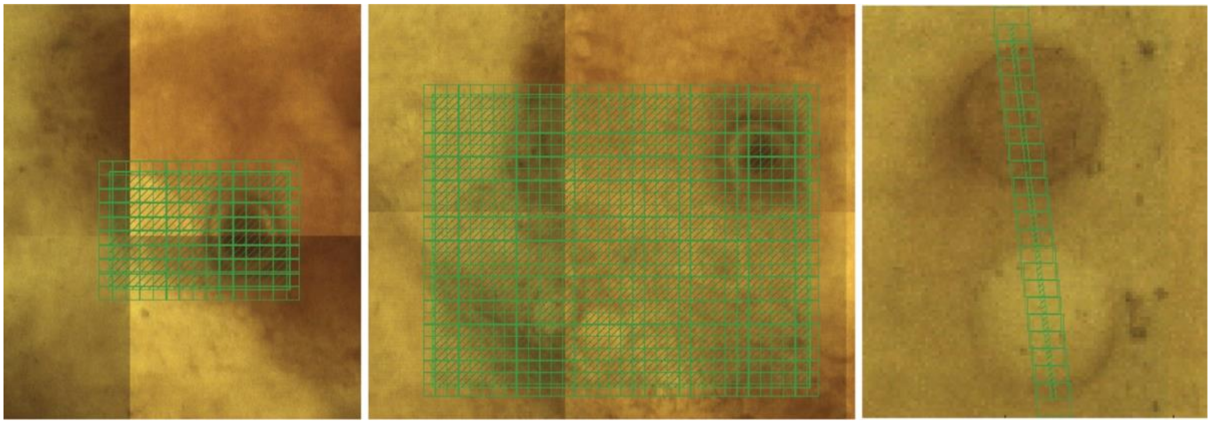

Fig. S9. Location of synchrotron maps and transect. Green squares correspond to each acquired spectrum (aperture of 10x10 microns and step of 5 microns). Darker brown areas correspond to medullary spots (images 1 and 2). Image 3 is of two transversal cuts through type 2 tube within the matrix.

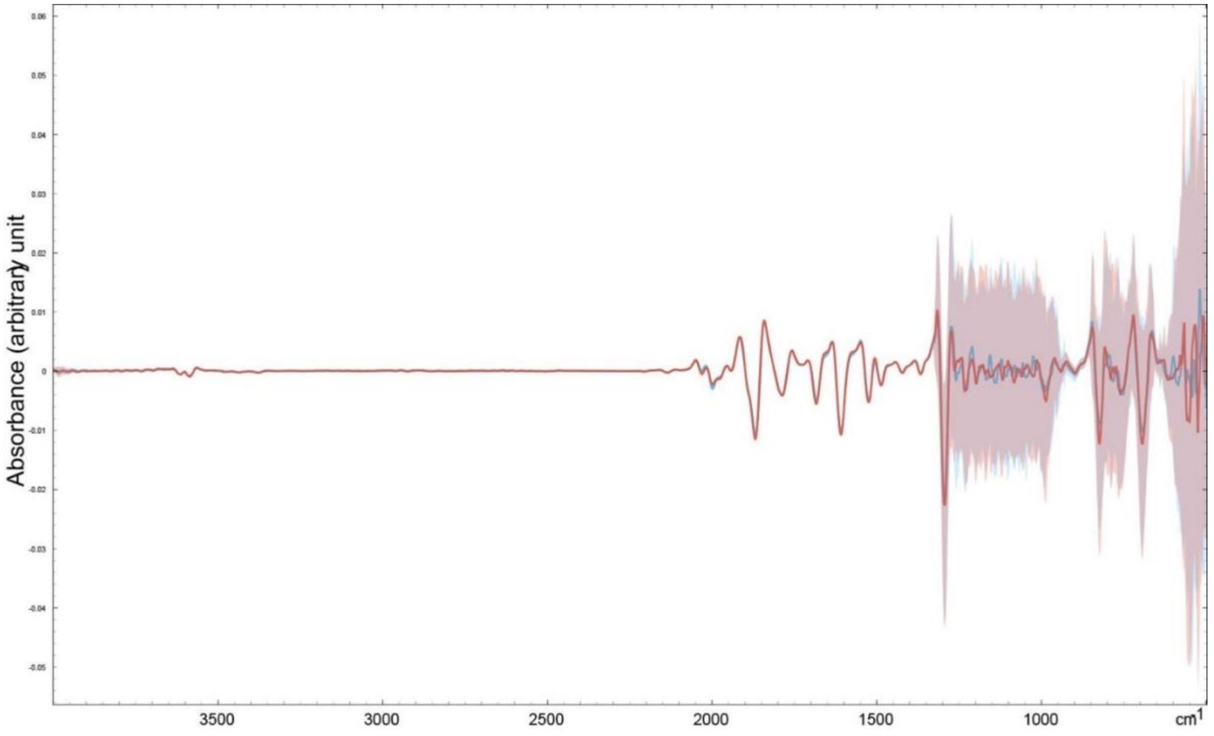

Fig. S10. Average raw second derivative synchrotron-FTIR Spectra for the main body and medullary spots. Spectra for the main body are in red and for medullary spots in blue. The spectra show the intense, saturating, signal of silica below 1450 cm^{-1}. The shaded areas correspond to the full spectral variation for each category.

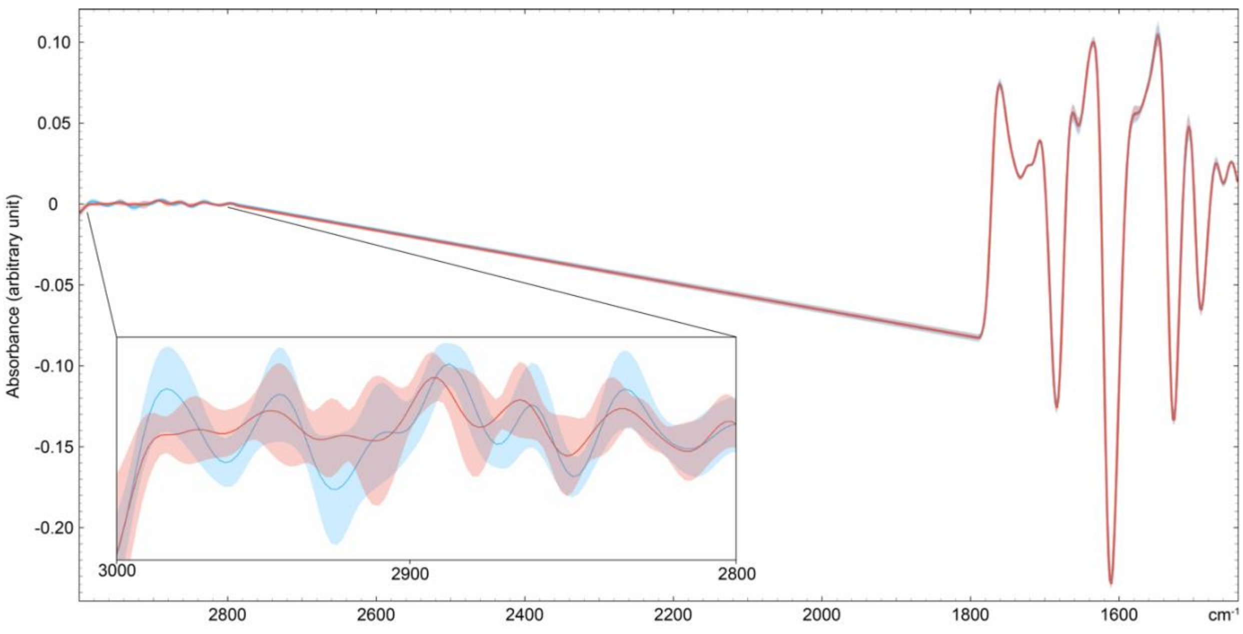

Fig. S11. Average pre-processed second derivative synchrotron-FTIR Spectra for the main body and medullary spots. Spectra for the main body are in red and for medullary spots in blue. Zoomed area corresponds to the aliphatic C-H region, where only small differences between the two types can be observed (below the signal-to-noise ratio). The shaded areas correspond to the full spectral variation for each category.

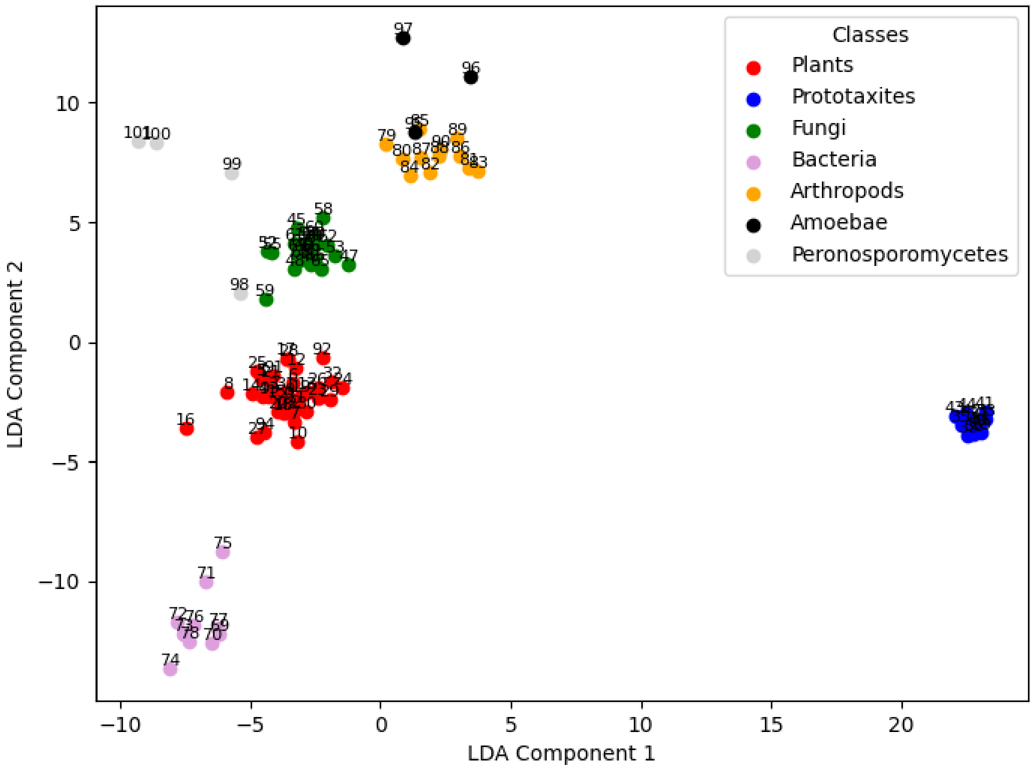

Fig. S12. Discriminant space projection for the full dataset (D1) without prior dimension reduction. The classes of fossils are clearly separating from each other, but this analysis represents separation based on uninformative spectral bands. This indicates the model is overfitting and that dimension reduction should be conducted.

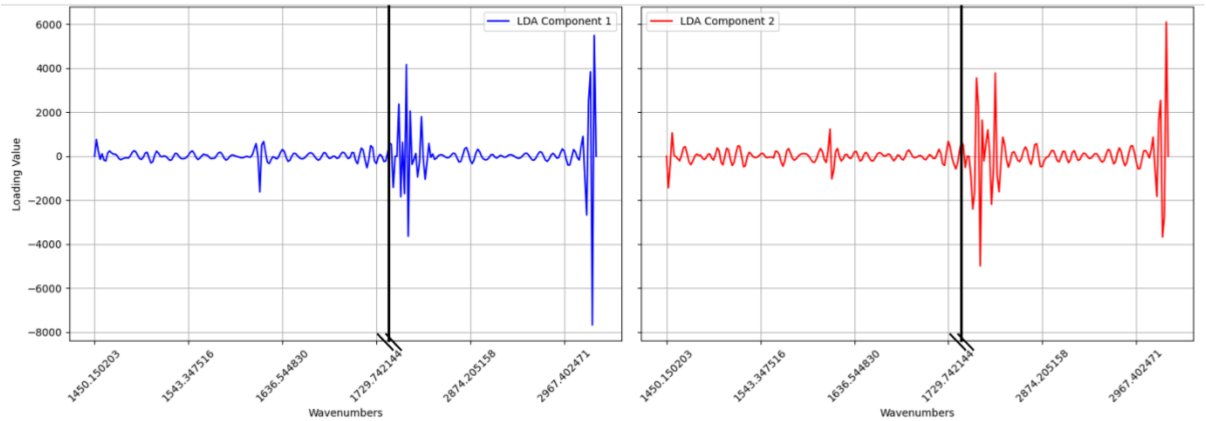

Fig. S13. Loading plots for the two discriminant components that contribute the most to Fig. S12. The variables that contribute the most to separation of the classes, at ca. 2800-2830 and 2980-3000 cm^{-1} on both components, do not reflect any real molecular contribution but rather a noise pattern (that is, uninformative spectral variations that do not correspond to any bond vibrations). This indicates the model is overfitting and that dimension reduction should be conducted.

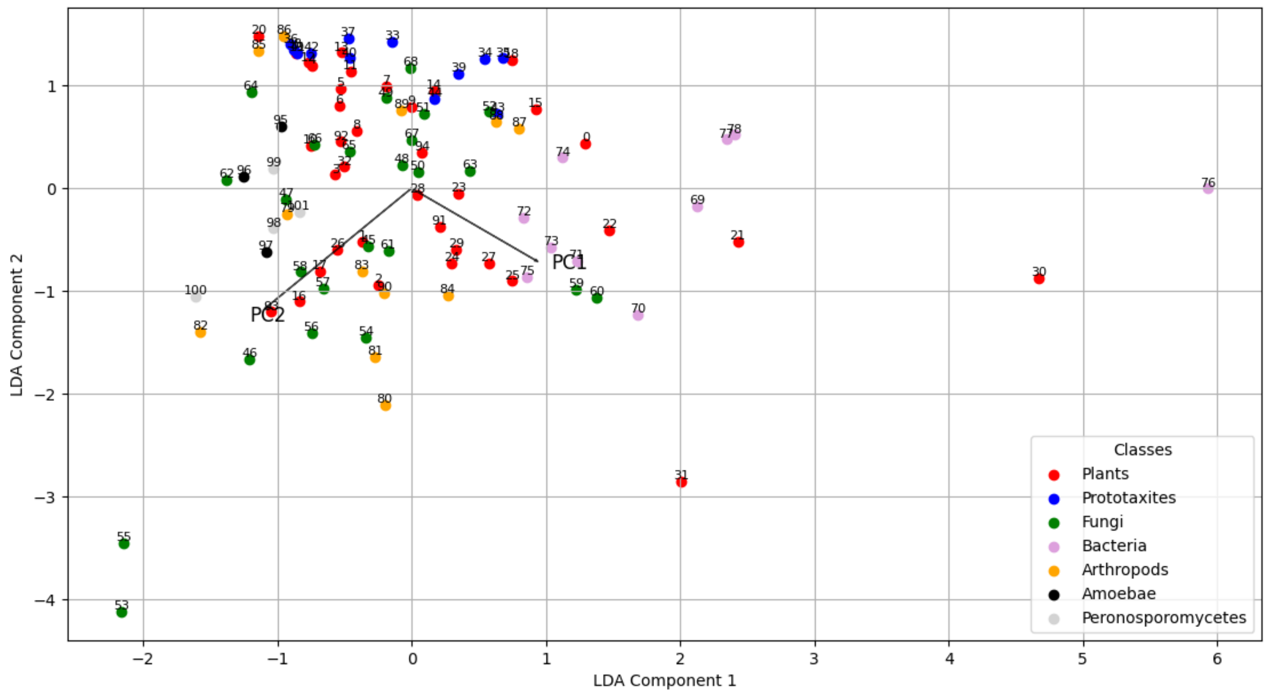

Fig. S14. Discriminant space projection for the full dataset with prior dimension reduction by PCA, eliminating correlation between multiple variables. The classes of fossils are overlapping, although some, like *Prototaxites* and Bacteria, remain constrained in space. The loading vectors (black arrows) show the contribution of each variable, here PC1 and PC2, to the projection of the data in space.

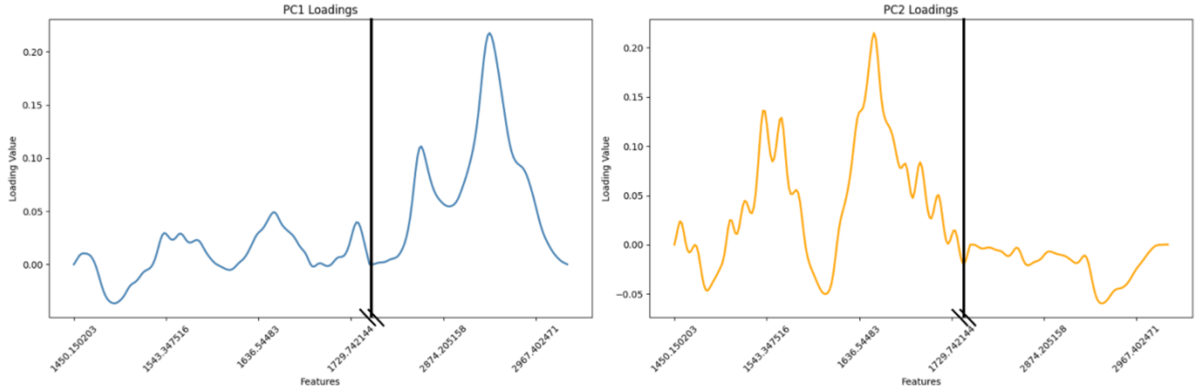

Fig. S15. Loading plots (loading spectra) for PC1 and PC2. These two PCs are responsible for the projection of the data and reflect spectral features that are biologically informative. PC1 loadings show that the main contribution to the first axis are the aliphatic moieties at ca. 2850, 2925 and 2960 cm^{-1}, whereas PC2 loadings reflects the influence of carbonyl C=O (1650 cm^{-1}) and nitrogen products (1540-1575 cm^{-1}), as expected for fossilisation products of sugar-proteins.

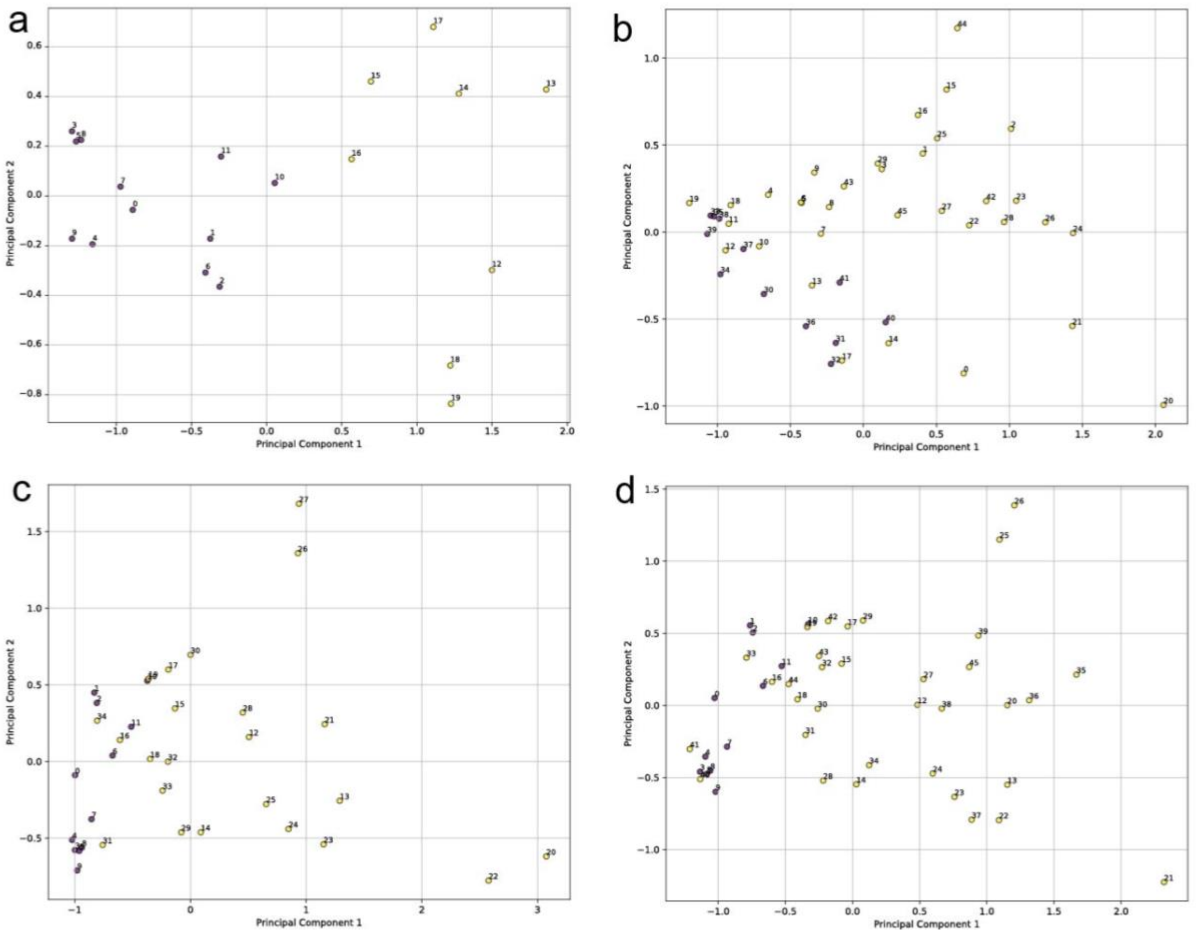

Fig. S16. Score plots for *Prototaxites* vs Bacteria (a), *Prototaxites* vs Plants (b), *Prototaxites* vs Fungi (c), *Prototaxites* vs Chitinous Organisms (d). In two dimensions, *Prototaxites* (purple in a-d) separates well from bacteria (yellow in a), but shows some overlap with other lineages (yellow in b-d), indicating the need to include more dimensions (PCs) in further analyses.

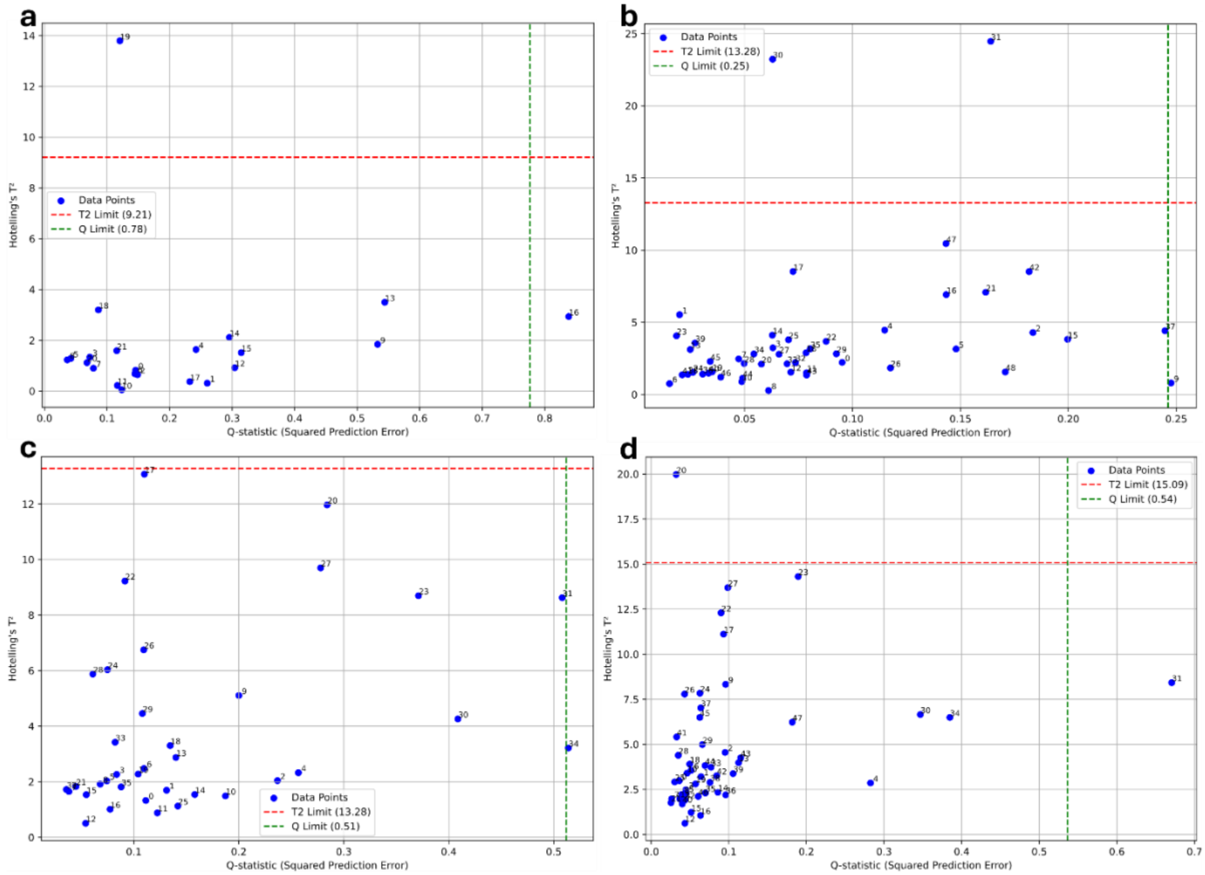

Fig. S17. Outlier detection test. Hotelling's T^2 vs Q residual for *Prototaxites* vs Bacteria (a), *Prototaxites* vs Plants (b), *Prototaxites* vs Fungi (c), *Prototaxites* vs Chitinous Organisms (d). Points are designated as outliers if located beyond the T2 (red dashed line) and Q (green dashed line) limits on the plots.

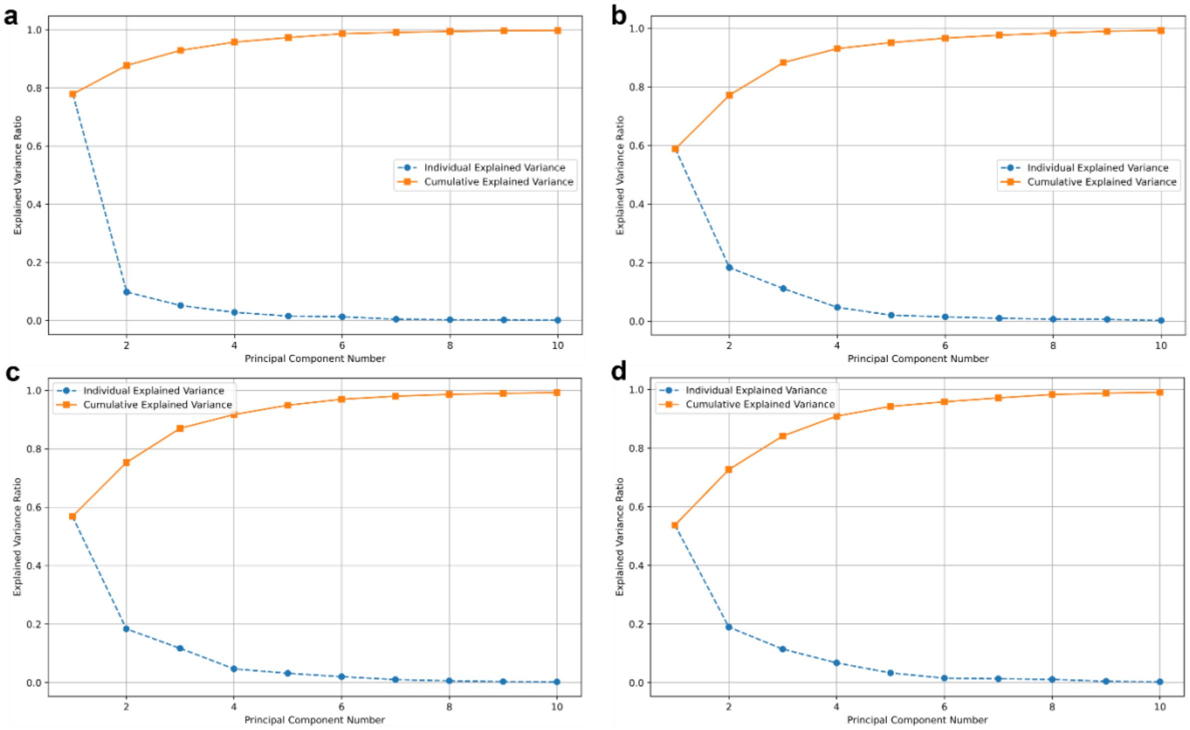

Fig. S18. Scree plots of PCA for *Prototaxites* vs Bacteria (a), *Prototaxites* vs Plants (b), *Prototaxites* vs Fungi (c), *Prototaxites* vs Chitinous Organisms (d) after outlier removal. An optimal number of PCs can be estimated to be between 2 to 5 for each dataset.

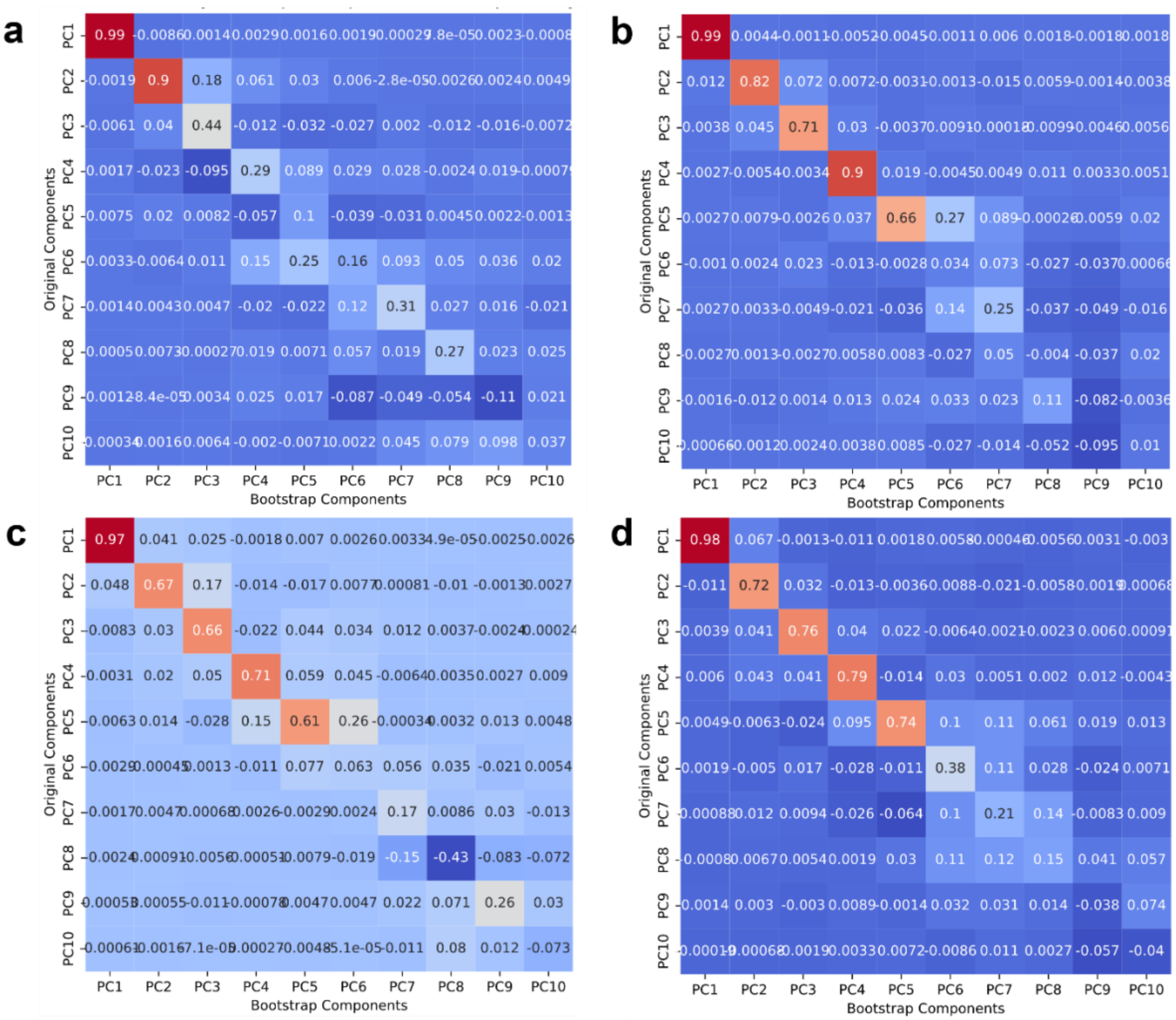

Fig. S19. Bootstrap stability analyses by cosine similarity of PCs for *Prototaxites* vs Bacteria (a), *Prototaxites* vs Plants (b), *Prototaxites* vs Fungi (c), *Prototaxites* vs Chitinous Organisms (d) after outlier removal. The most stable PCs are marked in red and represent a cosine angle of more than 0.65. Only these PCs were retained for classification**.**

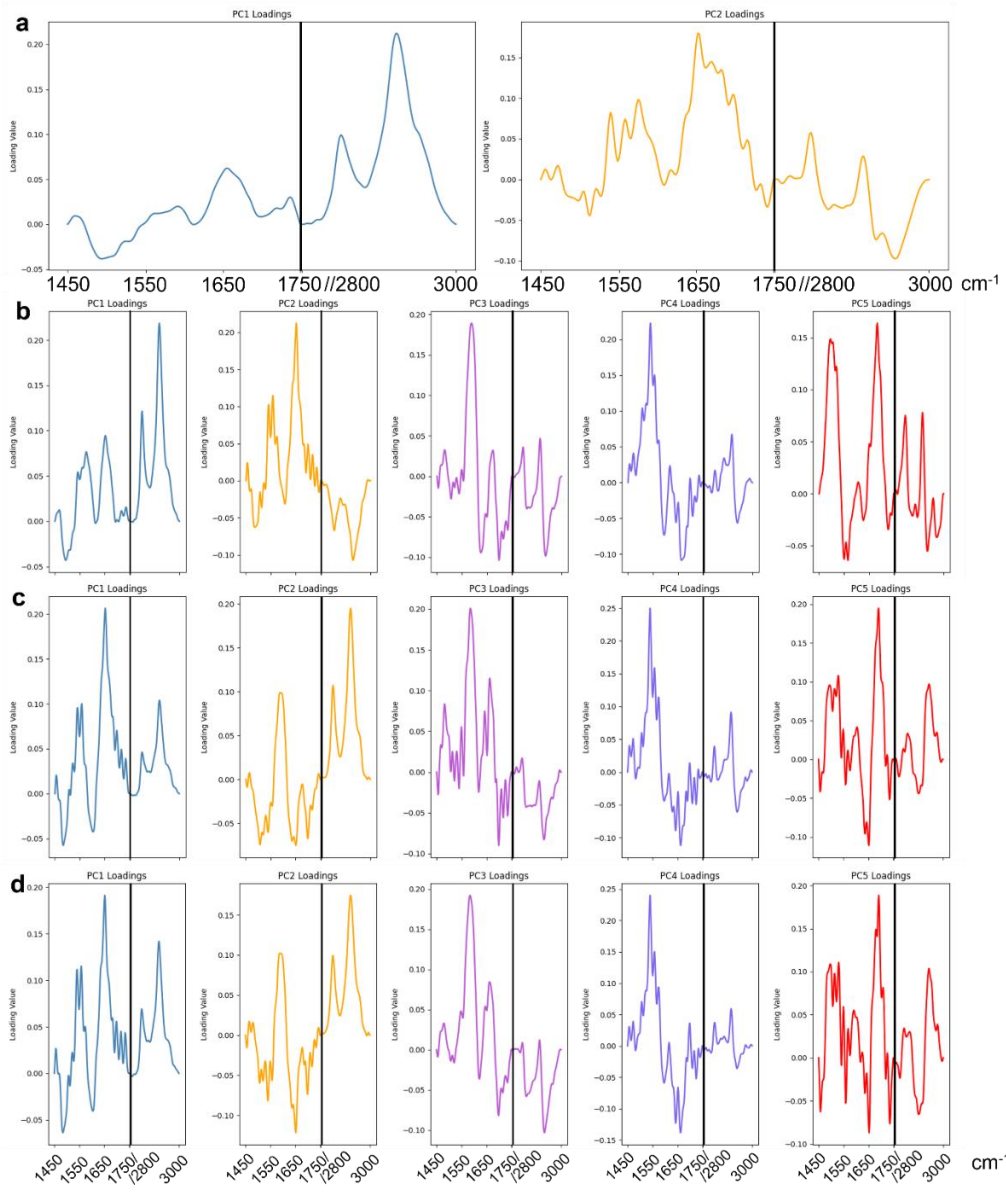

Fig. S20. Loading spectra of PC1 and PC2 for *Prototaxites* vs Bacteria (a), and PC1 to PC5 for *Prototaxites* vs Plants (b), *Prototaxites* vs Fungi (c), *Prototaxites* vs Chitinous Organisms (d).

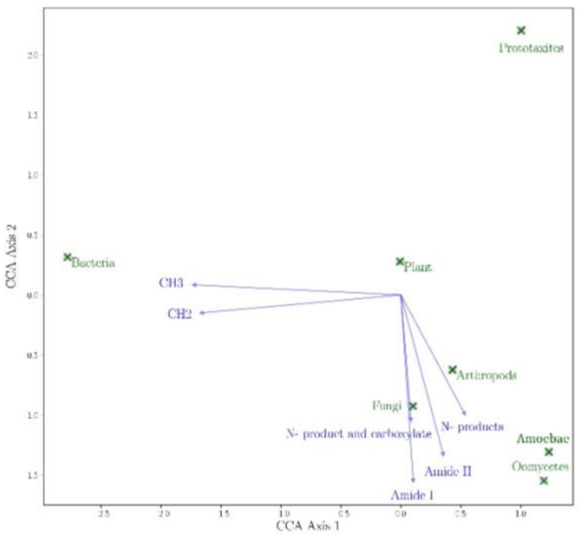

Fig. S21. Canonical Correspondence Analysis (CCA) ordination diagram for Rhynie chert lineages and representative absorption contributions. This is reproduced with annotation in main text *Fig.* 3.

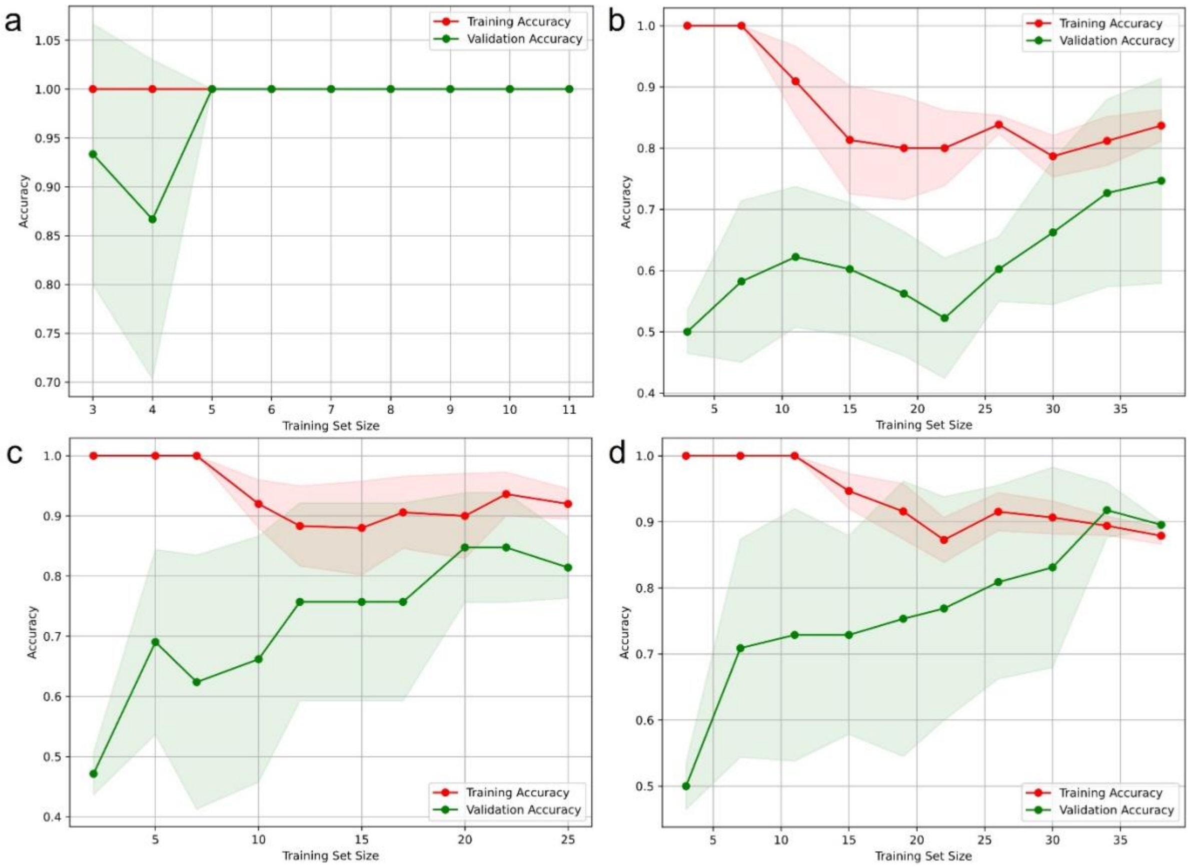

Fig. S22. Learning curves of LDA models. *Prototaxites* vs Bacteria (a), *Prototaxites* vs Plants (b), *Prototaxites* vs Fungi (c), and *Prototaxites* vs Chitinous Organisms (d).

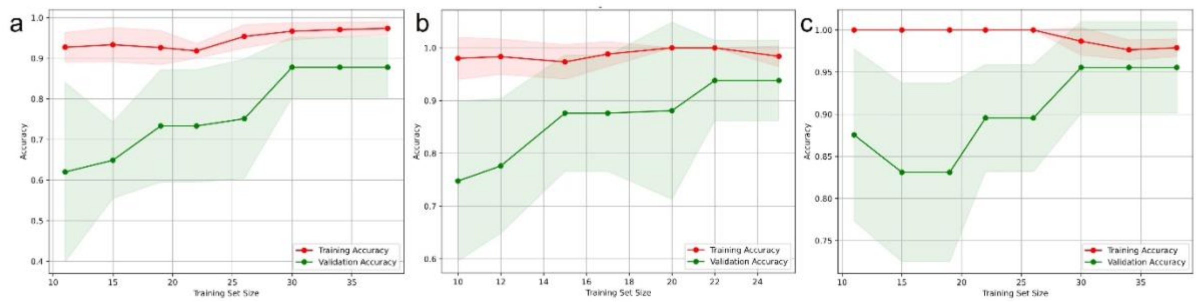

Fig. S23. Learning curves of LDA models. *Prototaxites* vs Plants (a), *Prototaxites* vs Fungi (b), and *Prototaxites* vs Chitinous Organisms (c).

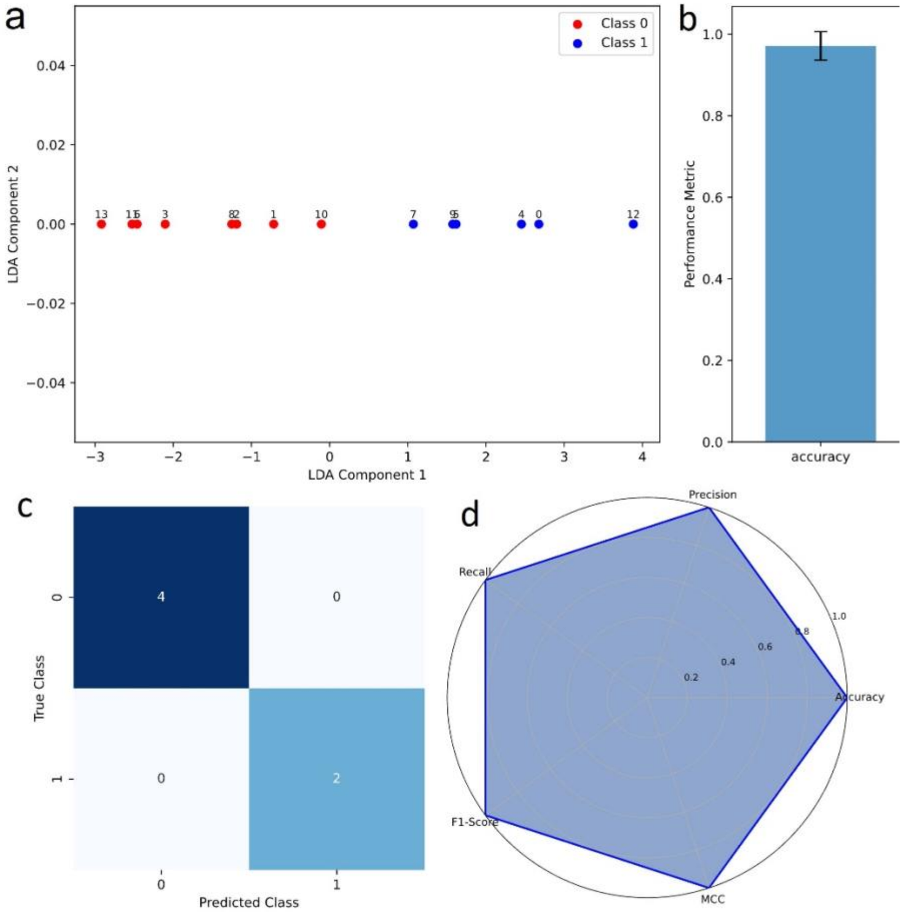

Fig. S24. Linear discriminant analysis after PCA dimension reduction of *Prototaxites* (class 0) vs Bacteria (class 1). The ordination score plot **(a)** shows an excellent separation along the LDA first component axis. The bootstrap stability analysis **(b)** shows an excellent resistance to changes in the dataset (mean= 0.97, std= 0.03). The confusion matrix **(c)** and performance metrics **(d)** report an excellent classification with all *Prototaxites* and bacteria samples correctly discriminated.

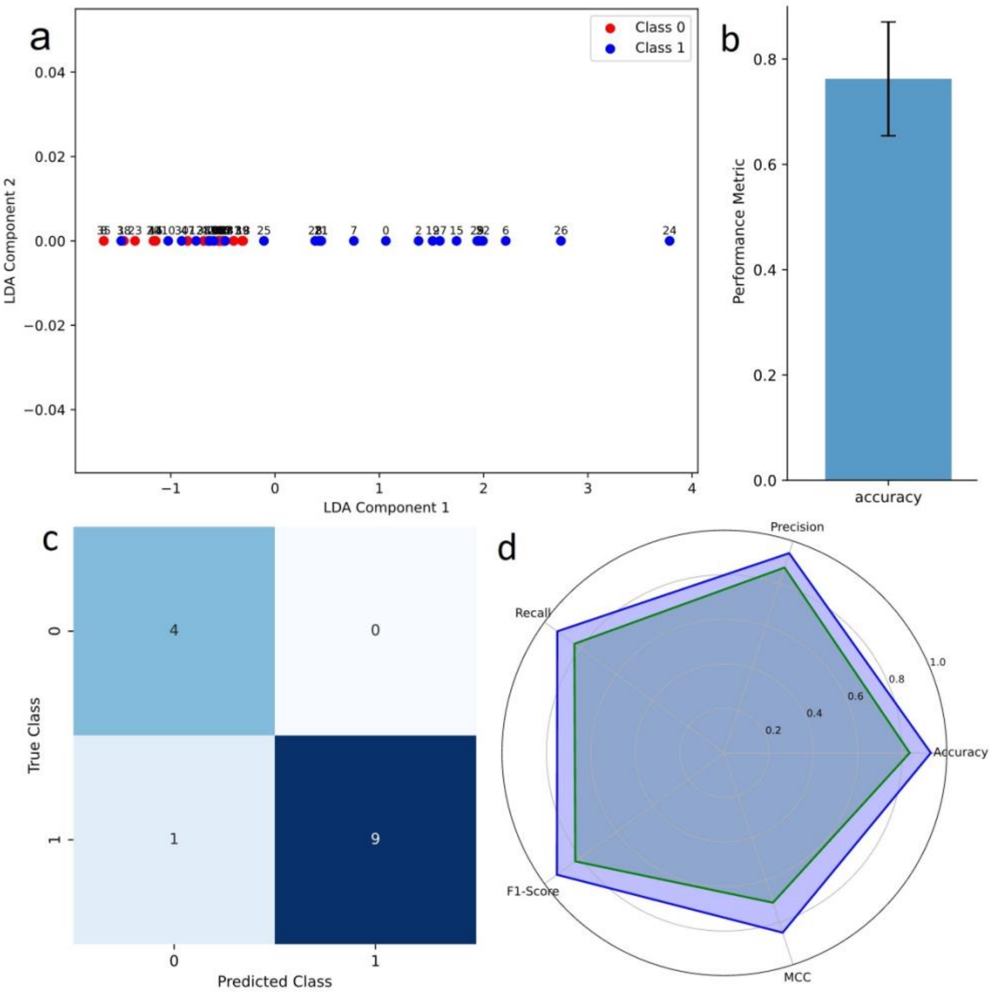

Fig. S25. Linear discriminant analysis after PCA dimension reduction of *Prototaxites* (class 0) vs Plants (class 1). The ordination score plot **(a)** shows a strong overlap along the LDA first component axis. The bootstrap stability analysis **(b)** shows a good resistance to changes in the dataset (mean= 0.76, std= 0.11). The confusion matrix **(c)** reports an excellent classification of the test set, with all *Prototaxites* correctly discriminated and only one plant sample misclassified. The performance metrics **(d)** show that the training set (green) is outperformed by the test set (blue), suggesting that the model is slightly underfitting. Performance could be improved by using a more flexible model (e.g., SVM).

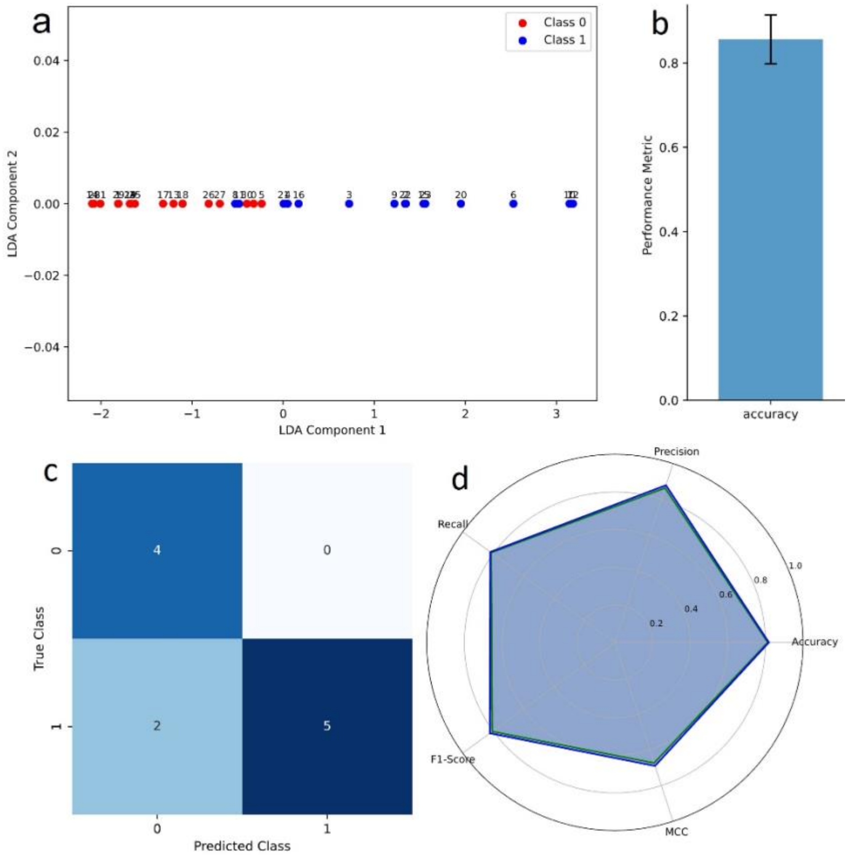

Fig. S26. Linear discriminant analysis after PCA dimension reduction of *Prototaxites* (class 0) vs Fungi (class 1). The ordination score plot **(a)** shows a slight overlap along the LDA first component axis. The bootstrap stability analysis **(b)** shows a very good resistance to changes in the dataset (mean= 0.86, std= 0.06). The confusion matrix **(c)** reports an excellent classification of the test set, with all *Prototaxites* correctly discriminated and only two fungi samples misclassified. The performance metrics **(d)** are excellent, with a training set (green) matching the test set (blue). This model can be considered robust, although the false negatives (the two misclassified fungi) and the MCC scores could potentially be improved.

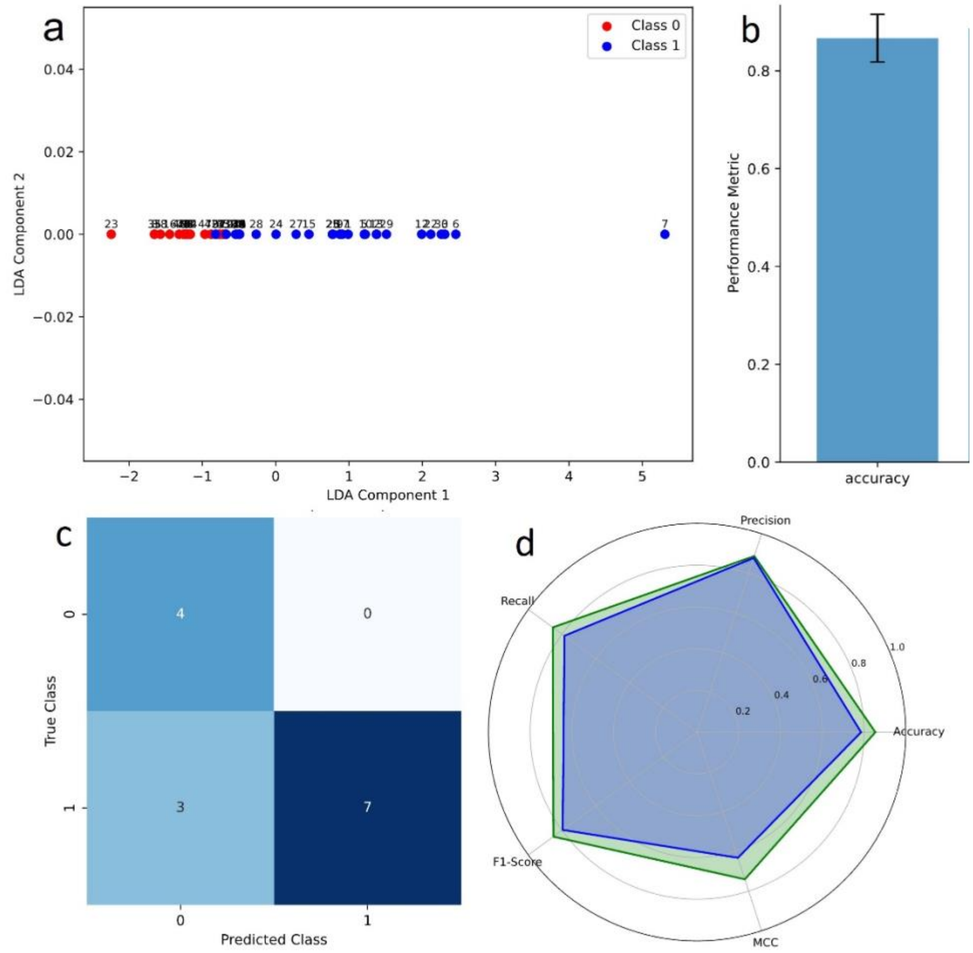

Fig. S27. Linear discriminant analysis after PCA dimension reduction of *Prototaxites* (class 0) vs Chitinous Organisms (class 1). The ordination score plot **(a)** shows a slight overlap along the LDA first component axis. The bootstrap stability analysis **(b)** shows a very good resistance to changes in the dataset (mean= 0.87, std= 0.05). The confusion matrix **(c)** reports an excellent classification of the test set with all *Prototaxites* correctly discriminated and only three chitinous samples misclassified. The performance metrics **(d)** are good, with a training set (green) performing slightly better than the test set (blue). This model can be considered robust, although the false negatives (the three misclassified chitinous organisms) and the MCC scores could potentially be improved.

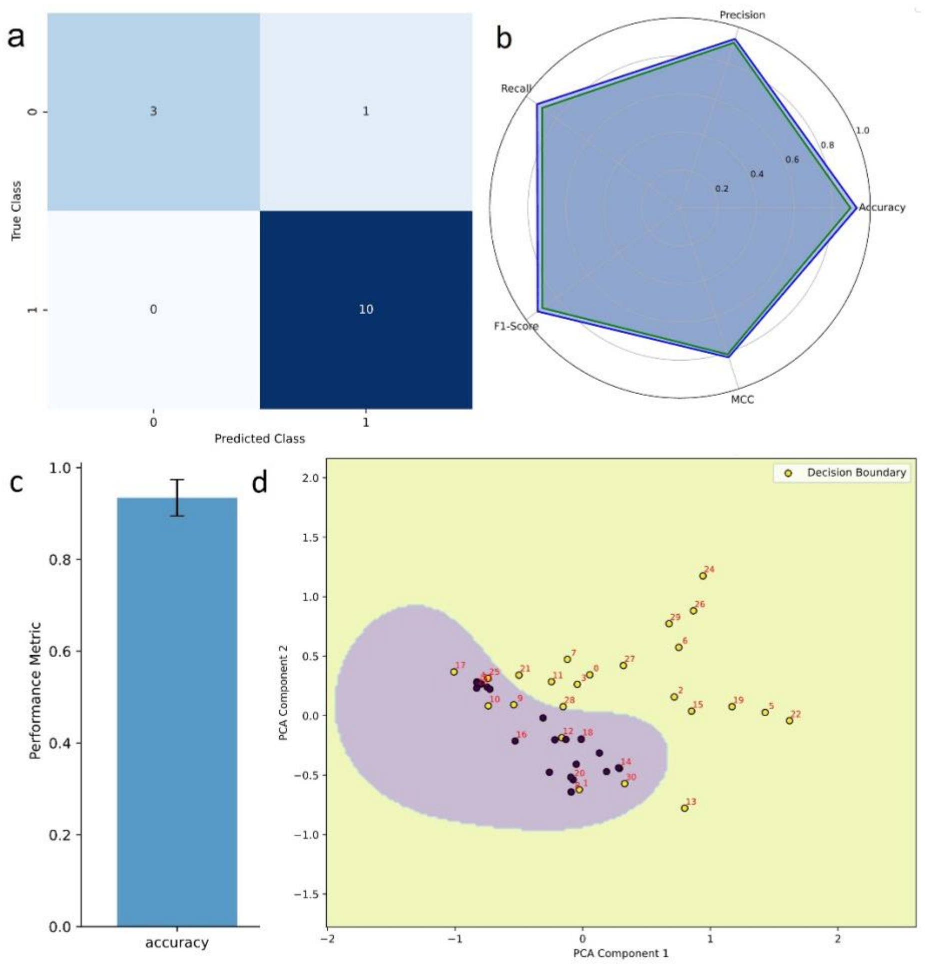

Fig. S28. Support Vector Machine analysis after PCA dimension reduction of *Prototaxites* (class 0) vs Plant (class 1). The confusion matrix **(a)** shows an excellent classification of the test set with all plant samples correctly discriminated, and only one *Prototaxites* sample misclassified. The performance metrics **(b)** are excellent, with the training set (green) matching the test set (blue). The bootstrap stability analysis **(c)** shows an excellent resistance to changes in the dataset (mean= 0.93, std= 0.4). **(d)** shows the decision boundary in two-dimensions for the C and gamma value parameter selected by the grid-search (C=10 and gamma = 1). This model can be considered very robust and shows a clear improvement over the LDA.

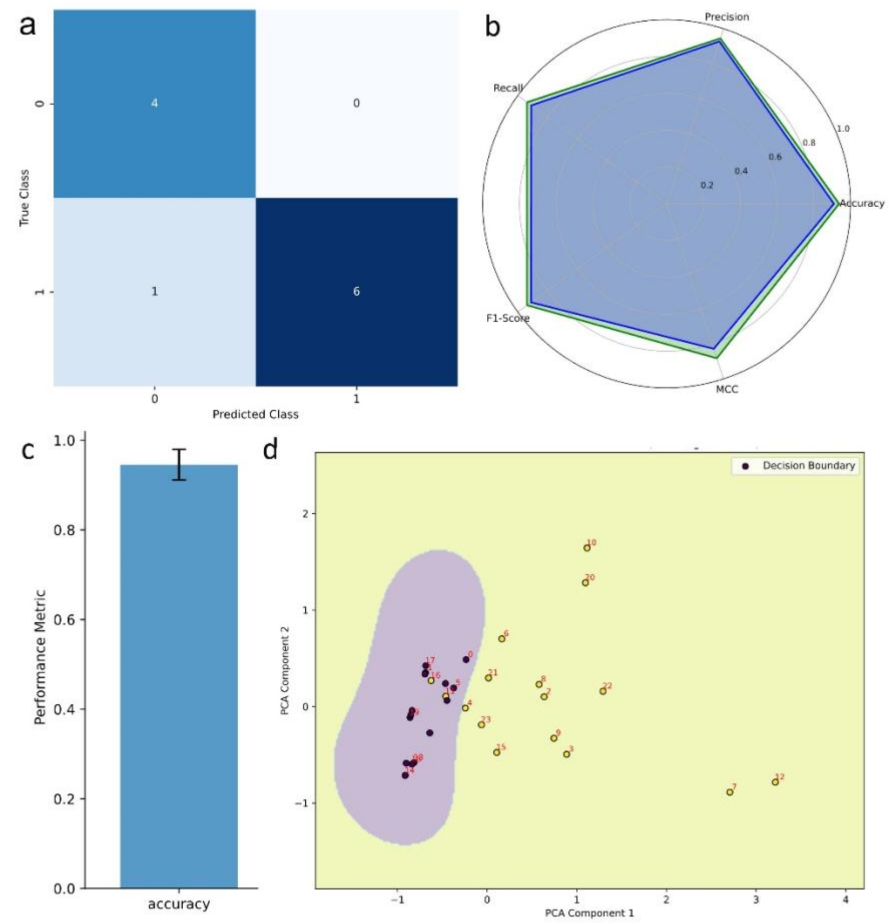

Fig. S29. Support Vector Machine analysis after PCA dimension reduction of *Prototaxites* (class 0) vs Fungi (class 1). The confusion matrix **(a)** shows an excellent classification of the test set, with all *Prototaxites* samples correctly discriminated and only one fungi sample misclassified. The performance metrics **(b)** are excellent, with the training set (green) closely matching the test set (blue). The bootstrap stability analysis **(c)** shows an excellent resistance to changes in the dataset (mean= 0.94, std= 0.03). **(d)** shows the decision boundary in two-dimensions for the C and gamma value parameters selected by the grid-search (C=10 and gamma = 1). This model can be considered very robust and shows a clear improvement over the LDA.

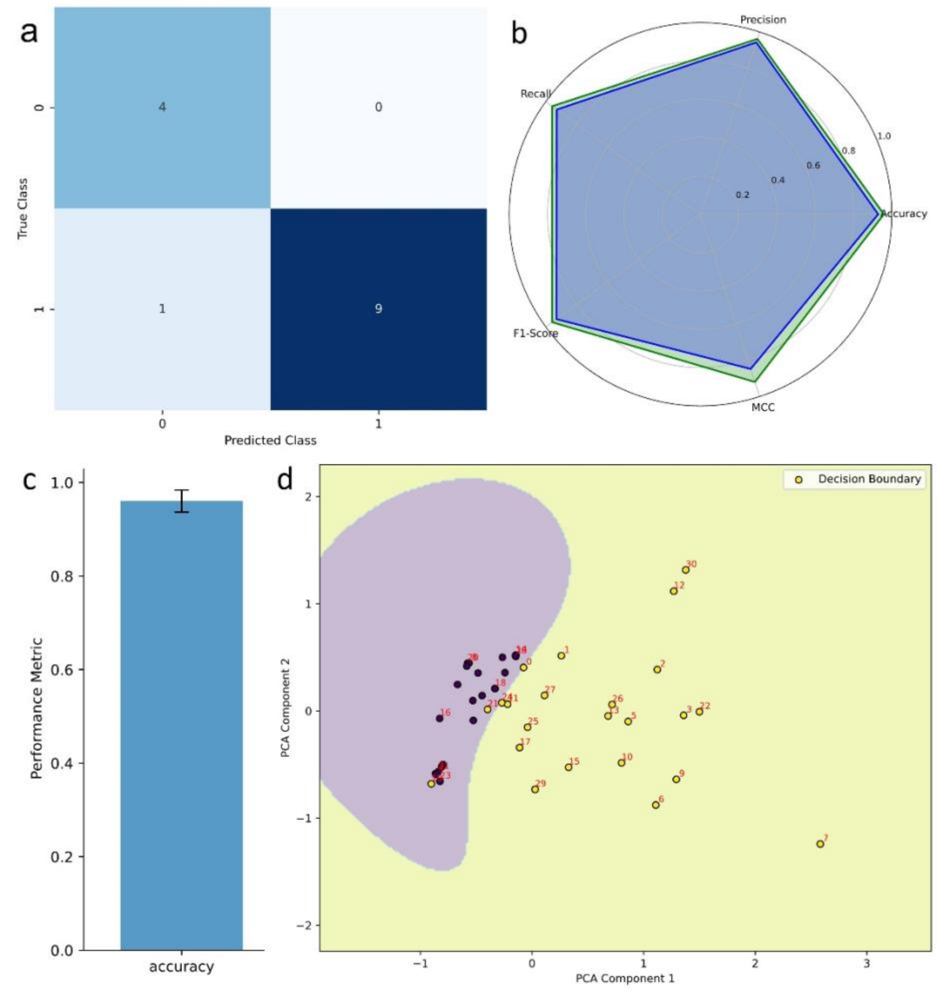

Fig. S30. Support Vector Machine analysis after PCA dimension reduction of *Prototaxites* (class 0) vs Chitinous Organisms (class 1). The confusion matrix **(a)** shows an excellent classification of the test set, with all *Prototaxites* samples correctly discriminated and only one chitinous organisms sample misclassified. The performance metrics **(b)** are excellent, with the training set (green) closely matching the test set (blue). The bootstrap stability analysis **(c)** shows an excellent resistance to changes in the dataset (mean= 0.96, std= 0.02). **(d)** shows the decision boundary in two-dimensions for the C and gamma value parameters selected by the grid-search (C=10 and gamma = 1). This model can be considered very robust and shows a clear improvement over the LDA.

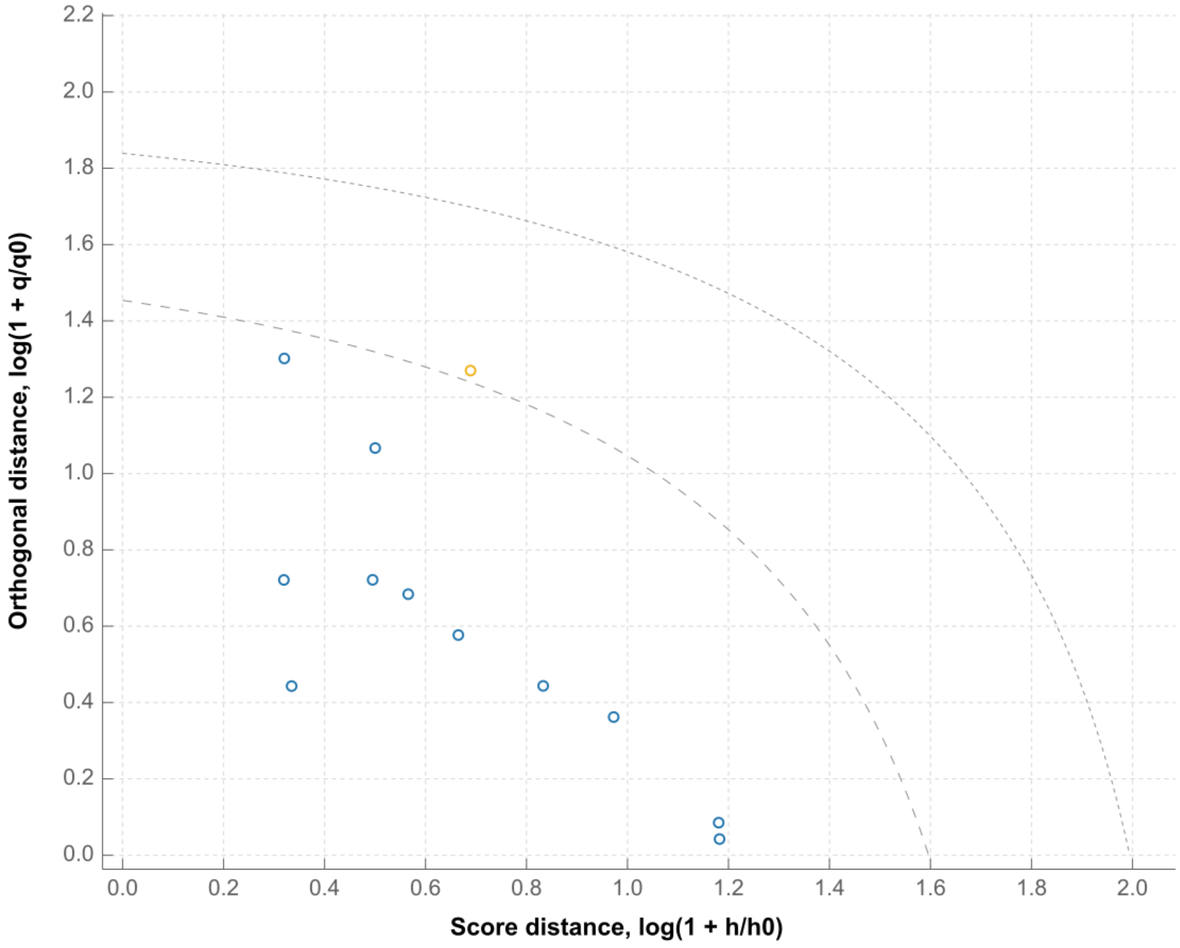

Fig. S31. Log-acceptance plot for the training set (*Prototaxites*). The dashed line delimits the regular area, and the dotted line delimits the extreme area, beyond which is the outlier space. All instances are recognised as regular (blue), except one instance which is extreme (orange).

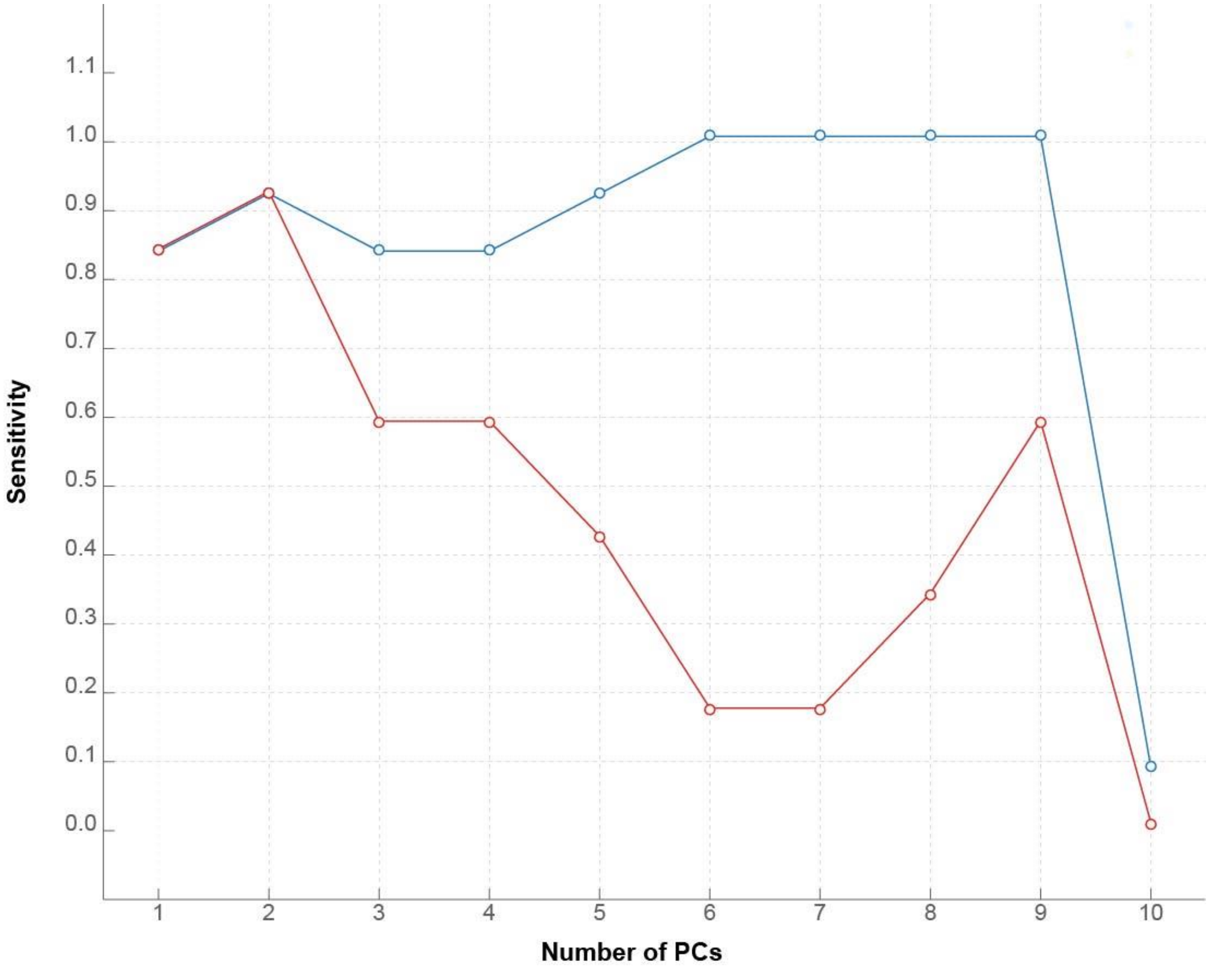

Fig. S32. Sensitivity plot for the training set (*Prototaxites*). The cross-validation performance is in red, and the training performance is in blue. A strong difference in performance between the two indicates a less robust model (here, from three PCs onward). The best sensitivity for both sets is achieved at two PCs.

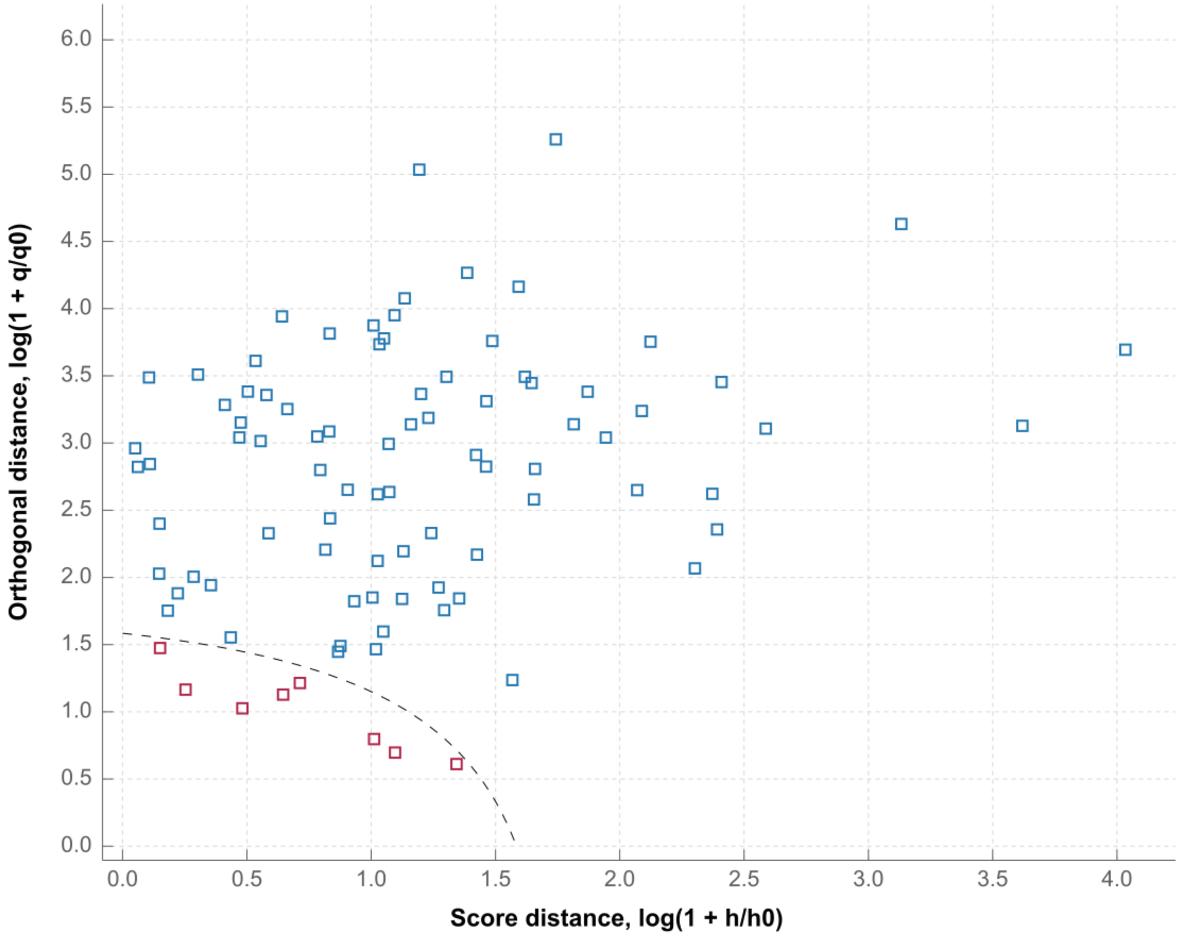

Fig. S33. Log-acceptance plot for our test set (all other Rhynie chert fossils). Eights instances are mistakenly identified as inlier/*Prototaxites* (red) out of 90 instances (7 plants and 1 arthropod sample).

Number	Band (cm^{-1})	Assignement
1	2958-2963	Asymmetric ν(CH_3)
2	2922-2926	Asymmetric ν(CH_2)
3	2888-2926	ν(CH)
4	2851-2853	Symmetric ν(CH_2)
5	1710-1725	ν(COOH); ν(C=O)
6	1684	Si-O
7	1650	ν(C=O); ν(C=N); (Amide I)
8	1614	Si-O
9	1595-1605	ν(C=C)
10	1570-1575	ν(C=N); COOH; carboxylate
11	1560	ν(C-N); δ(N-H); COOH; (Amide II)
12	1535-1540	ν(C-N); δ(N-H); COOH; carboxylate
13	1524	Si-O
14	1490	Si-O
15	1456-1464	δ(CH_2); δ(C-H)
16	1372	O-H (phenol); δ(CH_3)
17	1335	C-O (phenol), δ(C-H)
18	1260-1270	C-O (phenol)
19	1185	C-O-C; C-O/C-C; ν(C-N); aromatic δCH in plane
20	1120	C-O-C; C-O/C-C; aromatic δCH in plane
21	1030-1040	C-O/C-C

Table S1. FTIR band assignments. Stretching vibrations are indicated by the Greek letter ν, deformation vibrations by the Greek letter δ. Assignments are based on (*39, 47, 77-82*). Numbers correspond to numbers in **Figs. S7 and S8**.

Auxiliary supplementary materials

Datasets for FTIR analyses: raw spectra and datasets for each statistical analysis, with and without outliers. Dataset for CCA analyses.

Images of FTIR fossil targets: Images of sampled fossils. List of specimens, coordinates and repositories. Specimen codes: 2829p: Oxford University Museum of Natural History. Rfa, Rfe,

Rff, Rfg, ALG2, AGLyon 2019, AGL127, Agl75 2019-1, Lyon 48 UC1, Lyon 156 UCB1: University of Aberdeen. NMSRC9, G.2024.5.3, G.2024.5.5: National Museums of Scotland.

REFERENCES

1. F. M. Hueber, Rotted wood–alga–fungus: The history and life of *Prototaxites* Dawson 1859. *Rev. Palaeobot. Palynol.* **116**, 123–158 (2001).

2. W. Carruthers, On the history, histological structure, and affinities of *Nematophycus logani* Carr. (*Prototaxites logani* Dawson), an alga of Devonian age. *Mon. Microsc. J.* **8**, 160–172 (1872).

3. D. P. Penhallow, On *Nematophyton* and allied forms from the Devonian of Gaspé, with introductory notes by Sir William Dawson. *Transation of the Royal Society of Canada* (1889), vol. 6, pp. 27–47.

4. D. P. Penhallow, Notes on *Nematophyton crassum*. *Proc. United States National Museum* **16**, 115–118 (1893).

5. J. W. Dawson, On the fossil plants from the Devonian rocks of Canada. *Quart. J. Geol. Soc. London* **15**, 477–488 (1859).

6. R. Honegger, D. Edwards, L. Axe, C. Strullu-Derrien, Fertile *Prototaxites taiti*: A basal ascomycete with inoperculate, polysporous asci lacking croziers. *Philos. Trans. R. Soc. Lond. B Biol. Sci.* **373**, 20170146 (2018).

7. M. P. Nelsen, C. K. Boyce, What to do with *Prototaxites*? *Int. J. Plant Sci.* **183**, 556–565 (2022).

8. W. H. Lang, IV-On the plant-remains from the Downtonian of England and Wales. *Philos. Trans. R. Soc. Lond. B Biol. Sci.* **227**, 245–291 (1937).

9. L. E. Graham, M. E. Cook, D. T. Hanson, K. B. Pigg, J. M. Graham, Structural, physiological, and stable carbon isotopic evidence that the enigmatic Paleozoic fossil *Prototaxites* formed from rolled liverwort mats. *Am. J. Bot.* **97**, 268–275 (2010).

10. S. Chitaley, On the occurrence of *Prototaxites* in the Cleveland Black Shale of Ohio, USA. *Rev. Palaeobot. Palynol.* **72**, 257–271 (1992).

11. C. K. Boyce, C. L. Hotton, *Prototaxites* was not a taphonomic artifact. *Am. J. Bot.* **97**, 1073 (2010).

12. T. N. Taylor, E. L. Taylor, A.-L. Decombeix, A. Schwendemann, R. Serbet, I. Escapa, M. Krings, The enigmatic Devonian fossil Prototaxitesis not a rolled-up liverwort mat: Comment on the paper by Graham et al. (AJB97: 268–275). *Am. J. Bot.* **97**, 1074–1078 (2010).

13. C. K. Boyce, C. L. Hotton, M. L. Fogel, G. D. Cody, R. M. Hazen, A. H. Knoll, F. M. Hueber, Devonian landscape heterogeneity recorded by a giant fungus. *Geology* **35**, 399–402 (2007).

14. E. A. Hobbie, C. K. Boyce, Carbon sources for the Palaeozoic giant fungus *Prototaxites* inferred from modern analogues. *Proc. R. Soc. B* **277**, 2149–2156 (2010).

15. N. D. Burgess, D. Edwards, A new Palaeozoic plant closely allied to *Prototaxites* Dawson. *Bot. J. Linn. Soc.* **97**, 189–203 (1988).

16. G. D. Abbott, G. Ewbank, D. Edwards, G. Y. Wang, Molecular characterization of some enigmatic Lower Devonian fossils. *Geochim. Cosmochim. Acta* **62**, 1407–1418 (1998).

17. M. A. Selosse, *Prototaxites*: A 400 myr old giant fossil, a saprophytic holobasidiomycete, or a lichen? *Mycol. Res.* **106**, 642–644 (2002).

18. G. J. Retallack, E. Landing, Affinities and architecture of Devonian trunks of *Prototaxites loganii*. *Mycologia* **106**, 1143–1158 (2014).

19. M. A. Selosse, C. Strullu-Derrien, Origins of the terrestrial flora: A symbiosis with fungi? *BIO Web Conf.* **4**, 00009 (2015).

20. V. Vajda, L. Cavalcante, K. Palmgren, A. Krüger, M. Ivarsson, *Prototaxites* reinterpreted as mega-rhizomorphs, facilitating nutrient transport in early terrestrial ecosystems. *Can. J. Microbiol.* **69**, 17–31 (2022).

21. D. Edwards, L. Axe, Evidence for a fungal affinity for *Nematasketum*, a close ally of *Prototaxites*. *Bot. J. Linn. Soc.* **168**, 1–18 (2012).

22. M. L. Berbee, C. Strullu-Derrien, P. M. Delaux, P. K. Strother, P. Kenrick, M. A. Selosse, J. W. Taylor, Genomic and fossil windows into the secret lives of the most ancient fungi. *Nat. Rev. Microbiol.* **18**, 717–730 (2020).

23. C. Strullu-Derrien, T. Goral, A. R. T. Spencer, P. Kenrick, M. C. Aime, E. Gaya, D. L. Hawksworth, A fungal plant pathogen discovered in the Devonian Rhynie Chert. *Nat. Commun.* **14**, 7932 (2023).

24. D. Edwards, P. A. Selden, The development of early terrestrial ecosystems. *Bot. J. Scotl.* **46**, 337–366 (1992).

25. D. Edwards, P. A. Selden, L. Axe, Selective feeding in an Early Devonian terrestrial ecosystem. *Palaios* **27**, 509–522 (2012).

26. D. Edwards, L. Axe, J. L. Morris, L. Boddy, P. Selden, Further evidence for fungivory in the Lower Devonian (Lochkovian) of the Welsh Borderland, UK. *PalZ* **94**, 603–618 (2020).

27. R. Kidston, W. H. Lang, XXXIII.—On Old Red Sandstone plants showing structure, from the Rhynie Chert Bed, Aberdeenshire. Part V. The Thallophyta occurring in the peat-bed; the succession of the plants throughout a vertical section of the bed, and the conditions of accumulation and preservation of the deposit. *Earth Environ. Sci. Trans. R. Soc. Edinb.* **52**, 855–902 (1921).

28. D. S. Edwards, Studies on the Flora of the Rhynie Chert. University College of South Wales and Monmouthshire, Cardiff (1973).

29. E. J. H. Corner, Ad Polyporaceas VII: The xanthochroic polypores. *Beihefte zur Nova Hedwigia* **101**, 16–17 (1991).

30. E. J. H. Corner, A *Fomes* with two systems of hyphae. *Trans. Br. Mycol. Soc.* **17**, 51–81 (1932).

31. D. L. Porter, S. E. Naleway, Hyphal systems and their effect on the mechanical properties of fungal sporocarps. *Acta Biomater.* **145**, 272–282 (2022).

32. M. Kondas, Nematophytes. *Geol. Today.* **34**, 73–78 (2018).

33. C. T. Ingold, *Fungal Spores. Their Liberation and Dispersal* (Clarendon Press, Oxford, 1971), pp. 147–149.

34. R. Falck, Die Meruliusfäule des Bauholzes, in A. Moller ed. Hausschwammforschungen, sechstes heft. Jena: Gustav Fischer, Tafel XIII, 10. (1912).

35. D. E. G. Briggs, Molecular taphonomy of animal and plant cuticles: Selective preservation and diagenesis. *Philos. Trans. R. Soc. Lond. B Biol Sci.* **354**, 7–17 (1999).

36. S. Killops, V. Killops, *Introduction to Organic Geochemistry* (Blackwell Publishing, Oxford, ed. 2, 2005).

37. A. Stankiewicz, D. E. G. Briggs, R. P. Evershed, M. B. Flannery, M. Wuttke, Preservation of chitin in 25-million-year-old fossils. *Science* **276**, 1541–1543 (1997).

38. G. D. Cody, N. S. Gupta, D. E. G. Briggs, A. L. D. Kilcoyne, R. E. Summons, F. Kenig, R. E. Plotnick, A. C. Scott, Molecular signature of chitin-protein complex in Paleozoic arthropods. *Geology* **39**, 255–258 (2011).

39. C. C. Loron, E. Rodriguez Dzul, P. J. Orr, A. V. Gromov, N. C. Fraser, S. McMahon, Molecular fingerprints resolve affinities of Rhynie chert organic fossils. *Nat. Commun.* **14**, 1387 (2023).

40. Y. Qu, A. Engdahl, S. Zhu, V. Vajda, N. McLoughlin, Ultrastructural heterogeneity of carbonaceous material in ancient cherts: Investigating biosignature origin and preservation. *Astrobiology* **15**, 825–842 (2015).

41. L. J. Preston, M. J. Genge, The Rhynie Chert, Scotland, and the search for life on Mars. *Astrobiology* **10**, 549–560 (2010).

42. G. D. Abbott, I. W. Fletcher, S. Tardio, E. Hack, Exploring the geochemical distribution of organic carbon in early land plants: A novel approach. *Philos. Trans. R. Soc. Lond. B Biol. Sci.* **373**, 20160499 (2018).

43. A. I. Holman, S. F. Poropat, P. F. Greenwood, R. Bhandari, M. Tripp, P. Hopper, A. Schimmelmann, L. Brosnan, W. D. A. Rickard, K. Wolkenstein, K. Grice, Significance of lignin and fungal markers in the Devonian (407 Ma) Rhynie Chert. *Geobiology* **22**, e12616 (2024).

44. C. K. Boyce, G. D. Cody, M. L. Fogel, R. M. Hazen, C. M. O. D. Alexander, A. H. Knoll, Chemical evidence for cell wall lignification and the evolution of tracheids in early Devonian plants. *Int. J. Plant Sci.* **164**, 691–702 (2003).

45. V. E. McCoy, J. Wiemann, J. C. Lamsdell, C. D. Whalen, S. Lidgard, P. Mayer, H. Petermann, D. E. Briggs, Chemical signatures of soft tissues distinguish between vertebrates and invertebrates from the Carboniferous Mazon Creek Lagerstätte of Illinois. *Geobiology* **18**, 560–565 (2020).

46. J. Wiemann, J. M. Crawford, D. E. G. Briggs, Phylogenetic and physiological signals in metazoan fossil biomolecules. *Sci. Adv.* **6**, eaba6883 (2020).

47. C. C. Loron, M. C. Sforna, F. Borondics, C. Sandt, E. J. Javaux, Synchrotron FTIR investigations of kerogen from Proterozoic organic-walled eukaryotic microfossils. *Vib. Spectrosc.* **123**, 103476 (2022).

48. S. C. Watkinson, L. Boddy, N. Money, *The fungi* (Academic Press, 2015).

49. M. Kacurakova, P. Capek, V. Sasinkova, N. Wellner, A. Ebringerova, FT-IR study of plant cell wall model compounds: Pectic polysaccharides and hemicelluloses. *Carbohydr. Polym.* **43**, 195–203 (2000).

50. G. Perna, M. Lasalvia, V. Capozzi, Vibrational spectroscopy of synthetic and natural eumelanin. *Polym. Int.* **65**, 1323–1330 (2016).

51. M. Igisu, Y. Ueno, M. Shimojima, S. Nakashima, S. M. Awramik, H. Ohta, S. Maruyama, Micro-FTIR spectroscopic signatures of bacterial lipids in Proterozoic microfossils. *Precambrian Res.* **173**, 19–26 (2009).

52. C. L. Morais, L. M. Lima, M. Singh, F. L. Martin, Tutorial: Multivariate classification for vibrational spectroscopy in biological samples. *Nat. Protoc.* **15**, 2143–2162 (2020).

53. B. K. Baludikay, C. François, M. C. Sforna, J. Beghin, Y. Cornet, J. Y. Storme, E. J. Javaux, Raman microspectroscopy, bitumen reflectance and illite crystallinity scale: Comparison of different geothermometry methods on fossiliferous Proterozoic sedimentary basins (DR Congo, Mauritania and Australia). *Int. J. Coal Geol.* **191**, 80–94 (2018).

54. M. Igisu, T. Yokoyama, Y. Ueno, S. Nakashima, M. Shimojima, H. Ohta, S. Maruyama, Changes of aliphatic C–H bonds in cyanobacteria during experimental thermal maturation in the presence or absence of silica as evaluated by FTIR microspectroscopy. *Geobiology* **16**, 412–428 (2018).

55. A. Khiralla, A. O. Mohammed, S. Yagi, Fungal perylenequinones. *Mycol. Prog.* **21**, 38 (2022).

56. A. H. Knoll, The multiple origins of complex multicellularity. *Annu. Rev. Earth Planet. Sci.* **39**, 217–239 (2011).

57. L. G. Nagy, G. M. Kovács, K. Krizsán, Complex multicellularity in fungi: Evolutionary convergence, single origin, or both? *Biol. Rev.* **93**, 1778–1794 (2018).

58. T. Y. James, M. L. Berbee, No jacket required – New fungal lineage defies dress code: Recently described zoosporic fungi lack a cell wall during trophic phase. *Bioessays* **34**, 94–102 (2012).

59. T. A. Richards, G. U. Leonard, J. G. Wideman, What defines the "kingdom" fungi? *Microbiol. Spectr.* **5**, 10.1128/microbiolspec.funk-0044-2017 (2017).

60. M. Leménager, J. Burkiewicz, D. J. Schoen, S. Joly, Studying flowers in 3D using photogrammetry. *New Phytol.* **237**, 1922–1933 (2023).

61. J. Schindelin, I. Arganda-Carreras, E. Frise, V. Kaynig, M. Longair, T. Pietzsch, S. Preibisch, C. Rueden, S. Saalfeld, B. Schmid, J. Y. Tinevez, Fiji: An open-source platform for biological-image analysis. *Nat. Methods* **9**, 676–682 (2012).

62. M. D. Sutton, R. J. Garwood, D. J. Siveter, D. J. Siveter, SPIERS and VAXML: A software toolkit for tomographic visualisation and a format for virtual specimen interchange. *Palaeontol. Electron.* **15**, 14 (2012).

63. K. Grey, A modified palynological preparation technique for the extraction of large Neoproterozoic acanthomorph acritarchs and other acid-soluble microfossils (1999).

64. M. Toplak, S. T. Read, C. Sandt, F. Borondics, Quasar: Easy machine learning for biospectroscopy. *Cells* **10**, 2300 (2021).

65. M. Toplak, G. Birarda, S. Read, C. Sandt, S. M. Rosendahl, L. Vaccari, J. Demšar, F. Borondics, Infrared orange: Connecting hyperspectral data with machine learning. *Synchrotron Radiat. News* **30**, 40–45 (2017).

66. R. Bispo, F. Marques, Stability of principal components under normal and non-normal parent populations and different covariance structures scenarios. *J. Stat. Comput. Simul.* **93**, 1060–1076 (2023).

67. K. A. Bakeev, *Process Analytical Technology: Spectroscopic Tools and Implementation Strategies for the Chemical and Pharmaceutical Industries* (John Wiley & Sons, 2010).

68. C. J. ter Braak, Correspondence analysis of incidence and abundance data: Properties in terms of a unimodal response model. *Biometrics* **41**, 859–873 (1985).

69. C. J. ter Braak, Canonical correspondence analysis: A new eigenvector technique for multivariate direct gradient analysis. *Ecology* **67**, 1167–1179 (1986).

70. J. Oksanen, G. Simpson, F. Blanchet, R. Kindt, P. Legendre, P. Minchin, R. O'Hara, P. Solymos, M. Stevens, E. Szoecs, H. Wagner, M. Barbour, M. Bedward, B. Bolker, D. Borcard, T. Borman, G. Carvalho, M. Chirico, M. De Caceres, S. Durand, H. Evangelista, R. FitzJohn, M. Friendly, B. Furneaux, G. Hannigan, M. Hill, L. Lahti, C. Martino, D. McGlinn, M. Ouellette, E. Ribeiro Cunha, T. Smith, A. Stier, C. Ter Braak, J.Weedon, vegan: Community Ecology Package. R package version 2.8-0 (2025).

71. rpy2 – R in Python, v 3.6.1 (2025); https://rpy2.github.io/index.html.

72. F. Pedregosa, G. Varoquaux, A. Gramfort, V. Michel, B. Thirion, O. Grisel, M. Blondel, P. Prettenhofer, R. Weiss, V. Dubourg, J. Vanderplas, Scikit-learn: Machine learning in Python. *J. Mach. Learn. Res.* **12**, 2825–2830 (2011).

73. N. V. Chawla, K. W. Bowyer, L. O. Hall, W. P. Kegelmeyer, SMOTE: Synthetic minority over-sampling technique. *J. Artif. Intell. Res.* **16**, 321–357 (2002).

74. H. J. Butler, L. Ashton, B. Bird, G. Cinque, K. Curtis, J. Dorney, K. Esmonde-White, N. J. Fullwood, B. Gardner, P. L. Martin-Hirsch, M. J. Walsh, Using Raman spectroscopy to characterize biological materials. *Nat. Protoc.* **11**, 664–687 (2016).

75. S. Kucheryavskiy, O. Rodionova, A. Pomerantsev, A comprehensive tutorial on Data-Driven SIMCA: Theory and implementation in web. *J. Chemometr.* **38**, e3556 (2024).

76. A. L. Pomerantsev, O. Y. Rodionova, Concept and role of extreme objects in PCA/SIMCA. *J. Chemometr.* **28**, 429–438 (2014).

77. R. Lin, G. P. Ritz, Studying individual macerals using ir microspectrometry, and implications on oil versus gas/condensate proneness and "low-rank" generation. *Org. Geochem.* **20**, 695–706 (1993).

78. R. Lin, G. P. Ritz, Reflectance FT-IR microspectroscopy of fossil algae contained in organic-rich shales. *Appl. Spectrosc.* **47**, 265–271 (1993).

79. J. Coates, Interpretation of infrared spectra, a practical approach. *Encycl. Anal. Chem.* **12**, 10815–10837 (2000).

80. J. Arana, E. P. Melián, V. R. López, A. P. Alonso, J. D. Rodríguez, O. G. Díaz, J. P. Pena, Photocatalytic degradation of phenol and phenolic compounds: Part I. Adsorption and FTIR study. *J. Hazard. Mater.* **146**, 520–528 (2007).

81. C. Popescu, C. Vasile, M. Popescu, G. Singurel, V. I. Popa, B. S. Munteanu, Analytical methods for lignin characterization II. Spectroscopic studies. *Cellul. Chem. Technol.* **40**, 597–621 (2006).

82. G. F. Mohsin, F. J. Schmitt, C. Kanzler, J. D. Epping, S. Flemig, A. Hornemann, Structural characterization of melanoidin formed from d-glucose and l-alanine at different temperatures applying FTIR, NMR, EPR, and MALDI-ToF-MS. *Food Chem.* **245**, 761–767 (2018).

83. E. E. Bray, E. D. Evans, Distribution of n-paraffins as a clue to recognition of source beds. *Geochim. Cosmochim. Acta* **22**, 2–15 (1961).

84. B. M. Didyk, B. R. T. Simoneit, S. C. Brassell, G. Eglinton, Organic geochemical indicators of palaeoenvironmental conditions of sedimentation. *Nature* **272**, 216–222 (1978).

85. T. O. Akinsanpe, S. A. Bowden, J. Parnell, Molecular and mineral biomarker record of terrestrialization in the Rhynie Chert. *Palaeogeogr. Palaeoclimatol. Palaeoecol.* **640**, 112101 (2024).

86. K. Grice, H. Lu, P. Atahan, M. Asif, C. Hallmann, P. Greenwood, E. Maslen, S. Tulipani, K. Williford, J. Dodson, New insights into the origin of perylene in geological samples. *Geochim. Cosmochim. Acta* **73**, 6531–6543 (2009).

87. J. Rouillard, M. van Zuilen, C. Pisapia, J. M. Garcia-Ruiz, An alternative approach for assessing biogenicity. *Astrobiology* **21**, 151–164 (2021).

88. C. W. Hsu, C. C. Chang, C. J. Lin, A practical guide to support vector classification. [Preprint] (2003); https://www.csie.ntu.edu.tw/~cjlin/papers/guide/guide.pdf.

89. A. L. Pomerantsev, Acceptance areas for multivariate classification derived by projection methods. *J. Chemometr.* **22**, 601–609 (2008).
